# Hidden Allies: Decoding the Core Endohyphal Bacteriome of *Aspergillus fumigatus*

**DOI:** 10.1111/1758-2229.70153

**Published:** 2025-08-19

**Authors:** Daryna Piontkivska, João M. P. Jorge, Dalila Mil‐Homens, Tiago M. Martins, Pedro Crespo, Demosthenes P. Morales, Dinah Carvalho, José Melo‐Cristino, Raquel Sá‐Leão, Gustavo H. Goldman, Cristina Silva Pereira

**Affiliations:** ^1^ Instituto de Tecnologia Química e Biológica António Xavier Universidade Nova de Lisboa (ITQB NOVA) Oeiras Portugal; ^2^ Institute for Bioengineering and Biosciences (iBB) and Institute for Health and Bioeconomy (i4HB), Instituto Superior Técnico University of Lisbon Lisboa Portugal; ^3^ Department of Bioengineering, Instituto Superior Técnico University of Lisbon Lisboa Portugal; ^4^ Center of Integrated Nanotechnologies, Los Alamos National Laborator Los Alamos New Mexico USA; ^5^ Laboratory of Microbiology Unidade Local de Saúde Santa Maria Lisboa Portugal; ^6^ Faculdade de Ciências Farmacêuticas de Ribeirão Preto Universidade de São Paulo São Paulo Brazil; ^7^ National Institute of Science and Technology in Human Pathogenic Fungi São Paulo Brazil

**Keywords:** *Aspergillus fumigatus*, clinical isolates, endohyphal bacteria, endosymbionts, fungal bacteriome, fungal virulence

## Abstract

Bacterial–fungal interactions that influence the behaviour of one or both organisms are common in nature. Well‐studied systems include endosymbiotic relationships that range from transient to long‐term associations. Diverse endohyphal bacteria associate with fungal hosts, emphasising the need to better comprehend the fungal bacteriome. We evaluated the hypothesis that *Aspergillus fumigatus* harbours an endohyphal community of bacteria that influence the host phenotype. We analysed whether 38 *A. fumigatus* strains show stable association with diverse endohyphal bacteria; all derived from single‐conidium cultures that were subjected to antibiotic and heat treatments. The fungal bacteriome, inferred through analysis of bacterial diversity within the fungal strains (short‐ and long‐ read sequencing methods), revealed the presence of core endohyphal bacterial genera. Microscopic analysis further confirmed the presence of endohyphal bacteria. The fungal strains exhibited high genetic diversity and phenotypic heterogeneity in drug susceptibility and in vivo virulence. No correlations were observed between genomic or functional traits and bacteriome diversity, but the abundance of some bacterial genera correlated with fungal virulence or posaconazole susceptibility. The observed endobacteriome may play functional roles, for example, nitrogen fixation. Our study emphasises the existence of complex interactions between fungi and endohyphal bacteria, possibly impacting the phenotype of the fungal host, including virulence.

## Graphical abstract

*Aspergillus fumigatus* , a widespread fungal pathogen commonly found in soil, harbours a diverse endohyphal bacterial community. Microscopic analyses, including transmission electron microscopy, confirm bacterial presence within fungal hyphae. Identifying core bacterial genera highlights the need to further explore fungal–bacterial interactions and their potential impact on fungal biology and pathogenicity.

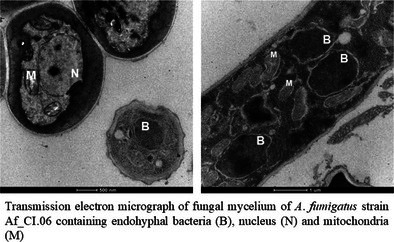

## 1 Background

Globally, new estimates indicate that over 6.5 million people are affected by invasive fungal infections and chronic pulmonary aspergillosis, with an overall crude mortality of nearly 3.8 million, of which 68% corresponds to the attributed mortality (Firacative 2020; Bongomin et al. 2017; Denning 2024). Recognising the need for combating fungal infections, the World Health Organization (WHO) identified four fungal priority pathogens, namely *Aspergillus fumigatus* , *Cryptococcus neoformans* , *Candidozyma auris* (previously *Candida auris*) and *Candida albicans* (Fisher and Denning 2023). *A. fumigatus* is widespread in both its geographic and ecological distribution, and it is the primary causative agent of invasive pulmonary aspergillosis—a particularly dangerous condition for immunocompromised individuals (Latge and Chamilos 2019). Furthermore, the escalating prevalence of antifungal resistance and the growing diversity of fungal species that are overcoming host‐specific barriers due to climate change create major threats to human health (Fisher et al. 2020). This threat is further accentuated by the finding that environmental pollutants increase the production of virulent *A. fumigatus* airborne spores (Martins et al. 2023). The combination of all of these factors increases the complexity of treating fungal infections and emphasises the need for reinforced vigilance and innovative strategies for clinical management (Evans et al. 2024).

Significant progress has been made in recent years toward understanding the genetic, molecular and ecological factors that determine the pathogenic potential of *A. fumigatus* (Paulussen et al. 2017; Abad et al. 2010). Studies have revealed that the virulence of this species is intricately modulated by a variety of factors, including variations in secondary metabolite production (Steenwyk et al. 2020), cell wall composition (Latge et al. 2017) and expression levels of virulence‐associated genes (Abad et al. 2010). Moreover, genetic polymorphisms in key regulatory genes and signalling pathways have been implicated in shaping the virulence profiles of different *A. fumigatus* strains (Abad et al. 2010; Steenwyk et al. 2020), stressing the complex genetic architecture that underlies pathogenicity. Unlike phytopathogenic fungi, for which specific virulence factors often dictate pathogenicity (Dort et al. 2023), the genetic and ecological distinctions that separate pathogenic *A. fumigatus* strains from their nonpathogenic counterparts remain unknown (Rokas 2022). Studies have demonstrated that genetically identical conidia (i.e., asexual spores) exhibit substantial phenotypic diversity—this has been reported for multiple different fungal species, including *A. fumigatus* (Wang et al. 2021). The observed phenotypic plasticity raises questions about the identity of environmental cues that may play a crucial role in shaping the pathogenic potential of *A. fumigatus* .

Numerous studies have shown an increasingly intricate interplay between fungi and their bacterial counterparts. While traditionally viewed as independent axenic entities, fungi and bacteria are now known to coexist within the same microbial communities (Urbanová et al. 2015; Kapitan et al. 2019). Bacteria can inhabit the internal spaces of fungal hyphae, either as transient colonisers, such as *Ralstonia solanacearum* invading *Aspergillus flavus* hyphae during co‐culture (Spraker et al. 2016), or as facultative endohyphal residents, as seen in several *Fusarium* spp. where bacteria can be isolated and may confer growth‐promoting traits, but without evidence of stable or essential symbiosis (Fang et al. 2023; Cheng et al. 2022). In contrast, some bacterial–fungal partnerships, such as the obligate mutualism between *Rhizopus microsporus* and its endohyphal *Burkholderia* spp., exhibit a highly integrated relationship where the fungus is fully dependent on the bacterium for its reproduction and virulence (Partida‐Martinez et al. 2007). These endohyphal bacteria can modulate various aspects of fungal biology, including growth, metabolism and virulence (Partida‐Martinez et al. 2007), through direct physical interactions or through the secretion of bioactive molecules. Furthermore, it was suggested that endohyphal bacterial associates in fungi appear to be the rule rather than the exception, as demonstrated by the examination of 700 phylogenetically diverse fungal isolates, including more than 50 environmental *Aspergillus* strains (Robinson et al. 2021). However, to date, our understanding of the functional complexity of endohyphal bacteria in clinically relevant fungi remains limited.

We addressed the hypothesis that *A. fumigatus* strains harbour endobacterial partners that can influence their hosts' phenotypes. To test this hypothesis, we systematically analysed the presence of endobacteria in a set of *Aspergillus* spp. clinical isolates (*n* = 40), mostly *A. fumigatus* (*n* = 37) and one environmental (soil) *A. fumigatus* isolate. Bacteriome profiling was undertaken using short‐ and long‐read sequencing approaches. All *A. fumigatus* strains were phenotypically characterised in terms of their drug susceptibility and virulence profiles, and a subset was visualised microscopically. Statistical analyses were used to explore potential correlations between specific endohyphal bacterial partners and the key phenotypic traits of the fungal host. Finally, bioinformatics methods were used to predict the functional niche space within the genetic pool of the endobacteria community. The structure of the core bacteriome, and its implications for the fungal host, are discussed.

## 2 Material and Methods

### 2.1 Study Design

In this study we tested the hypothesis that the bacterial partners of *A. fumigatus* strains contribute to their hosts' phenotypic variability. Fungal isolates were recovered from patients at the Hospital Santa Maria (HSM) in Lisbon, Portugal; one isolate, originating from soil, was also included in the study (Martins et al. 2023). Each strain was subjected to nuclear ribosomal Internal Transcribed Spacer (ITS) sequencing (Martins et al. 2023) for taxonomic classification (Zhang et al. 2000; Morgulis et al. 2008) (Table 1, Supporting Information 1) and the *A. fumigatus* strains to microsatellite genotyping (de Valk et al. 2005) (Supporting Information 2). The last data were used to infer the genetic diversity between strains, that is, minimum spanning network using Bruvo's distance, and compared with a subset of isolates from the databank for clinical and environmental *A. fumigatus* strains available at the afumID website (Sewell et al. 2019) through a discriminant analysis of principal components, which was performed using the R package poppr (v2.9.5) (Kamvar et al. 2014). Initially, bacteriomes were profiled in three randomly selected strains using a nested PCR 16S touchdown strategy with sequencing of the V4 region (Caporaso et al. 2011; Weisburg et al. 1991) (see processing details below). Based on the results, each fungal strain was then subjected to both antibiotic and thermal treatment (60°C, 1 h), followed by the isolation of a microcolony derived from a single conidium in solid medium (Nomani et al. 2018). Each individual conidium culture was propagated whenever fresh spores were required. The strains were genotyped and phenotypically analysed for antifungal susceptibility (EUCAST reference method; Subcommittee on Antifungal Susceptibility Testing of the ESCMID European Committee for Antimicrobial Susceptibility Testing 2008) and virulence (*Galleria mellonella* as the animal model; Martins et al. 2023). The endobacteriome of each strain was systematically analysed using V3‐V4 amplification (Thijs et al. 2017) (*n* = 38) and full length 16S MinIon sequencing (*n* = 9) of DNA extracted from 2‐day old mycelium, using multiple quality controls. In the last subset of fungal strains, the relative proportion of 18S:16S was also quantified in DNA extracted from fungal samples. In all bacteriome profiling analyses, the amplicon sequence variants (ASVs) (Callahan et al. 2017), computed using the DADA2 (v1.26.0) (Callahan et al. 2016) pipeline, were identified using the DECIPHER (v2.26.0) package with the IDTAXA algorithm (Murali et al. 2018). The closest bacterial hit from the SILVA SSU database r138 (Quast et al. 2013) was considered (Supporting Information 3). The 16S amplicons were clustered into OTUs (Blaxter et al. 2005) based on phylogeny‐derived distances, using the *tip_glom* function in the *phyloseq* R (v1.42.0) package (McMurdie and Holmes 2013), which were taxonomically identified against the SILVA SSU database r138 (Quast et al. 2013) as described above. The phylogenetic trees were inferred using the phangorn package (v2.11.1) (Schliep 2011), with sequence alignment generated by the msa package (v1.30.1) using the ClustalW method that identifies the best‐fitting model for our dataset (R scripts in the Supporting Information 4). Bioinformatic analyses always considered the relative abundances of each ASV at taxonomic levels down to the genus level. FAPROTAX (v1.2.10) and PICRUSt2 (v2.5.2) were used to predict the ecologically relevant functions of the microbiomes from the fungal isolates (Louca et al. 2016; Douglas et al. 2020). Complementary whole genome sequencing of fungal DNA was also performed (*n* = 2). Attempts to isolate bacteria in aerobic standard conditions were carried out as well. Finally, the bacterial partners inside the hyphae of 20‐h old mycelia were visualised through fluorescent microscopy analyses of mycelia grown in hydrogel media discs (Morales et al. 2022; Woo et al. 2010), and through transmission electron microscopy (TEM) analyses.

**TABLE 1:** Description of all *Aspergillus* isolates used in this study. The source of isolation and whether it originated from a cystic fibrosis (CF) patient are indicated. NCBI BLAST alignment of the sequenced ITS regions (amplicon sequences available in Supporting Information 1) with TYPE material sequences (accession numbers), along with identity percentages and gap counts, are provided (Cheng et al. 2022; Partida‐Martinez et al. 2007).

Isolate	Isolation source	CF patient	Scientific name	Accession	Identities	Gaps
**Af_SI.00**	Soil	—	*Aspergillus fumigatus*	NR_121481.1	504/504 (100%)	0
**Af_CI.01**	Sputum	Yes	*A. fumigatus*	NR_121481.1	562/563 (99%)	0
**Af_CI.02**	Sputum	Yes	*A. fumigatus*	NR_121481.1	592/592 (100%)	0
**Af_CI.03**	Sputum	Yes	*A. fumigatus*	NR_121481.1	587/587 (99%)	0
**Af_CI.06**	Sputum	Yes	*A. fumigatus*	NR_121481.1	591/591 (100%)	0
**Af_CI.07**	Sputum	Yes	*A. fumigatus*	NR_121481.1	571/571 (100%)	0
**Af_CI.08**	Sputum	Yes	*A. fumigatus*	NR_121481.1	578/579 (99%)	0
**Af_CI.09**	Sputum	Yes	*A. fumigatus*	NR_121481.1	572/572 (100%)	0
**Af_CI.11**	Ear Secs.	No	*A. fumigatus*	NR_121481.1	574/574 (100%)	0
**Af_CI.12**	Sputum	No	*A. fumigatus*	NR_121481.1	573/574 (99%)	0
**Af_CI.13**	BAL	No	*A. fumigatus*	NR_121481.1	572/573 (99%)	0
**Af_CI.14**	Sputum	Yes	*A. fumigatus*	NR_121481.1	577/577 (100%)	0
**Af_CI.15**	Sputum	Yes	*A. fumigatus*	NR_121481.1	586/586 (100%)	0
**Af_CI.16**	Sputum	Yes	*A. fumigatus*	NR_121481.1	572/572 (100%)	0
**Af_CI.17**	Sputum	Yes	*A. fumigatus*	NR_121481.1	577/577 (100%)	0
**Af_CI.18**	Sputum	Yes	*A. fumigatus*	NR_121481.1	575/575 (100%)	0
**Af_CI.19**	Sputum	Yes	*A. fumigatus*	NR_121481.1	585/585 (100%)	0
**Af_CI.20**	Sputum	No	*A. fumigatus*	NR_121481.1	584/584 (100%)	0
**Af_CI.22**	Sputum	Yes	*A. fumigatus*	NR_121481.1	579/579 (100%)	0
**Af_CI.23**	Sputum	Yes	*A. fumigatus*	NR_121481.1	580/580 (100%)	0
**Af_CI.24**	Sputum	Yes	*A. fumigatus*	NR_121481.1	574/575 (99%)	0
**Af_CI.27**	Sputum	Yes	*A. fumigatus*	NR_121481.1	555/555 (100%)	0
**Af_CI.28**	Sputum	No	*A. fumigatus*	NR_121481.1	582/584 (99%)	1
**Af_CI.30**	Sputum	Yes	*A. fumigatus*	NR_121481.1	589/590 (99%)	0
**Af_CI.31**	Sputum	Yes	*A. fumigatus*	NR_121481.1	572/572 (100%)	0
**Af_CI.32**	Lung Biopsy	No	*A. fumigatus*	NR_121481.1	582/583 (99%)	0
**Af_CI.34**	Sputum	Yes	*A. fumigatus*	NR_121481.1	588/589 (99%)	1
**Af_CI.36**	Sputum	Yes	*A. fumigatus*	NR_121481.1	574/574 (100%)	0
**Af_CI.37**	Sputum	Yes	*A. fumigatus*	NR_121481.1	590/590 (100%)	0
**Af_CI.38**	Sputum	Yes	*A. fumigatus*	NR_121481.1	530/531 (99%)	1
**Af_CI.39**	Sputum	Yes	*A. fumigatus*	NR_121481.1	597/598 (99%)	1
**Af_CI.41**	Sputum	Yes	*A. fumigatus*	NR_121481.1	588/589 (99%)	0
**Af_CI.42**	Sputum	Yes	*A. fumigatus*	NR_121481.1	583/584 (99%)	1
**Af_CI.43**	Sputum	Yes	*A. fumigatus*	NR_121481.1	588/588 (100%)	0
**Af_CI.44**	Sputum	Yes	*A. fumigatus*	NR_121481.1	526/529 (99%)	0
**Af_CI.45**	Sputum	No	*A. fumigatus*	NR_121481.1	518/518 (100%)	0
**Af_CI.46**	BAL	No	*A. fumigatus*	NR_121481.1	524/524 (100%)	0
**Af_CI.47**	Sputum	Yes	*A. fumigatus*	NR_121481.1	570/571 (99%)	0
**Afla_CI.04**	Sputum	Yes	*Aspergillus flavus*	KU729026.1	540/541 (99%)	1
**An_CI.05**	Bronchial Secs.	No	*Aspergillus niger*	NR_111348.1	559/562 (99%)	0
**At_CI.25**	Oropharyngeal Secs.	No	*Aspergillus terreus*	NR_131276.1	603/603 (100%)	0

Abbreviations: BAL, bronchoalveolar lavage; Secs., secretions.

Full description of the methods in Supporting Information 5.

## 3 Results

Our main working hypothesis is that endohyphal bacteria contribute to the phenotypic heterogeneity observed in *A. fumigatus* . To address this hypothesis, *Aspergillus* clinical isolates were primarily isolated from patients at HSM in Lisbon, Portugal and were taxonomically classified as *A. fumigatus* (*n* = 37), *Aspergillus terreus* , *Aspergillus niger* and *A. flavus* (*n* = 1 each) (Table 1). Additional strains used in some assays were an *A. fumigatus* soil isolate (Af_SI.00) (Martins et al. 2023) and the reference strain Af293.

### 3.1 Setting‐Up a Framework to Analyse Endohyphal Bacterial Partners of *A. fumigatus* Clinical Isolates

The presence of endohyphal bacterial associates was initially tested in three randomly selected *A. fumigatus* strains to address two outstanding questions: (i) if antibiotic treatment eliminates ephemeral bacterial associates; and (ii) if cultures generated from a single conidium display a lower diversity of bacterial associates than those derived from multiple conidia. Bacterial profiling was systematically performed by amplicon sequencing of the hypervariable region V4 of the 16S rRNA gene. The ASVs obtained were taxonomically identified through the IDTaxa algorithm (Murali et al. 2018) using the SILVA 16S database as a training set (Quast et al. 2013). Due to the low taxonomic resolution of the short‐read sequences analysed here, the bacterial ASVs identified are displayed at class or family level.

To evaluate the impact of antibiotic selection, the composition of the bacterial community in mycelia grown in media supplemented with high‐dose ciprofloxacin (a broad‐spectrum antibiotic) was compared to that from mycelia grown in media without antibiotic. A total of 233 unique ASVs were identified across the 6 samples examined (Supporting Information 5: Figure S2A). Among these, 30 ASVs were present in the mycelia that were not subjected to the antibiotic selection. This result suggests that these bacteria, which could be eliminated by treatment with antibiotic, are likely not stable endohyphal partners of *A. fumigatus* . One hundred and fifty‐six ASVs were only detected after antibiotic treatment, suggesting that the antibiotic selection induced a very noticeable shift in the composition of the bacterial community, allowing detection of less abundant bacteria. Interestingly, 47 ASVs were found at relative high abundances in mycelia cultivated in media both without and with antibiotic selection (Supporting Information 5: Figure S2A). This observation implies a strong association between these ASVs and mycelia, suggesting the existence of bacteria within or perhaps on hyphae.

**FIGURE 1:**
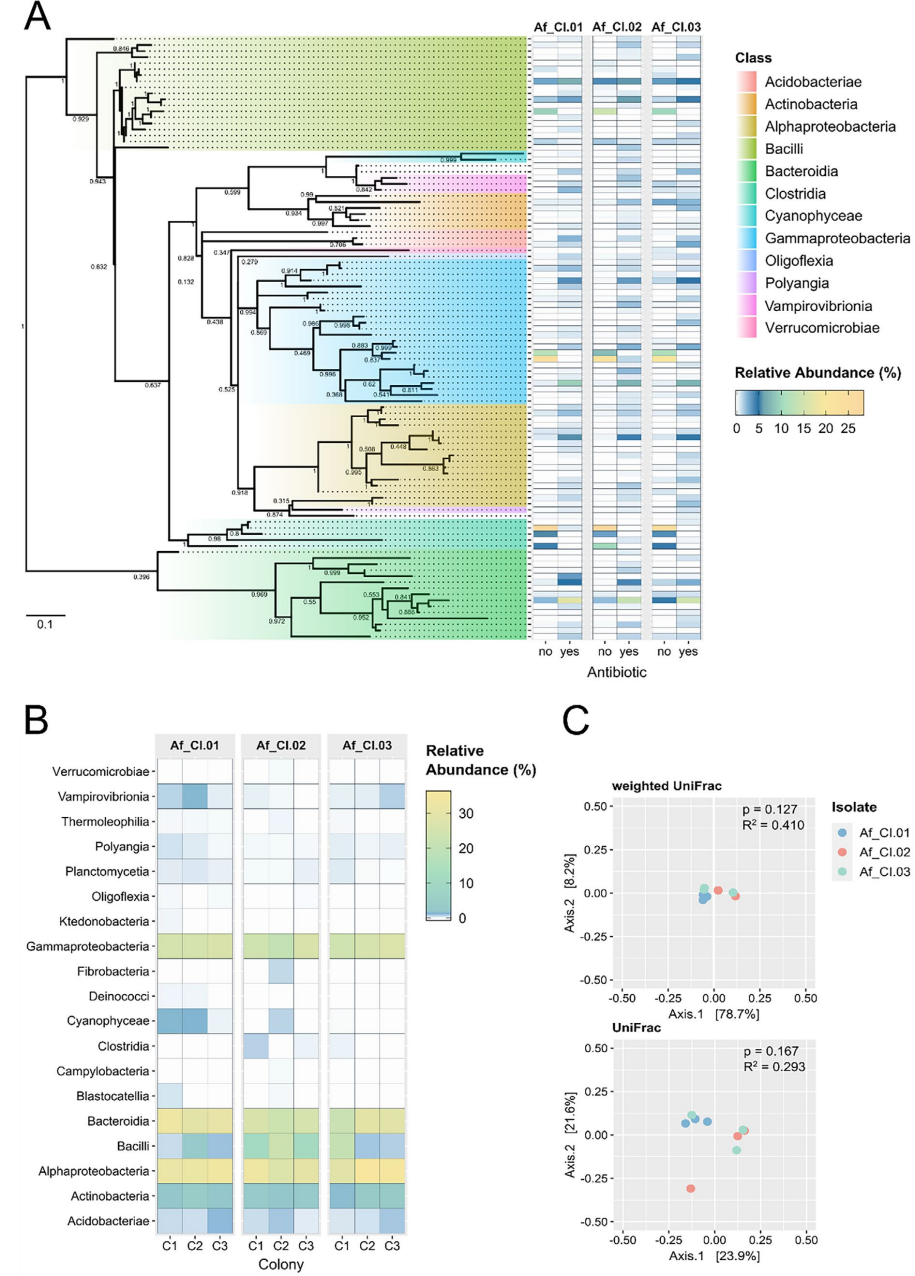
Bacteriome of three *Aspergillus fumigatus* strains grown with and without antibiotic stress in cultures derived from multiple conidia or single‐conidium. (A) Maximum likelihood midpoint rooted tree of the 100 most abundant bacterial ASVs across the sample set. The maximum likelihood tree was constructed using the general time reversible model with the rate variation among sites described by a gamma distribution and the proportion of invariable sites (GTR + G + I model). Background colours indicate bacterial ASVs assigned at class level. Tree constriction was based on the hypervariable V4 region of 16S rRNA gene sequences, applying 1000 bootstrap replications to estimate confidence. Bootstrap values are indicated above or below the branches. The scale bar indicates nucleotide substitutions per site. Heatmap shows the relative abundances of bacterial ASVs found in tested fungal clinical isolates without (no) and with (yes) antibiotic treatment. The colour intensity shows the ASV percentage in each sample (note that in the colour key the dark blue corresponds to 5%). (B) Heat‐map diagram bacteriome composition at class level of the 3 single spore colonies (marked as C1, C2 or C3) of the three tested fungal clinical isolates. (C) PCoA plot of beta diversity of the single spore cultures based on weighted and unweighted Unifrac distances.

A phylogenetic analysis focusing on the top 100 most abundant ASVs was undertaken (representing ~98% and ~92% of the total relative abundance in the absence and presence of antibiotic, respectively) to better understand their distribution across different fungal strains and conditions. The results highlighted that there were major alterations in the composition of the bacterial community postantibiotic selection, specifically a heightened community diversity with a clear dominance of ASVs belonging to the classes *Bacteroidia*, *Alphaproteobacteria* and *Polyangia* (Figure 1A). For a given fungal strain, no significant correlation was detected between the untreated control and the sample subjected to antibiotic selection. In contrast, a strong positive Pearson's correlation (*p* ≤ 0.0001) was identified among the three different fungal strains under the same conditions. These results suggest that the bacteriomes of the three fungal strains are similar under pre‐ or postantibiotic treatment, but not across conditions (Supporting Information 5: Figure S2B).

The complexity of the mycelial bacteriome led to the decision to isolate single‐conidium cultures—with the aim of eliminating any contribution from conidial heterogeneity to the overall bacterial diversity. Additionally, as some bacteria outside of conidia may survive the antibiotic treatment, conidia were also subjected to heat stress (60°C, 1 h) in addition to the antibiotic pressure. The conditions were optimised using two axenic bacteria cultures ( *Escherichia coli* and *Hydrobacter penzbergensis*). In contrast to some of the conidia, none of the bacteria could survive the heat treatment. We generated single‐conidium cultures (*n* = 3 per isolate) from three fungal isolates and analysed the associated bacteriomes. The results showed similar bacteriomes across the single‐conidium colonies from the same fungal strain and for the three different fungal strains tested (Figure 1B). The resulting heatmap highlights the similar bacterial community composition and the distribution of relative abundance across the sample set at the class level (Figure 1B). Further examination through phylogenetic analysis focusing on the top 100 most abundant bacterial ASVs (representing ~95% of the total abundance) showed consistent results: all single‐conidium colony cultures show a similar bacteriome, regardless of the parental fungal strain (Supporting Information 5: Figure S3). The same inference can be derived from beta diversity analyses based on both weighted and unweighted Unifrac distances—also showing the similarity of the bacteriomes analysed (Figure 1C).

Collectively, the results showed a remarkable similarity for the hyphal bacteriomes across three distinct fungal strains, inoculated from conidia subjected to heat treatment in an antibiotic selection medium. All subsequent experiments used a randomly selected single‐conidium colony per *Aspergillus* spp. strain.

### 3.2 Determining the Range of Genotypic Diversity and Phenotypic Heterogeneity of the *A. fumigatus* Clinical Isolates

**FIGURE 2:**
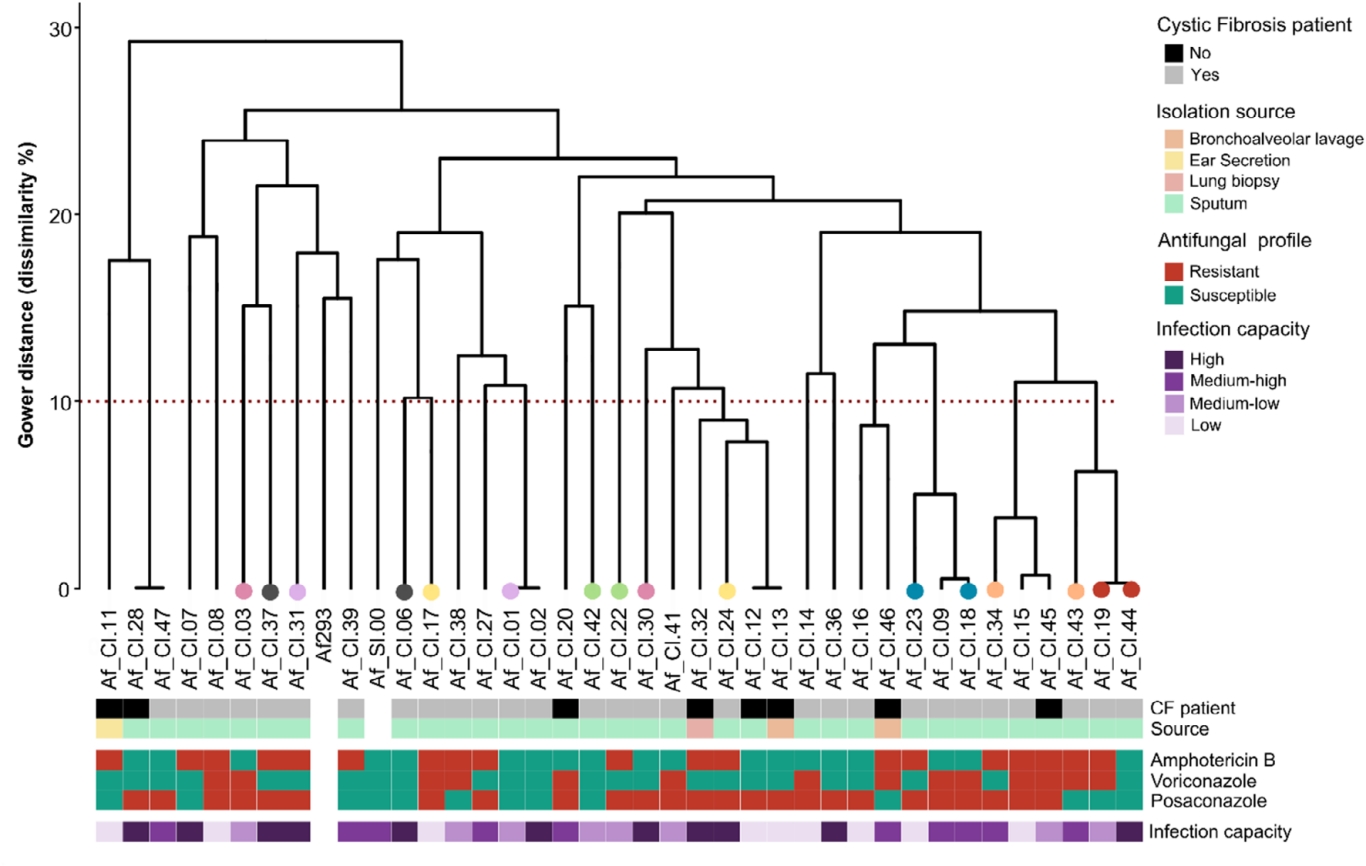
Microsatellite genotyping and phenotypic heterogeneity of 38 *Aspergillus fumigatus* strains assessed in terms of their drug‐resistance and virulence profiles. Hierarchical cluster dendrogram of microsatellite genotypes of *A. fumigatus* isolates constructed based on the Gower dissimilarity index, with the laboratory model strain Af293 and the soil isolate (Af_SI.00) for comparison. Dots at the end of the dendrogram indicate isolates obtained from the same patient (colour‐coded accordingly). The source of isolation and whether it originated from a cystic fibrosis patient are indicated below the dendrogram (detailed in Table 1). Antifungal susceptibility profiles, assessed via the EUCAST method, and in vivo infection capacity using *Galleria mellonella* as the infection model (96 h) are represented in the heatmap (Table S1).

Most of the *A. fumigatus* clinical isolates (*n* = 37 in total) originated from patients with cystic fibrosis (*n* = 29, 78%). Overall, the bulk of the samples were isolated from sputum; the exceptions were from ear secretions (*n* = 1), lung biopsies (*n* = 1) and bronchoalveolar lavage (*n* = 2). In some cases, two isolates were sourced from the same patient (Table 1, Figure 2).

We used microsatellite genotyping to achieve a higher discriminatory power within *A. fumigatus* (de Valk et al. 2005). First, we tested if the geographic origin of the *A. fumigatus* strains from HSM in Lisbon impacts on their genetic diversity. Therefore, our genotyping results (Supporting Information 2) were compared with those of clinical isolates found in other countries (Sewell et al. 2019). The minimum spanning network displaying the relatedness between the isolates showed that the 37 clinical strains exhibit considerable genetic variability (Supporting Information 5: Figure S4). This result suggests that genotypic diversity within isolates of *A. fumigatus* surpasses geographic boundaries, consistent with previous studies (Sewell et al. 2019). Distinct lineages within the *A. fumigatus* strains were identified through hierarchical cluster analysis using the Gower dissimilarity index (Figure 2). The strains Af293 (lab strain) and Af_SI.00 (isolated from soil) were included for comparison purposes. Clustering with a maximum dissimilarity threshold of 10% revealed the presence of 27 clusters, highlighting the strains' genotypic diversity. Four pairs of strains were found to be genotypically identical and just one of those pairs was isolated from the same patient (Af_CI.19 and Af_CI.44) (Figure 2).

We then analysed the phenotypic heterogeneity of *A. fumigatus* strains, in terms of their drug‐resistance and virulence profiles (*n* = 38). Minimal inhibitory concentrations (MICs) analysed by the EUCAST method (Subcommittee on Antifungal Susceptibility Testing of the ESCMID European Committee for Antimicrobial Susceptibility Testing 2008) ranged from 16 to 0.03 mg·L^−1^ for amphotericin B and voriconazole, and from 8 to 0.016 mg·L^−1^ for posaconazole (Supporting Information 5: Table S1). Overall, the results show significant heterogeneity in the drug‐susceptibility profiles, with no apparent clustering to their lineage as depicted in Figure 2.

In vivo infection capacity of each *A. fumigatus* strain was assessed using *G. mellonella* as the infection model (*n* = 38, plus the Af293 strain). This model is widely recognised for evaluating the virulence of microbial pathogens (Araujo et al. 2022), particularly fungal pathogens (Mil‐Homens et al. 2018), and demonstrates a reliable correlation with murine models (Slater et al. 2011). Following a 96‐h postinfection period, the fungal strains exhibited varying degrees of infection capacity. According to the survival probability range, the infection index was classified as low (> 75%); medium‐low (75%–50%); medium‐high (50%–25%) and high (< 25%) (Figure 2; Supporting Information 5: Figure S5). The virulence results did not exhibit any clustering with the isolates' lineages.

Collectively these results show that the 37 *A. fumigatus* clinical strains characterised in this study, sampled within close geographic proximity, exhibit substantial genotypic diversity and phenotypic heterogeneity in terms of their virulence and drug‐resistance profiles, consistent with previous reports (Dos Santos et al. 2020). Neither antifungal susceptibility nor genotyping lineage correlated with virulence potential.

### 3.3 Determining the Core Bacteriome of *A. fumigatus* Clinical Isolates

To identify the *A. fumigatus* core bacteriome, all 37 clinical strains and the Af_SI.00 strain were profiled through sequencing of the V3–V4 hypervariable region of the 16S rRNA gene. Prior to this analysis, we verified in a subset of strains (*n* = 9) that the relative proportions of 18S:16S quantified via RT‐*q*PCR were consistent in the DNA extracts of both spores and 48 h‐old mycelia (Supplementary 5: Table S2). Bacterial DNA is consistently present in all DNA samples derived from either fungal source (except for AF_CI.08 that presents a slow growth rate), ranging from 0.35% to 1.6% of bacterial DNA in the total DNA.

**FIGURE 3:**
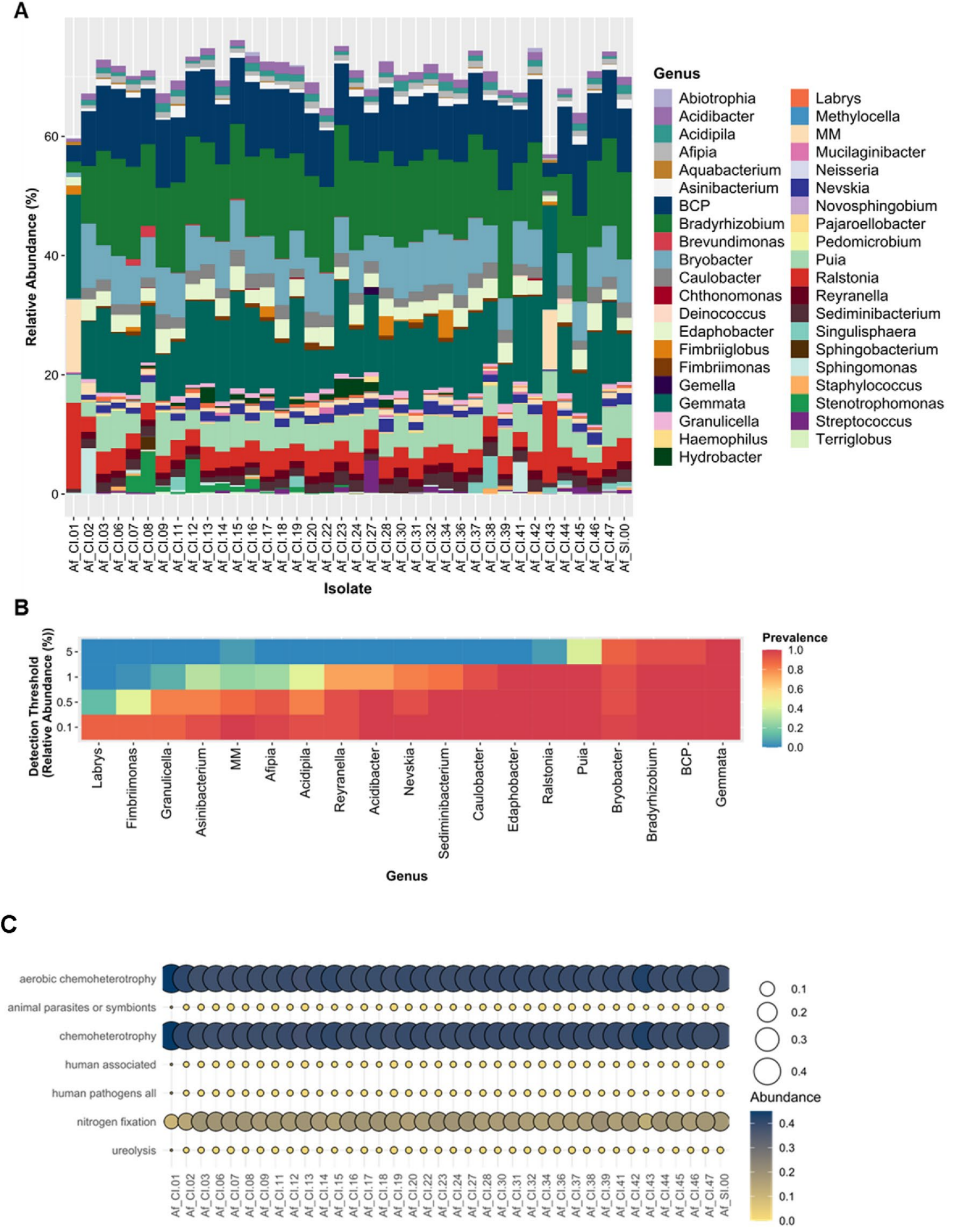
The core endohyphal bacteriome of the 38 *Aspergillus fumigatus* strains profiled through sequencing of the V3‐V4 hypervariable region of the 16S rRNA gene, and the derived functional annotation of prokaryotic taxa. (A) Stacked bar chart showing the relative abundance of ASVs from the bacterial V3‐V4 hypervariable region of 16S rRNA sequences, taxonomically classified at the genus level. Low abundance taxa were removed from the visualisation. The order of bacterial genus in the legend is according to its position in the chart. (B) Heat map of the core bacteria at the genus level across the sample set (*n* = 38), based upon 75% prevalence with at least a 0.1% detection threshold. The y‐axis represents the detection thresholds (indicated as relative abundance), colour shading indicates the prevalence of each bacterial genus among samples for each abundance threshold. (C) The annotation of prokaryotic taxa (FAPROTAX) predicted from the genetic pool of the core endobacteria (75% prevalence with at least 0.1% detection threshold) using the relative abundance ASVs from the bacterial V3‐V4 hypervariable region of 16S rRNA sequences, showing potential functional roles, mostly in categories such as chemoheterotrophy and nitrogen fixation, followed by human pathogens/associated and animal parasites or symbionts.

All analysed *A. fumigatus* strains (*n* = 38) exhibited a consistent bacteriome profile at the genus level (Figure 3A). This result suggests stability in the bacterial community across all tested fungal strains. Such stability expands to the bacteriome profile of the other clinical *Aspergillus* spp. strains, namely *A. terreus, A. niger* and *A. flavus* (*n* = 1, each) (Supporting Information 5: Figure S6). This observation supports the notion that the bacterial communities associated with distinct aspergilla originate from shared ecological niches and/or functional roles—this is deserving of a more focused analysis in the future.

Evaluation of prevalence at the genus level highlighted the presence of 19 core bacterial genera, of which 11 were present in all *A. fumigatus* strains (Figure 3B). Considering the relative abundance, *Gemmata* was the most prevalent genus (5%), followed by the *Burkholderia‐Caballeronia‐Paraburkholderia* (BCP) group, *Bradyrhizobium, Puia, Ralstonia* and *Edaphobacter* spp. (1%, each), *Acidibacter* and *Sediminibacterium* (0.5%, each) and the *Methylobacterium‐Methylorubrum* (MM) group, *Afipia* and *Nevskia* (< 0.1%) (Figure 3B). Most of the core bacterial genera match multiple ASVs identifications, likely due to the presence of closely related variants within each genus. Phylogenetic analysis of the core bacteriome suggests that these ASVs are closely related and likely represent the same bacterial species (Supporting Information 5: Figure S7). Importantly, bacteria detected in all quality controls (amplification and extraction negative controls) were systematically excluded from all bacteriome profiling analyses (Supporting Information 5: Figure S1).

**FIGURE 4:**
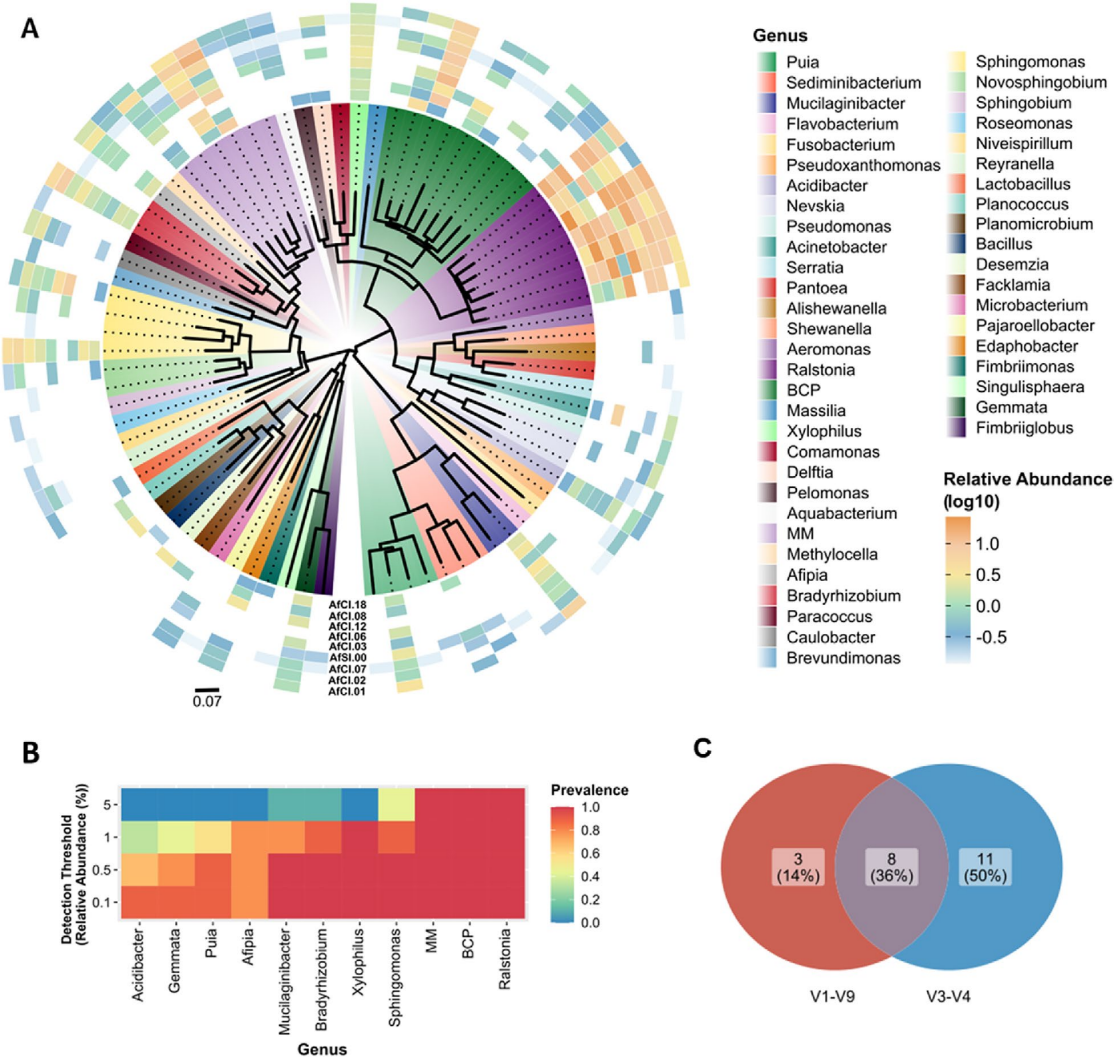
The core endohyphal bacteriome of 9 *Aspergillus fumigatus* strains profiled through long‐read sequencing of 16S rRNA amplicons. (A) Maximum likelihood tree of V1‐V9 16S rRNA gene bacterial ASVs identified at genus level, with the respective relative abundance (log_10_) in each *A. fumigatus* isolate. The genus order in the legend is the same as in the tree, counterclockwise. (B) Heat map of the core bacteria at genus level across the sample set (*n* = 9), based upon 75% prevalence with at least 0.1% detection threshold. The y‐axis represents the detection thresholds (indicated as relative abundance), colour shading indicates the prevalence of each bacterial genus among samples for each abundance threshold. (C) Venn diagram showing the number of ASVs at genus level found in common in both long length (V1‐V9) and short length (V3‐V4) analysis.

To further test the presence of a core bacteriome, a subset of *A. fumigatus* strains (*n* = 9, Supporting Information 5: Table S2) was profiled using long‐read sequencing of 16S rRNA amplicons on the Oxford Nanopore MinION platform. The utilisation of long‐read sequencing techniques offers a deeper insight into microbial communities (Tedersoo et al. 2021) and may potentially validate the identification performed using the shorter‐amplified sequences. This approach yielded a total of 49 bacterial genera (Supporting Information 3). However, the majority of these genera are associated with a few strains at remarkably low abundance (Figure 4A). The potential roles of rare bacteria in the fungal bacteriome certainly warrant further investigation.

In total, 11 core bacterial genera were identified, of which seven were found consistently across all nine isolates, including *Ralstonia*, the BCP group, the MM group, *Sphingomonas, Xylophilus, Bradyrhizobium* and *Mucilaginibacter* (Figure 4B). The two core bacteriomes identified have eight bacterial genera in common (Figure 4C). This result highlights the stability of certain bacterial genera across different sequencing methods.

Bacterial genera consistently identified within the core bacteriome may play key roles in the structure and function of this microbial community. Metabolic dependencies are thought to play a significant role in species co‐occurrence and are indicative of the presence of regular cooperative groups within microbial community architectures (Zelezniak et al. 2015). This concept also underlies the formation and structure of holobiont systems. Intercellular bacterial partners of eukaryotic hosts can provide primary metabolic pathways (e.g., photosynthesis) or expand the repertoire of secondary metabolism while also influencing the host's fitness, growth, development, behaviour and other functions (Kelliher et al. 2023; Araldi‐Brondolo et al. 2017). In this respect, most of the core endohyphal bacterial genera identified herein can be found in the soil habitat and demonstrate viability in acidic and low nutrient (oligotrophic) environments. Some are rare bacterial genera with only a few species known (e.g., *Gemmata*, *Puia*, *Acidibacter* and *Xylophilus*) (Dworkin et al. 2006a; Falagan and Johnson 2014; Lv et al. 2017; Dworkin et al. 2006b), while others are more common (e.g., *Ralstonia*). Many are known to form symbiotic relationships with eukaryotes, and their chemoheterotrophy suggests a potential for N fixation, for example *Ralstonia* (Chen et al. 2003; Itabangi et al. 2022), *Bryobacter* (Kulichevskaya et al. 2010), *Bradyrhizobium* (Wang et al. 2020; Camuel et al. 2023), the BCP group (Kaur et al. 2017) and *Sphingomonas* (Almeida et al. 2018). Based on the genetic pool of the endohyphal microbiota of *A. fumigatus* (inferred from the V3‐V4 amplicons), both the functional annotation of prokaryotic taxa (FAPROTAX) (Louca et al. 2016) and the metabolic space could be predicted (PICRUSt2) (Douglas et al. 2020; Fahimipour and Gross 2020), as applied in other related studies (Zheng et al. 2024). Despite the obvious limitation of relying only on genus‐level data, the results show potential functional roles, mostly in categories such as chemoheterotrophy and nitrogen fixation, followed by human pathogens/associated and animal parasites or symbionts (Figure 3C), as well as amino acid metabolism, lipid metabolism and cofactor, carrier and vitamin biosynthesis (Supporting Information 5: Figure S8).

As performed in other studies (Sharmin et al. 2018; Uehling et al. 2017; Meng et al. 2018), we subjected two *A. fumigatus* strains (Af_CI.002 and Af_CI.12) to whole‐genome sequencing (WGS). The acquired data revealed a very low abundance of sequences not matching *A. fumigatus* (0.36%–0.47%) with the majority unclassified. This result is consistent with that inferred through the relative abundance of 18S/16S amplified from fungal DNA samples (Supporting Information 5: Table S2). It is however relevant that a predominant bacterial genus could be identified: *Bradyrhizobium* (Supporting Information 5: Figure S9), with several sequences aligning, at distinct regions, with a reference genome of *Bradyrhizobium guangzhouense* (CCBAU 51670, acc. no. CP030053). This bacterial genus was consistently identified within the core bacteriome using other sequencing methods (Figures 3 and 4), hence further validating its presence within the mycelia. Sequencing the full genome of predominant endohyphal bacteria would allow a better understanding of the core endobacteriome. However, due to the low abundance of bacterial DNA, such future studies will require multiple rounds of optimization to identify cultivation conditions and DNA processing methods that result in higher yields of endobacterial DNA.

**FIGURE 5:**
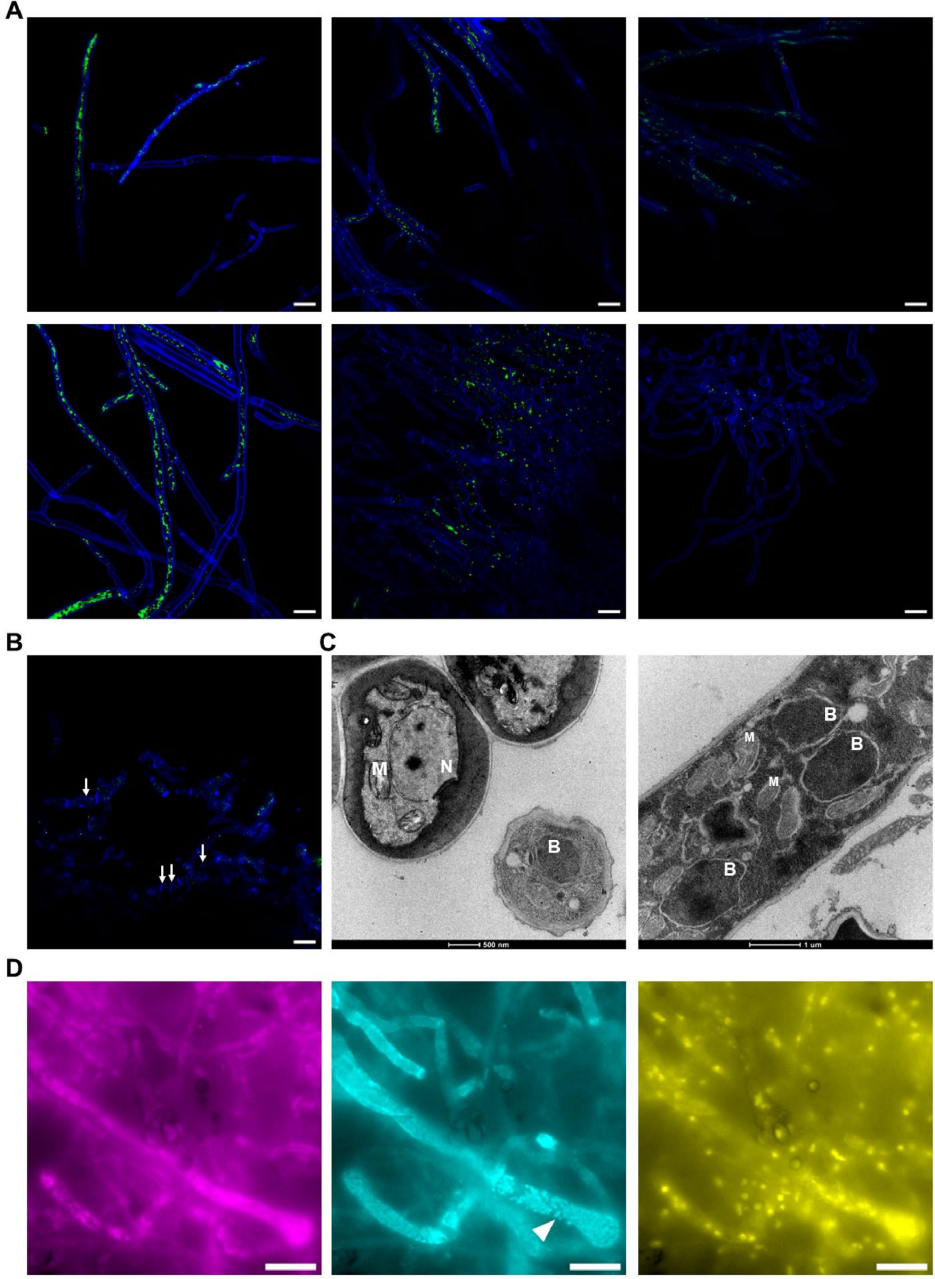
Visualisation of endohyphal bacteria in *Aspergillus fumigatus* strains by microscopy. (A) Fluorescent microscopy, where mycelia were stained with calcofluor‐white (blue) and bacterial DNA with syto9 (green) (Scale bar = 10 μm). Representatives photographs were selected, from left to right: Af_SI.00; Af_CI.01; Af_CI.02; Af_CI.03; Af_CI.12 and Af_CI.18. (B) Fluorescent microscopy, where mycelia were stained with calcofluor‐white (blue), bacterial DNA with syto9 (green) and fungal nuclei with Hoechst (blue) (Scale bar = 10 μm). A representative example is shown: Af_CI.12. The fungal nuclei labelling (↓) enables a clear distinction between fungal and bacterial DNA. (C) Transmission electron micrograph of fungal mycelium of *A. fumigatus* strain Af_CI.06 containing endohyphal bacteria (B), nucleus (N) and mitochondria (M). (D) Fluorescent in situ hybridization depicting clusters of spherical bacteria (cyan, marked with an arrow) along the hyphae of *A. fumigatus* (magenta). Fungal rRNA were labelled with the universal eukaryotic 18S rRNA probe; bacteria were co‐stained with a universal 16S rRNA probe and DAPI was used as a global nuclear staining (yellow) (Scale bar = 10 μm).

To further explore the fungal– bacterial association, endohyphal bacteria were visualised under the microscope. *A. fumigatus* 20 h‐old hyphae (*n* = 6, within those used for the long‐read sequencing analyses) were stained with styo9, a dye commonly used to label nucleic acids and especially effective in staining endobacteria within fungi (Hazarika et al. 2020; Shao et al. 2020). The endobacteria were observed within the hyphae of all analysed fungal strains (Figure 5A). None of these endohyphal bacteria could be cultured aerobically using standard growth protocols. Fungal nuclei, stained with Hoechst (blue), have a distinctive morphology and larger size than the syto9‐stained endobacteria (green) (Figure 5B). Bacterial staining was also confirmed by FISH with a universal 16S rRNA probe targeting the bacteria in the fungi co‐stained for the 18S rRNA. Clusters of spherical bacteria (cyan) were observed along the hyphae of *A. fumigatus* (magenta) (Figure 5D). TEM was used to verify the presence of endobacteria in one strain of *A. fumigatus* , as applied before by others (Sharma et al. 2008). The mycelium from solid culture was frozen at high pressure and cryosubstituted in osmium tetroxide and uranyl acetate. Examination of the samples confirmed the presence of endohyphal bacteria within the cytosol of intact cells of *A. fumigatus* (Figure 5C). The direct visualisation of endohyphal bacteria further validates the bacteriome profiling results. Further assays are needed to identify conditions that allow them to grow outside of the fungal host. The long‐read amplicons could match the identification of bacteria up to the species level, yet most identified species matched those of uncultivable bacteria (data not shown).

### 3.4 Identifying Endohyphal Bacteria That Potentially Impact the Virulence and Drug‐Resistance Profiles of *A. fumigatus* Clinical Isolates

**FIGURE 6:**
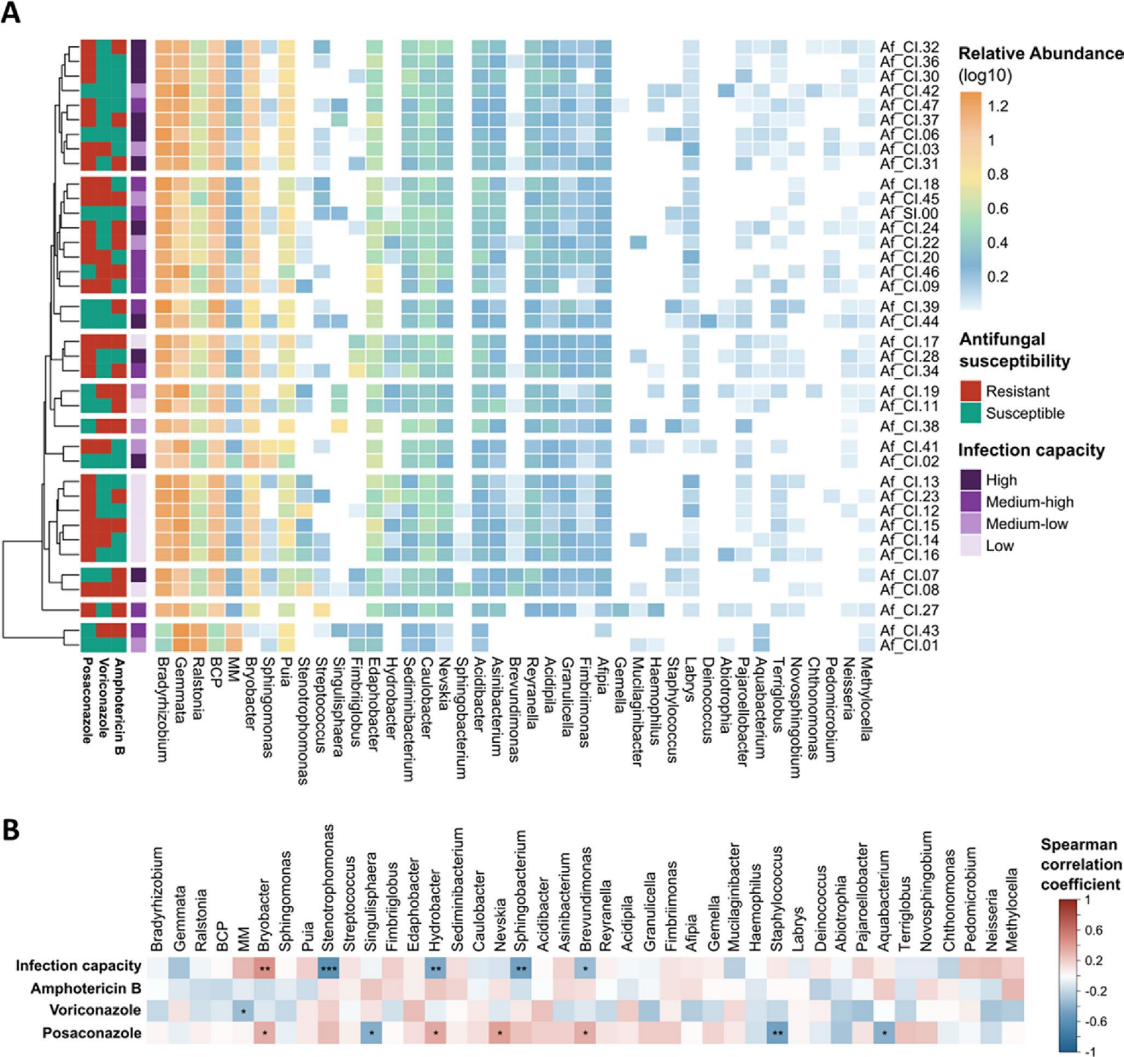
Identifying endohyphal bacteria potentially impacting the virulence and drug‐resistance profiles of *Aspergillus fumigatus* strains. (A) Hierarchical clustering heat map of fungal bacteriome using Bray–Curtis distance. Samples were clustered with maximum of 0.15 dissimilarity. (B) Spearman's correlation, asterisks indicate significant correlations *p ≤ 0.05; **p ≤ 0.01; ***p ≤ 0.001. Based on region V3–V4 of the 16S rRNA gene.

To test the hypothesis that the endohyphal bacteriome contributes to phenotypic heterogeneity of *A. fumigatus* virulence (i.e., in vivo infection capacity) and drug susceptibility, hierarchical clustering analyses were first applied using Bray‐Curtis distances. Based on the data, the fungal bacteriome (genus level) did not correlate with either virulence or drug susceptibility (Figure 6A, Supporting Information 5: Figure S10A). The same conclusion—bacterial diversity did not correlate with the fungal phenotype—could be inferred from testing only the bacterial genera that contribute to variations in the bacteriome, that is, only the rare endohyphal bacteria without the core bacteria (Supporting Information 5: Figure S10B). Finally, we tested if the abundance of specific core endohyphal bacteria shows correlation with the analysed fungal phenotypic traits using Spearman's correlation analyses (Figure 6B, Supporting Information 6). The results of the correlation coefficients (r_s_) suggest that endohyphal bacterial diversity did not correlate with susceptibility to either amphotericin B or voriconazole, with the exception of a negative correlation of MM abundance with voriconazole susceptibility. However, posaconazole susceptibility showed positive correlations with abundance of *Bryobacter* (*r*_s_ = 0.34), *Hydrobacter* (*r*_s_ = 0.34), *Nevskia* (*r*_s_ = 0.39) and *Brevundimonas* (*r*_s_ = 0.33), and negative correlations with the abundance of *Singulisphaera* (*r*_s_ = −0.41), *Aquabacterium* (*r*_s_ = −0.38) and *Staphylococcus*. This result suggests a protective mechanism possibly related to the bacterial chemoheterotrophy and ability to degrade the antifungal drug. Only *Bryobacter* exhibited a positive correlation (*r*_s_ = 0.45) with fungal virulence, possibly the genus abundance directly correlates with the in vivo infection capacity of the host. On the contrary, the abundance of either *Stenotrophomonas* (*r*_s_ = −0.58), *Hydrobacter* (*r*_s_ = −0.43), *Sphingobacterium* (*r*_s_ = −0.48) or *Brevundimonas* (*r*_s_ = −0.33) displayed a negative correlation with fungal virulence. Overall, the correlational analyses highlighted a possible relationship between the abundance of a specific core endohyphal bacteria genera of *A. fumigatus* and its virulence and drug susceptibility. Focused in‐depth analyses are needed to better understand how core endohyphal bacteria impact the phenotypic traits of the fungal host, especially those that are clinically relevant.

## 4 Discussion

We formulated a hypothesis that *A. fumigatus* strains harbour diverse core endohyphal bacteria that may contribute to its phenotypic heterogeneity. Amplicon sequencing of the 16S rRNA gene revealed a dynamic bacterial landscape, where antibiotic selection induced significant shifts in the composition of the community, with certain bacterial taxa persisting (Figure 1). This initial result suggests that some bacterial taxa exhibit more robust associations with their fungal hosts. Sequencing analyses, spanning both short‐ and long‐read platforms, of single conidium‐derived colonies of *A. fumigatus* strains (*n* = 38), which are genotypically diverse (Figure 2), highlighted a core bacteriome (Figures 3 and 4). This result makes it clear that conidial heterogeneity did not contribute to the bacteriome diversity observed. *A. fumigatus* strains (*n* = 38) displayed high phenotypic heterogeneity in their antifungal susceptibility and virulence profiles, without clear correlation with their genotypic diversity (Figure 2). These findings underscore, as is often reported, the multifaceted nature of *A. fumigatus* strains (Wang et al. 2021). Microscopy analysis visually confirmed the presence of endobacteria located within hyphae (Figure 5C). The role of the core bacteriome in expanding the nutrient assimilation capacity of *A. fumigatus*, as well as the ecological niches that it can occupy, remains hypothetical but is supported by the functional annotation of chemoheterotrophy and nitrogen fixation, among others (Figure 3C, Supporting Information 5: Figure S8). The remarkable diversity of the fungal bacteriome at taxonomic, functional and lifestyle levels has been reported in other studies (Robinson et al. 2021). Our data further challenge the paradigm of axenic fungi or limited association of fungi with only one (Partida‐Martinez et al. 2007) or two (Almeida et al. 2018) endohyphal bacteria.

Several observations support the presence of a core bacteriome. First, the genetic relationship of the fungal strains analysed was similar to those from distant geographic locations (Supporting Information 5: Figure S4) and was independent of proximity. Second, transient bacterial associates were efficiently removed through antibiotic pressure and heat‐shock (Figure 1). Third, single‐conidium cultures were consistently used in all assays to avoid heterogeneity within conidia populations (Figure 2). The phylogenies were inferred from sequences obtained using culture‐independent methods, ensuring an unbiased by cultivability representation of endohyphal bacterial diversity. Rigorous bioinformatics methods were employed, and potential sequencing artefacts from cross‐contaminants were systematically removed (Salter et al. 2014), in agreement with the highest standards for analysing complex metagenomes (Welsh and Eisenhofer 2024). Finally, the combined use of short‐ and long‐read platforms is recognised as the optimal strategy for generating robust datasets (Xu et al. 2022). The incorporation of the intragenomic variation found between 16S gene copies would ensure higher taxonomic resolution (Johnson et al. 2019) but is unachievable because the number of formally described taxa at any rank is insignificant compared with the total number of detected taxa (Yarza et al. 2014).

Symbiosis drives the acquisition of adaptive traits, ecological range expansion and biodiversity. The transition to obligate interspecific mutualism marks a major evolutionary step (West et al. 2015), seen in fungal hosts unable to replicate without their symbionts (Partida‐Martinez et al. 2007). While the roles of endohyphal bacteria in *A. fumigatus* are not fully determined, data suggest a close relationship between the fungi and their core bacterial associates. The presence of a core bacteriome across various aspergilla strains (*n* = 40) (Figures 3 and 4; Supporting Information 5: Figure S6) implies vertically transmitted endosymbionts. Gram‐negative bacteria dominate this core bacteriome (Figures 3 and 4). Fungi from all major phyla can harbour bacterial endosymbionts, primarily Gram‐negative, with obligate associations in early diverging fungi such as *Mucoromycota* and facultative associations in more derived lineages (Araldi‐Brondolo et al. 2017; Pawlowska et al. 2018). Examples of symbiosis include intracellular bacterial symbionts in insects providing essential nutrients (Dial et al. 2022), and the *R. microsporus* and *Burkholderia* spp. partnership, where bacteria produce rhizoxin to aid fungal pathogenicity (Partida‐Martinez and Hertweck 2005). The identified core endohyphal bacteria likely constitute endosymbiotic partners of *A. fumigatus* . Although mutual dependence is not conclusively established, the inability to cure the host and culture the endohyphal bacteria, along with the observed metabolic enrichment (Figure 3C, Supporting Information 5: Figure S8), supports this possibility. The functional interdependence of the fungal host and its core endohyphal bacterial partners deserves further analysis and validation. This study sets foundational knowledge to start addressing such outstanding questions relying on representative fungal strains and the most abundant bacterial partners within the core bacteriome. To address this, we will analyse how external factors, such as pH and nitrogen availability, modulate the endofungal bacteriome and consequently the partnership virulence, metabolite production and transcriptional signature.

The prevalence of Gram‐negative endohyphal bacteria in the core bacteriome of *A. fumigatus* (Figures 3 and 4) may be due to their ability to utilise the thick‐walled structures of conidia as a temporary, protective habitat, similar to Gram‐negative bacterial residents in chlamydospores (Spraker et al. 2016; Venkatesh et al. 2022). However, the stable bacterial diversity within mycelia, as well as the inability to remove or easily culture them outside of the host, suggests that these Gram‐negative bacteria are true endosymbionts. The fungal diversification timeline dates back to the Jurassic period, with aspergilli established in the Cretaceous period (Steenwyk et al. 2019). It is hypothesised that aspergilli acquired endohyphal bacteria around this time, occurring in parallel to the recruitment of fungi by plants.

## 5 Conclusions

Our study provides important insights into the complex interplay between *A. fumigatus* and its associated endohyphal bacteria, establishing the existence of a potentially clinically relevant core bacteriome. The finding that *Bryobacter* bacteria may increase fungal virulence (Figure 6B) warrants further investigation. Several core endohyphal bacteria, such as *Caulobacter* spp. and *Ralstonia* spp., have been linked to human diseases, including hospital‐acquired meningitis (Manchon et al. 2023; Ryan and Adley 2014). This raises the important question of whether these partnerships enhance the infection capacity of *A. fumigatus* , a species on the WHO's list of fungal priority pathogens (Fisher and Denning 2023). Our results challenge us to shift from a host‐centric vision of fungal–bacterial partnerships to a bacteria‐centric vision: focusing on the roles played by endohyphal bacteria and their interactions and adaptations. Understanding the molecular mechanisms governing the establishment and maintenance of these associations will also require a detailed analysis of host genetic processes, transmission mechanisms and population control.

## Author Contributions

**Daryna Piontkivska:** investigation, writing – original draft, methodology, formal analysis, data curation, writing – review and editing. **João M. P. Jorge:** investigation, methodology, writing – review and editing. **Dalila Mil‐Homens:** investigation, methodology, writing – review and editing. **Tiago M. Martins:** investigation, methodology, formal analysis, writing – review and editing. **Pedro Crespo:** investigation, writing – review and editing. **Demosthenes P. Morales:** investigation, methodology, funding acquisition, writing – review and editing. **Dinah Carvalho:** investigation, writing – review and editing. **José Melo‐Cristino:** investigation, writing – review and editing. **Raquel Sá‐Leão:** writing – review and editing, supervision, funding acquisition. **Gustavo H. Goldman:** funding acquisition, writing – review and editing. **Cristina Silva Pereira:** conceptualization, investigation, funding acquisition, writing – review and editing, supervision.

## Ethics Statement

The authors have nothing to report. Note that all samples—fungal isolates—were provided anonymised, being outside the scope of the General Data Protection Regulation (GDPR 2016/679, national law n° 58/2019). As such, our research and anticipated results are not expected to pose any risks to the rights, freedoms, interests or well‐being of the donors, third parties or the community in general.

## Consent

The authors have nothing to report.

## Conflicts of Interest

The authors declare no conflicts of interest.

## Supporting information


**Supporting Information 1.** Amplicon sequencing data of ITS regions (xls format) of the fungal strains.

**Supporting Information 2.** Microsatellite genotyping data (xls format) of the fungal strains.

**Supporting Information 3.** ASVs sequences, taxa match and abundances and 16S sequences (xls format).

**Supporting Information 4.** Detailed R scripts (MS word).

**Supporting Information 5.** Supporting Information (MS Word), containing the full description of the methods used and a more detailed tables and figures that support the main figure panels in the main text.

**Supporting Information 6.** Spearman’s correlation coefficients (xls format).

## Data Availability

The data that support the findings of this study are openly available in NCBI at https://www.ncbi.nlm.nih.gov/, reference number PRJNA1135973.

## References

[emi470153-bib-0001] Abad, A. , J. M. Fernández‐Molina , J. Bikandi , et al. 2010. “What Makes a Successful Pathogen? Genes and Molecules Involved in Invasive Aspergillosis.” Revista Iberoamericana de Micología 27, no. 4: 155–182. 20974273 10.1016/j.riam.2010.10.003

[emi470153-bib-0002] Almeida, C. , C. Silva Pereira , V. Gonzalez‐Menendez , et al. 2018. “Unveiling Concealed Functions of Endosymbiotic Bacteria Harbored in the Ascomycete Stachylidium Bicolor.” Applied and Environmental Microbiology 84, no. 15: e00660‐18. 29858203 10.1128/AEM.00660-18 PMC6052265

[emi470153-bib-0003] Araldi‐Brondolo, S. J. , J. Spraker , J. P. Shaffer , et al. 2017. “Bacterial Endosymbionts: Master Modulators of Fungal Phenotypes.” Microbiology Spectrum 5, no. 5: funk‐0056‐80. 10.1128/microbiolspec.funk-0056-2016 PMC11687546 28936944

[emi470153-bib-0004] Araujo, D. , D. Mil‐Homens , M. Henriques , and S. Silva . 2022. “Anti‐EFG1 2'‐OMethylRNA Oligomer Inhibits *Candida albicans* Filamentation and Attenuates the Candidiasis in *Galleria mellonella* .” Molecular Therapy—Nucleic Acids 27: 517–523. 35036062 10.1016/j.omtn.2021.12.018 PMC8728520

[emi470153-bib-0005] Blaxter, M. , J. Mann , T. Chapman , et al. 2005. “Defining Operational Taxonomic Units Using DNA Barcode Data.” Philosophical Transactions of the Royal Society of London. Series B, Biological Sciences 360, no. 1462: 1935–1943. 16214751 10.1098/rstb.2005.1725 PMC1609233

[emi470153-bib-0006] Bongomin, F. , S. Gago , R. O. Oladele , and D. W. Denning . 2017. “Global and Multi‐National Prevalence of Fungal Diseases‐Estimate Precision.” Journal of Fungi (Basel, Switzerland) 3, no. 4: 57. 29371573 10.3390/jof3040057 PMC5753159

[emi470153-bib-0007] Callahan, B. J. , P. J. McMurdie , and S. P. Holmes . 2017. “Exact Sequence Variants Should Replace Operational Taxonomic Units in Marker‐Gene Data Analysis.” ISME Journal 11, no. 12: 2639–2643. 28731476 10.1038/ismej.2017.119 PMC5702726

[emi470153-bib-0008] Callahan, B. J. , P. J. McMurdie , M. J. Rosen , A. W. Han , A. J. A. Johnson , and S. P. Holmes . 2016. “DADA2: High‐Resolution Sample Inference From Illumina Amplicon Data.” Nature Methods 13, no. 7: 581–583. 27214047 10.1038/nmeth.3869 PMC4927377

[emi470153-bib-0009] Camuel, A. , A. Teulet , M. Carcagno , et al. 2023. “Widespread Bradyrhizobium Distribution of Diverse Type III Effectors That Trigger Legume Nodulation in the Absence of Nod Factor.” ISME Journal 17, no. 9: 1416–1429. 37355742 10.1038/s41396-023-01458-1 PMC10432411

[emi470153-bib-0010] Caporaso, J. G. , C. L. Lauber , W. A. Walters , et al. 2011. “Global Patterns of 16S rRNA Diversity at a Depth of Millions of Sequences per Sample.” Proceedings of the National Academy of Sciences of the United States of America 108, no. Suppl 1: 4516–4522. 20534432 10.1073/pnas.1000080107 PMC3063599

[emi470153-bib-0011] Chen, W. M. , E. K. James , A. R. Prescott , M. Kierans , and J. I. Sprent . 2003. “Nodulation of Mimosa spp. by the Beta‐Proteobacterium *Ralstonia Taiwanensis* .” Molecular Plant‐Microbe Interactions 16, no. 12: 1051–1061. 14651338 10.1094/MPMI.2003.16.12.1051

[emi470153-bib-0012] Cheng, S. , J. W. Jiang , L. T. Tan , et al. 2022. “Plant Growth‐Promoting Ability of Mycorrhizal Fusarium Strain KB‐3 Enhanced by Its IAA Producing Endohyphal Bacterium, *Klebsiella Aerogenes* .” Frontiers in Microbiology 13: 855399. 35495715 10.3389/fmicb.2022.855399 PMC9051524

[emi470153-bib-0013] Subcommittee on Antifungal Susceptibility Testing of the ESCMID European Committee for Antimicrobial Susceptibility Testing . 2008. “EUCAST Technical Note on the Method for the Determination of Broth Dilution Minimum Inhibitory Concentrations of Antifungal Agents for Conidia‐Forming Moulds.” Clinical Microbiology and Infection 14, no. 10: 982–984. 18828858 10.1111/j.1469-0691.2008.02086.x

[emi470153-bib-0014] de Valk, H. A. , J. F. G. M. Meis , I. M. Curfs , K. Muehlethaler , J. W. Mouton , and C. H. W. Klaassen . 2005. “Use of a Novel Panel of Nine Short Tandem Repeats for Exact and High‐Resolution Fingerprinting of *Aspergillus fumigatus* Isolates.” Journal of Clinical Microbiology 43, no. 8: 4112–4120. 16081958 10.1128/JCM.43.8.4112-4120.2005 PMC1233892

[emi470153-bib-0015] Denning, D. W. 2024. “Global Incidence and Mortality of Severe Fungal Disease.” Lancet Infectious Diseases 24, no. 7: e428–e438. 38224705 10.1016/S1473-3099(23)00692-8

[emi470153-bib-0016] Dial, D. T. , K. M. Weglarz , A. O. Aremu , et al. 2022. “Transitional Genomes and Nutritional Role Reversals Identified for Dual Symbionts of Adelgids (Aphidoidea: Adelgidae).” ISME Journal 16, no. 3: 642–654. 34508228 10.1038/s41396-021-01102-w PMC8857208

[emi470153-bib-0017] Dort, E. N. , E. Layne , N. Feau , et al. 2023. “Large‐Scale Genomic Analyses With Machine Learning Uncover Predictive Patterns Associated With Fungal Phytopathogenic Lifestyles and Traits.” Scientific Reports 13, no. 1: 17203. 37821494 10.1038/s41598-023-44005-w PMC10567782

[emi470153-bib-0018] Dos Santos, R. A. C. , J. L. Steenwyk , O. Rivero‐Menendez , et al. 2020. “Genomic and Phenotypic Heterogeneity of Clinical Isolates of the Human Pathogens *Aspergillus fumigatus* , Aspergillus Lentulus, and Aspergillus Fumigatiaffinis.” Frontiers in Genetics 11: 459. 32477406 10.3389/fgene.2020.00459 PMC7236307

[emi470153-bib-0019] Douglas, G. M. , V. J. Maffei , J. R. Zaneveld , et al. 2020. “PICRUSt2 for Prediction of Metagenome Functions.” Nature Biotechnology 38, no. 6: 685–688. 10.1038/s41587-020-0548-6 PMC7365738 32483366

[emi470153-bib-0020] Dworkin, M. , S. Falkow , E. Rosenberg , K.‐H. Schleifer , and E. Stackebrandt . 2006a. The Prokaryotes: A Handbook on the Biology of Bacteria. Vol. 1. Springer.

[emi470153-bib-0021] Dworkin, M. , S. Falkow , E. Rosenberg , K.‐H. Schleifer , and E. Stackebrandt . 2006b. “Xylophilus.” In The Prokaryotes: A Handbook on the Biology of Bacteria, vol. 6. Springer.

[emi470153-bib-0022] Evans, T. J. , A. Lawal , C. Kosmidis , and D. W. Denning . 2024. “Chronic Pulmonary Aspergillosis: Clinical Presentation and Management.” Seminars in Respiratory and Critical Care Medicine 45, no. 1: 88–101. 38154471 10.1055/s-0043-1776914

[emi470153-bib-0023] Fahimipour, A. K. , and T. Gross . 2020. “Mapping the Bacterial Metabolic Niche Space.” Nature Communications 11, no. 1: 4887. 10.1038/s41467-020-18695-z PMC7522980 32985497

[emi470153-bib-0024] Falagan, C. , and D. B. Johnson . 2014. “Acidibacter Ferrireducens Gen. Nov., sp. Nov.: An Acidophilic Ferric Iron‐Reducing Gammaproteobacterium.” Extremophiles 18, no. 6: 1067–1073. 25116055 10.1007/s00792-014-0684-3

[emi470153-bib-0025] Fang, L. , X. Zheng , Z. Sun , Y. Li , J. Deng , and Y. I. Zhou . 2023. “Characterization of a Plant Growth‐Promoting Endohyphal *Bacillus subtilis* in Fusarium Acuminatum From *Spiranthes sinensis* .” Polish Journal of Microbiology 72, no. 1: 29–37. 36929887 10.33073/pjm-2023-007 PMC10280306

[emi470153-bib-0026] Firacative, C. 2020. “Invasive Fungal Disease in Humans: Are We Aware of the Real Impact?” Memórias do Instituto Oswaldo Cruz 115: e200430. 33053052 10.1590/0074-02760200430 PMC7546207

[emi470153-bib-0027] Fisher, M. C. , and D. W. Denning . 2023. “The WHO Fungal Priority Pathogens List as a Game‐Changer.” Nature Reviews. Microbiology 21, no. 4: 211–212. 10.1038/s41579-023-00861-x PMC9901396 36747091

[emi470153-bib-0028] Fisher, M. C. , S. J. Gurr , C. A. Cuomo , et al. 2020. “Threats Posed by the Fungal Kingdom to Humans, Wildlife, and Agriculture.” mBio 11, no. 3: e00449‐20. 32371596 10.1128/mBio.00449-20 PMC7403777

[emi470153-bib-0029] Hazarika, D. J. , T. Gautom , A. Parveen , et al. 2020. “Mechanism of Interaction of an Endofungal Bacterium *Serratia marcescens* D1 With Its Host and Non‐Host Fungi.” PLoS One 15, no. 4: e0224051. 32320394 10.1371/journal.pone.0224051 PMC7176118

[emi470153-bib-0030] Itabangi, H. , P. C. S. Sephton‐Clark , D. P. Tamayo , et al. 2022. “A Bacterial Endosymbiont of the Fungus *Rhizopus microsporus* Drives Phagocyte Evasion and Opportunistic Virulence.” Current Biology 32, no. 5: 1115–1130. 35134329 10.1016/j.cub.2022.01.028 PMC8926845

[emi470153-bib-0031] Johnson, J. S. , D. J. Spakowicz , B. Y. Hong , et al. 2019. “Evaluation of 16S rRNA Gene Sequencing for Species and Strain‐Level Microbiome Analysis.” Nature Communications 10, no. 1: 5029. 10.1038/s41467-019-13036-1 PMC6834636 31695033

[emi470153-bib-0032] Kamvar, Z. N. , J. F. Tabima , and N. J. Grunwald . 2014. “Poppr: An R Package for Genetic Analysis of Populations With Clonal, Partially Clonal, and/or Sexual Reproduction.” PeerJ 2: e281. 24688859 10.7717/peerj.281 PMC3961149

[emi470153-bib-0033] Kapitan, M. , M. J. Niemiec , A. Steimle , J. S. Frick , and I. D. Jacobsen . 2019. “Fungi as Part of the Microbiota and Interactions With Intestinal Bacteria.” Current Topics in Microbiology and Immunology 422: 265–301. 30062595 10.1007/82_2018_117

[emi470153-bib-0034] Kaur, C. , G. Selvakumar , and A. N. Ganeshamurthy . 2017. “Burkholderia to Paraburkholderia: The Journey of a Plant‐Beneficial‐Environmental Bacterium.” In Recent Advances in Applied Microbiology, edited by P. Shukla , 213–228. Springer.

[emi470153-bib-0035] Kelliher, J. M. , A. J. Robinson , R. Longley , et al. 2023. “The Endohyphal Microbiome: Current Progress and Challenges for Scaling Down Integrative Multi‐Omic Microbiome Research.” Microbiome 11, no. 1: 192. 37626434 10.1186/s40168-023-01634-7 PMC10463477

[emi470153-bib-0036] Kulichevskaya, I. S. , N. E. Suzina , W. Liesack , and S. N. Dedysh . 2010. “ *Bryobacter aggregatus* gen. nov., sp. nov., a Peat‐Inhabiting, Aerobic Chemo‐Organotroph From Subdivision 3 of the Acidobacteria.” International Journal of Systematic and Evolutionary Microbiology 60, no. Pt 2: 301–306. 19651730 10.1099/ijs.0.013250-0

[emi470153-bib-0037] Latge, J. P. , A. Beauvais , and G. Chamilos . 2017. “The Cell Wall of the Human Fungal Pathogen *Aspergillus fumigatus*: Biosynthesis, Organization, Immune Response, and Virulence.” Annual Review of Microbiology 71: 99–116. 10.1146/annurev-micro-030117-020406 28701066

[emi470153-bib-0038] Latge, J. P. , and G. Chamilos . 2019. “ *Aspergillus fumigatus* and Aspergillosis in 2019.” Clinical Microbiology Reviews 33, no. 1: e00140‐18. 31722890 10.1128/CMR.00140-18 PMC6860006

[emi470153-bib-0039] Louca, S. , L. W. Parfrey , and M. Doebeli . 2016. “Decoupling Function and Taxonomy in the Global Ocean Microbiome.” Science 353, no. 6305: 1272–1277. 27634532 10.1126/science.aaf4507

[emi470153-bib-0040] Lv, Y. Y. , Z. H. Gao , F. Xia , M. H. Chen , and L. H. Qiu . 2017. “Puia Dinghuensis Gen. Nov., sp. Nov., Isolated From Monsoon Evergreen Broad‐Leaved Forest Soil.” International Journal of Systematic and Evolutionary Microbiology 67, no. 11: 4639–4645. 28984557 10.1099/ijsem.0.002346

[emi470153-bib-0041] Manchon, R. , V. Zarrouk , V. Leflon , G. Iakovlev , and F. Bert . 2023. “First Case of Bloodstream Infection due to Caulobacter spp. Associated With a Postoperative Meningitis.” IDCases 32: e01761. 37077422 10.1016/j.idcr.2023.e01761 PMC10106900

[emi470153-bib-0042] Martins, C. , D. Piontkivska , D. Mil‐Homens , et al. 2023. “Increased Production of Pathogenic, Airborne Fungal Spores Upon Exposure of a Soil Mycobiota to Chlorinated Aromatic Hydrocarbon Pollutants.” Microbiology Spectrum 11, no. 4: e0066723. 37284774 10.1128/spectrum.00667-23 PMC10434042

[emi470153-bib-0043] McMurdie, P. J. , and S. Holmes . 2013. “Phyloseq: An R Package for Reproducible Interactive Analysis and Graphics of Microbiome Census Data.” PLoS One 8, no. 4: e61217. 23630581 10.1371/journal.pone.0061217 PMC3632530

[emi470153-bib-0044] Meng, A. , C. Marchet , E. Corre , et al. 2018. “A de Novo Approach to Disentangle Partner Identity and Function in Holobiont Systems.” Microbiome 6, no. 1: 105. 29885666 10.1186/s40168-018-0481-9 PMC5994019

[emi470153-bib-0045] Mil‐Homens, D. , S. Barahona , R. N. Moreira , et al. 2018. “Stress Response Protein BolA Influences Fitness and Promotes *Salmonella enterica* Serovar Typhimurium Virulence.” Applied and Environmental Microbiology 84, no. 8: e02850‐17. 29439986 10.1128/AEM.02850-17 PMC5881071

[emi470153-bib-0046] Morales, D. P. , A. J. Robinson , A. C. Pawlowski , et al. 2022. “Advances and Challenges in Fluorescence in Situ Hybridization for Visualizing Fungal Endobacteria.” Frontiers in Microbiology 13: 892227. 35722318 10.3389/fmicb.2022.892227 PMC9199388

[emi470153-bib-0047] Morgulis, A. , G. Coulouris , Y. Raytselis , T. L. Madden , R. Agarwala , and A. A. Schäffer . 2008. “Database Indexing for Production MegaBLAST Searches.” Bioinformatics 24, no. 16: 1757–1764. 18567917 10.1093/bioinformatics/btn322 PMC2696921

[emi470153-bib-0048] Murali, A. , A. Bhargava , and E. S. Wright . 2018. “IDTAXA: A Novel Approach for Accurate Taxonomic Classification of Microbiome Sequences.” Microbiome 6, no. 1: 140. 30092815 10.1186/s40168-018-0521-5 PMC6085705

[emi470153-bib-0049] Nomani, E. , A. Algheethi , N. K. Rahman , B. A. Talip , R. M. S. Radin Mohamed , and M. O. Ab Kadir . 2018. “Single Spore Isolation as a Simple and Efficient Technique to Obtain Fungal Pure Culture.” 4th International Conference on Civil and Environmental Engineering for Sustainability (Iconcees 2017), 140.

[emi470153-bib-0050] Partida‐Martinez, L. P. , and C. Hertweck . 2005. “Pathogenic Fungus Harbours Endosymbiotic Bacteria for Toxin Production.” Nature 437, no. 7060: 884–888. 16208371 10.1038/nature03997

[emi470153-bib-0051] Partida‐Martinez, L. P. , S. Monajembashi , K. O. Greulich , and C. Hertweck . 2007. “Endosymbiont‐Dependent Host Reproduction Maintains Bacterial‐Fungal Mutualism.” Current Biology 17, no. 9: 773–777. 17412585 10.1016/j.cub.2007.03.039

[emi470153-bib-0052] Paulussen, C. , J. E. Hallsworth , S. Álvarez‐Pérez , et al. 2017. “Ecology of Aspergillosis: Insights Into the Pathogenic Potency of *Aspergillus fumigatus* and Some Other Aspergillus Species.” Microbial Biotechnology 10, no. 2: 296–322. 27273822 10.1111/1751-7915.12367 PMC5328810

[emi470153-bib-0053] Pawlowska, T. E. , M. L. Gaspar , O. A. Lastovetsky , et al. 2018. “Biology of Fungi and Their Bacterial Endosymbionts.” Annual Review of Phytopathology 56: 289–309. 10.1146/annurev-phyto-080417-045914 30149793

[emi470153-bib-0054] Quast, C. , E. Pruesse , P. Yilmaz , et al. 2013. “The SILVA Ribosomal RNA Gene Database Project: Improved Data Processing and Web‐Based Tools.” Nucleic Acids Research 41, no. Database issue: D590–D596. 23193283 10.1093/nar/gks1219 PMC3531112

[emi470153-bib-0055] Robinson, A. J. , G. L. House , D. P. Morales , et al. 2021. “Widespread Bacterial Diversity Within the Bacteriome of Fungi.” Communications Biology 4, no. 1: 1168. 34621007 10.1038/s42003-021-02693-y PMC8497576

[emi470153-bib-0056] Rokas, A. 2022. “Evolution of the Human Pathogenic Lifestyle in Fungi.” Nature Microbiology 7, no. 5: 607–619. 10.1038/s41564-022-01112-0 PMC9097544 35508719

[emi470153-bib-0057] Ryan, M. P. , and C. C. Adley . 2014. “Spp.: Emerging Global Opportunistic Pathogens.” European Journal of Clinical Microbiology & Infectious Diseases 33, no. 3: 291–304. 24057141 10.1007/s10096-013-1975-9

[emi470153-bib-0058] Salter, S. J. , M. J. Cox , E. M. Turek , et al. 2014. “Reagent and Laboratory Contamination Can Critically Impact Sequence‐Based Microbiome Analyses.” BMC Biology 12: 87. 25387460 10.1186/s12915-014-0087-z PMC4228153

[emi470153-bib-0059] Schliep, K. P. 2011. “Phangorn: Phylogenetic Analysis in R.” Bioinformatics 27, no. 4: 592–593. 21169378 10.1093/bioinformatics/btq706 PMC3035803

[emi470153-bib-0060] Sewell, T. R. , J. Zhu , J. Rhodes , et al. 2019. “Nonrandom Distribution of Azole Resistance Across the Global Population of *Aspergillus fumigatus* .” mBio 10, no. 3: e00392‐19. 31113894 10.1128/mBio.00392-19 PMC6529631

[emi470153-bib-0061] Shao, M. , C. Sun , X. Liu , et al. 2020. “Upregulation of a Marine Fungal Biosynthetic Gene Cluster by an Endobacterial Symbiont.” Communications Biology 3, no. 1: 527. 32968175 10.1038/s42003-020-01239-y PMC7511336

[emi470153-bib-0062] Sharma, M. , M. Schmid , M. Rothballer , et al. 2008. “Detection and Identification of Bacteria Intimately Associated With Fungi of the Order.” Cellular Microbiology 10, no. 11: 2235–2246. 18637023 10.1111/j.1462-5822.2008.01202.x

[emi470153-bib-0063] Sharmin, D. , Y. Guo , T. Nishizawa , et al. 2018. “Comparative Genomic Insights Into Endofungal Lifestyles of Two Bacterial Endosymbionts, Mycoavidus Cysteinexigens and *Burkholderia rhizoxinica* .” Microbes and Environments 33, no. 1: 66–76. 29540638 10.1264/jsme2.ME17138 PMC5877345

[emi470153-bib-0064] Slater, J. L. , L. Gregson , D. W. Denning , and P. A. Warn . 2011. “Pathogenicity of *Aspergillus fumigatus* Mutants Assessed in *Galleria mellonella* Matches That in Mice.” Medical Mycology 49, no. Suppl 1: S107–S113. 20950221 10.3109/13693786.2010.523852

[emi470153-bib-0065] Spraker, J. E. , L. M. Sanchez , T. M. Lowe , P. C. Dorrestein , and N. P. Keller . 2016. “ *Ralstonia solanacearum* Lipopeptide Induces Chlamydospore Development in Fungi and Facilitates Bacterial Entry Into Fungal Tissues.” ISME Journal 10, no. 9: 2317–2330. 26943626 10.1038/ismej.2016.32 PMC4989320

[emi470153-bib-0066] Steenwyk, J. L. , X. X. Shen , A. L. Lind , G. H. Goldman , and A. Rokas . 2019. “A Robust Phylogenomic Time Tree for Biotechnologically and Medically Important Fungi in the Genera Aspergillus and Penicillium.” mBio 10, no. 4: e00925‐19. 31289177 10.1128/mBio.00925-19 PMC6747717

[emi470153-bib-0067] Steenwyk, J. L. , M. E. Mead , S. L. Knowles , et al. 2020. “Variation Among Biosynthetic Gene Clusters, Secondary Metabolite Profiles, and Cards of Virulence Across Species.” Genetics 216, no. 2: 481–497. 32817009 10.1534/genetics.120.303549 PMC7536862

[emi470153-bib-0068] Tedersoo, L. , M. Albertsen , S. Anslan , and B. Callahan . 2021. “Perspectives and Benefits of High‐Throughput Long‐Read Sequencing in Microbial Ecology.” Applied and Environmental Microbiology 87, no. 17: e0062621. 34132589 10.1128/AEM.00626-21 PMC8357291

[emi470153-bib-0069] Thijs, S. , M. De Op Beeck , B. Beckers , et al. 2017. “Comparative Evaluation of Four Bacteria‐Specific Primer Pairs for 16S rRNA Gene Surveys.” Frontiers in Microbiology 8: 494. 28400755 10.3389/fmicb.2017.00494 PMC5368227

[emi470153-bib-0070] Uehling, J. , A. Gryganskyi , K. Hameed , et al. 2017. “Comparative Genomics of Mortierella Elongata and Its Bacterial Endosymbiont Mycoavidus Cysteinexigens.” Environmental Microbiology 19, no. 8: 2964–2983. 28076891 10.1111/1462-2920.13669

[emi470153-bib-0071] Urbanová, M. , J. Snajdr , and P. Baldrian . 2015. “Composition of Fungal and Bacterial Communities in Forest Litter and Soil Is Largely Determined by Dominant Trees.” Soil Biology and Biochemistry 84: 53–64.

[emi470153-bib-0072] Venkatesh, N. , C. Greco , M. T. Drott , et al. 2022. “Bacterial Hitchhikers Derive Benefits From Fungal Housing.” Current Biology 32, no. 7: 1523–1533.e6. 35235767 10.1016/j.cub.2022.02.017 PMC9009100

[emi470153-bib-0073] Wang, F. , P. Sethiya , X. Hu , et al. 2021. “Transcription in Fungal Conidia Before Dormancy Produces Phenotypically Variable Conidia That Maximize Survival in Different Environments.” Nature Microbiology 6, no. 8: 1066–1081. 10.1038/s41564-021-00922-y 34183813

[emi470153-bib-0074] Wang, S. , A. Meade , H. M. Lam , and H. Luo . 2020. “Evolutionary Timeline and Genomic Plasticity Underlying the Lifestyle Diversity in Rhizobiales.” mSystems 5, no. 4: e00438‐20. 32665328 10.1128/mSystems.00438-20 PMC7363004

[emi470153-bib-0075] Weisburg, W. G. , S. M. Barns , D. A. Pelletier , and D. J. Lane . 1991. “16S Ribosomal DNA Amplification for Phylogenetic Study.” Journal of Bacteriology 173, no. 2: 697–703. 1987160 10.1128/jb.173.2.697-703.1991 PMC207061

[emi470153-bib-0076] Welsh, B. L. , and R. Eisenhofer . 2024. “The Prevalence of Controls in Phyllosphere Microbiome Research: A Methodological Review.” New Phytologist 242, no. 1: 23–29. 38339825 10.1111/nph.19573

[emi470153-bib-0077] West, S. A. , R. M. Fisher , A. Gardner , and E. T. Kiers . 2015. “Major Evolutionary Transitions in Individuality.” Proceedings of the National Academy of Sciences of the United States of America 112, no. 33: 10112–10119. 25964342 10.1073/pnas.1421402112 PMC4547252

[emi470153-bib-0078] Woo, P. C. , P. C. Y. Woo , A. H. Y. Ngan , H.‐K. Chui , S. K. P. Lau , and K.‐Y. Yuen . 2010. “Agar Block Smear Preparation: A Novel Method of Slide Preparation for Preservation of Native Fungal Structures for Microscopic Examination and Long‐Term Storage.” Journal of Clinical Microbiology 48, no. 9: 3053–3061. 20660221 10.1128/JCM.00917-10 PMC2937734

[emi470153-bib-0079] Xu, G. , L. Zhang , X. Liu , et al. 2022. “Combined Assembly of Long and Short Sequencing Reads Improve the Efficiency of Exploring the Soil Metagenome.” BMC Genomics 23, no. 1: 37. 34996356 10.1186/s12864-021-08260-3 PMC8742384

[emi470153-bib-0080] Yarza, P. , P. Yilmaz , E. Pruesse , et al. 2014. “Uniting the Classification of Cultured and Uncultured Bacteria and Archaea Using 16S rRNA Gene Sequences.” Nature Reviews. Microbiology 12, no. 9: 635–645. 25118885 10.1038/nrmicro3330

[emi470153-bib-0081] Zelezniak, A. , S. Andrejev , O. Ponomarova , D. R. Mende , P. Bork , and K. R. Patil . 2015. “Metabolic Dependencies Drive Species Co‐Occurrence in Diverse Microbial Communities.” Proceedings of the National Academy of Sciences of the United States of America 112, no. 20: 6449–6454. 25941371 10.1073/pnas.1421834112 PMC4443341

[emi470153-bib-0082] Zhang, Z. , S. Schwartz , L. Wagner , and W. Miller . 2000. “A Greedy Algorithm for Aligning DNA Sequences.” Journal of Computational Biology 7, no. 1–2: 203–214. 10890397 10.1089/10665270050081478

[emi470153-bib-0083] Zheng, H. , T. Chen , W. Li , J. Hong , J. Xu , and Z. Yu . 2024. “Endosymbiotic Bacteria Within the Nematode‐Trapping Fungus and Their Potential Roles in Nitrogen Cycling.” Frontiers in Microbiology 15: 1349447. 38348183 10.3389/fmicb.2024.1349447 PMC10860758

