# Supplementary material for: Hidden Allies: Decoding the Core Endohyphal Bacteriome of Aspergillus fumigatus
Source: Environ Microbiol Rep. 2025 Aug 19;17(4):e70153. doi: 10.1111/1758-2229.70153 (PMC12365344; doi:10.1111/1758-2229.70153)
Supplement: Supplementary file 4 — Supporting Information 4. Detailed R scripts (MS word). [file EMI4-17-e70153-s006.docx]

**Index:**

**1. #DADA2 PIPELINE FOR ILLUMINA 16S SEQUENCING ANALYSIS**

**2. #DATA ANALYSIS OF AVS TABLE OBTAINED FROM DADA2 PIPELINE**

**3. ####MINION 16S-full length sequencing data analysis**

**4. Version of software used**

**#DADA2 PIPELINE FOR ILLUMINA 16S SEQUENCING ANALYSIS**

#package installation (just need to be done once)

if (!requireNamespace("BiocManager", quietly = TRUE))

BiocManager::install(version = '3.16')

BiocManager::install("dada2")

BiocManager::install("Biostrings")

BiocManager::install("ShortRead")

install.packages("ShortRead")

BiocManager::install("DECIPHER")

BiocManager::install("phyloseq")

install.packages("ggplot2")

library(devtools)

#call installed packages (need to be done always) ans checks the version

library(dada2); packageVersion("dada2")

library(Biostrings); packageVersion("Biostrings")

library(ShortRead); packageVersion("ShortRead")

library(DECIPHER); packageVersion("DECIPHER")

library(phyloseq); packageVersion("phyloseq")

library(ggplot2); packageVersion("ggplot2")

library(MicEco)

(require(stats))

#IMPORTANT NOTES TO USE DADA2

#1. sample are splited into individual per-sample fastq files

#2. primers, adapters, linkers have been removed (done in the script below)

#3. forward and reverse fastq files contain reads in matched order (done in the script bellow)

#Set working directory, is the folder were all files for analysis are stored (is better to create a folder just with seq files)

path = "C:\\Users\\USER\\Documents\\PhD\\SequencingV3V4\\R_analysis\\all_raw"

list.files(path) #important to check if all files are present

#Generate matched lists of the forward and reverse read files

fnFs <- sort(list.files(path, pattern = "_R1_001.fastq", full.names = TRUE))

fnRs <- sort(list.files(path, pattern = "_R2_001.fastq", full.names = TRUE))

#Extract samples names

sample.names <- sapply(strsplit(basename(fnFs), "-N"), `[`, 1)

#Inspect the quality profiles of the reads

plotQualityProfile(fnFs[1:6]) #forward

plotQualityProfile(fnRs[1:6]) #reverse

##"pre-filter" the sequences

#remove Ns (ambiguous bases), remove primers

fnFs.pre_filt <- file.path(path, "pre_filt", paste0(sample.names, "_F_prefilt.fastq")) # Put filtered files in pre_filtered subdirectory

fnRs.pre_filt <- file.path(path, "pre_filt", paste0(sample.names, "_R_prefilt.fastq"))

filterAndTrim(fnFs, fnFs.pre_filt, maxN = 0, multithread = FALSE, trimLeft = 19, trimRight=20)

filterAndTrim(fnRs, fnRs.pre_filt, maxN = 0, multithread = FALSE, trimLeft = 19, trimRight=20)

##Filter and Trim

# Place filtered files in filtered/ subdirectory

filtFs <- file.path(path, "filtered", paste0(sample.names, "_F_filt.fastq"))

filtRs <- file.path(path, "filtered", paste0(sample.names, "_R_filt.fastq"))

names(filtFs) <- sample.names

names(filtRs) <- sample.names

#maxEE parameter sets the maximum number of "expected errors" allowed in a read (may need to be changed depending on the samples)

#truncLen parameter trims end of the red to n bp

out <- filterAndTrim(fnFs.pre_filt, filtFs, fnRs.pre_filt, filtRs, truncLen=c(240,200),

maxN=0, maxEE=c(2,2), truncQ=2, rm.phix=TRUE,

compress=TRUE, multithread=FALSE, matchIDs=TRUE)

head(out)

**#DATA ANALYSIS OF AVS TABLE OBTAINED FROM DADA2 PIPELINE**

###

library(Biostrings); packageVersion("Biostrings") #BiocManager::install("Biostrings")

library(ShortRead); packageVersion("ShortRead") #BiocManager::install("ShortRead")

library(DECIPHER); packageVersion("DECIPHER") #BiocManager::install("DECIPHER")

library(phyloseq); packageVersion("phyloseq") #BiocManager::install("phyloseq")

library(ggplot2); packageVersion("ggplot2")

library(msa); packageVersion("msa") #BiocManager::install("msa")

library(phangorn); packageVersion("phangorn")

library(ggtree); packageVersion("ggtree") #BiocManager::install("ggtree")

library(scales); packageVersion("scales")

library(ape)

library(tidytree)

library(aplot)

library(tidyverse)

library(Hmisc)

library(tidyr)

library(tibble)

library(ggcorrplot)

library(mia) #BiocManager::install("mia")

library(scater)

library(ggsignif) #plot significance level to plot

library(gridExtra)

library(ggtreeExtra)

library(microbiome)

###

set.seed(10)

#Importing dataframes

tax_table <- read.csv("C:\\Users\\USER\\Documents\\PhD\\SequencingV3V4\\R_analysis\\all_raw\\CSV treated\\tax_table.csv", header=TRUE, row.names="ID", sep =";")

refseq <- read.csv("C:\\Users\\USER\\Documents\\PhD\\SequencingV3V4\\R_analysis\\all_raw\\CSV treated\\refseq.csv", header=TRUE, row.names="ASV", sep =";")

abund_table <- read.csv("C:\\Users\\USER\\Documents\\PhD\\SequencingV3V4\\R_analysis\\all_raw\\CSV treated\\abund_table.csv", header=TRUE, row.names="ID", sep =";")

sam_data <- read.csv("C:\\Users\\USER\\Documents\\PhD\\SequencingV3V4\\R_analysis\\all_raw\\CSV treated\\sam_data.csv", header=TRUE, row.names="ID", sep =";")

#abund_table_rev <- read.csv("C:\\Users\\USER\\Documents\\PhD\\SequencingV3V4\\R_analysis\\all_raw\\CSV treated\\abund_table_rev.csv", header=TRUE, row.names="ASV", sep =";")

#Sequences information

dna <- Biostrings::DNAStringSet(refseq$Sequence)

names(dna) <- row.names(refseq)

#Construction of phylogenetic tree using phangron package

mult <- msa(dna, method="ClustalW", type="dna", order="input") #alignment of sequences

phang.align <- as.phyDat(mult, type="DNA")

#Model test

mt <- modelTest(phang.align, model=c("JC", "F81", "K80", "HKY", "SYM", "GTR"),

control = pml.control(trace = 0))

#infer a phylogenetic tree with best found model

fitK80 <- pml_bb(phang.align, model="K80+G(4)+I")

plot(fitK80)

#mid-point-rooted maximum likelihood phylogenetic tree

midpoint_fitK80 = midpoint(fitK80$tree)

plot(midpoint_fitK80)

add.scale.bar()

#Creating matrix with all information to use in phyloseq

asv_info.relativ <- phyloseq(otu_table(as.matrix(abund_table), taxa_are_rows = FALSE),

sample_data(sam_data),

tax_table(as.matrix(tax_table)),

phy_tree(midpoint_fitK80))

asv_info.relativ <- merge_phyloseq(asv_info, dna)

asv_info.relativ

#Save dataframe as RData

save(asv_info.relativ, file="16S_antibio_taxid_table_dada2_tree_v3v4_all_final.RData")

#load("~/PhD/Sequencing_analysis_22/Mycelium_ciprofloxacin_treatment/Data/16S_antibio_taxid_table_dada2_tree_final.RData")

#Convert abundances to relative abundances

#asv_info.relativ <- transform_sample_counts(asv_info, function(OTU) OTU/sum(OTU)*100)

###################################################

###Look to all ASVs

##Heatmap at class level total ASVs

#phyloseq object need to be summed at class level

genus_pobject <- tax_glom(asv_info.relativ, taxrank="genus")

genus_df <- psmelt(genus_pobject) #transform to dataframe

#plot heatmap

heatmap_class <- ggplot(genus_df, aes(x = Sample, y=genus, fill = Abundance)) +

geom_tile(color = "#283747",lwd = 0.1,linetype = 1) +

scale_fill_gradientn(name = "Relative Abundance (%)",

colours= c("#FDFEFE", "#7FB3D5", "#A9DFBF", "#F9E79F"),

na.value = "transparent",

values = c(0, rescale(10, from = range(class_df$Abundance)), 1))+

theme(strip.background=element_rect(fill="#ebebeb"),

strip.text.x = element_text(size = 11, face = "bold"),

legend.text = element_text(size = 12),

legend.title = element_text(size = 13, face = "bold", vjust = 1.8),

legend.key.size = unit(0.8, "cm"),

axis.text.x = element_text(size =11, color = "black", angle = 90),

axis.text.y = element_text(size= 11, color = "black"),

axis.title.y = element_blank(),

axis.title.x = element_text(size=13))+

coord_fixed() #makes tiles in square shape

heatmap_class

#save with height:650

##Correlation coefficients between the samples (using dataframe: abund_table_rev)

corr_pearson <- rcorr(as.matrix(abund_table_rev)) #dataframe needs to be transformed to matrix

corr_matrix <- corr_pearson$r #correlation matrix

p_value <- corr_pearson$P #p-value matrix

corrplot(corr_matrix, type = "lower", order = "hclust",diag=FALSE, method= "color",

p.mat = p_value, sig.level = c(0.001, 0.01, 0.05), insig = "label_sig")

corr_pearson_plot <- ggcorrplot(corr_matrix, hc.order = TRUE, type = "lower",

outline.col = "white", legend.title = "Pearson Correlation",

ggtheme = ggplot2::theme_gray,

colors = c("#6D9EC1", "white", "#D98880"),

p.mat = p_value) +

theme(legend.text = element_text(size = 10),

legend.title = element_text(size = 12, face = "bold", vjust = 2))

corr_pearson_plot

#save with height:472

##Alpha and Beta diversities

#To use mia package the phyloseq object need to me converted

asv_tse <- makeTreeSummarizedExperimentFromPhyloseq(asv_info.relativ) #convert phyloseq to TSE

asv_tse

#Calculate observed richness ()

asv_tse <- mia::estimateRichness(asv_tse, abund_values = "counts", index = "observed", name="observed")

#calculate diversity:

asv_tse <- mia::estimateDiversity(asv_tse , abund_values = "counts",index = "shannon", name = "shannon")

asv_tse <- mia::estimateDominance(asv_tse , abund_values = "counts", index="relative")

#Calculate eveness

asv_tse <- mia::estimateEvenness(asv_tse , abund_values = "counts", index="pielou")

head(colData(asv_tse)) #check dataframe

plot_shannon <- plotColData(asv_tse, "shannon", "Antibiotic", colour_by = "Isolate") +

ylab(expression("Shannon Index")) +

theme_gray()

plot_evenness <- plotColData(asv_tse, "pielou", "Antibiotic", colour_by = "Isolate") +

theme_gray() +

ylab(expression("Evenness Index"))

plot_relative <- plotColData(asv_tse, "relative", "Antibiotic", colour_by = "Isolate") +

theme_gray() +

ylab(expression("Dominance Index"))

aplha_div_plot <- grid.arrange(plot_shannon, plot_evenness, plot_relative, ncol=3)

aplha_div_plot

#Checking p-values using non-parametric Kolmogorov-Smirnov test for two-group comparisons

#Create data frame from the collected data

colData_df <- as.data.frame(colData(asv_tse))

# Split the values by group for each calculated index

spl_pielou <- split(df$pielou, df$Antibiotic) #evenness index

spl_shannon <- split(df$shannon, df$Antibiotic) #diversity index

spl_relative <- split(df$relative, df$Antibiotic) #dominance index

# Kolmogorov-Smironv test comparing the two groups: Antibiotic - yes and now

pvalue_pielou <- ks.test(spl_pielou$no, spl_pielou$yes)$p.value

pvalue_shannon <- ks.test(spl_shannon$no, spl_shannon$yes)$p.value

pvalue_relative <- ks.test(spl_relative$no, spl_relative$yes)$p.value

pvalue_list <- c(pvalue_pielou,pvalue_shannon,pvalue_relative)

# Adjust the p-value using Benjamini & Hochberg method

padj <- p.adjust(pvalue_list, method ="BH")

padj

Index <- c("evenness", "diversity", "domimance")

Signif <- c("NS", "NS", "NS")

pvalue_df <- data.frame(Index, pvalue_list, Signif)

###################################################

###Look to 100 most abundant ASVs

top100 <- names(sort(taxa_sums(asv_info.relativ), decreasing=TRUE))[1:100]

asv.top100 <- prune_taxa(top100, asv_info.relativ)

##Creating tree plot

#First step is to know witch node correspond to witch class, to define later in dataframe

tree_nodes = ggtree(asv_info.relativ, ladderize = FALSE, size = 1, aes(color=family)) +

#geom_point(aes(color = class),na.rm=TRUE, size = 2) +

geom_text2(aes(subset=!isTip, label=label), hjust=-2, size=0.8) +

geom_treescale(fontsize=3, linesize=0.8, offset=-2) +

geom_text(aes(label=node), hjust=-.3, size =2) +

geom_tiplab(align =TRUE, size = 2) +

geom_nodelab(aes(label=label), hjust=-.05, size=0.1)

print(tree_nodes)

#Define the dataframe with the class nodes to hilight

nodes_top100 <- c(172,174,134,185,102,121,184,149,90,74,181,34)

class_node_top100 <- c("Acidobacteriae", "Actinobacteria", "Alphaproteobacteria","Bacilli",

"Bacteroidia", "Clostridia", "Cyanobacteriia", "Gammaproteobacteria",

"Oligoflexia", "Polyangia", "Vampirivibrionia", "Verrucomicrobiae")

nodes_data_top100 <- data.frame(node=nodes_top100, type=class_node_top100)

#Retrieve just tree info from phyloseq object

tree_info_top100 = asv.top100@phy_tree

#To plot the tree with the the heatmap, we need to transform phyloseq object to dataframe (selection columns of interest)

top100_df <- psmelt(asv.top100) %>% select(label = OTU, Abundance, Colony, Isolate)

#plot the tree with highlighted class

tree_top100_class <- ggtree(tree_info_top100, ladderize = FALSE, size = 0.8) +

geom_text2(aes(subset=!isTip, label=label), hjust=1.5, size=2, vjust = 1) +

#geom_text(aes(label=node), hjust=-.3, size =2) +

#geom_treescale(fontsize=3, linesize=1, offset=-5) +

geom_tiplab(align =TRUE, size =2) +

geom_hilight(data=nodes_data_top100, aes(node=node, fill=type)) +

hexpand(.01)+

theme(legend.text = element_text(size =13),

legend.title = element_text(size = 14),

legend.key.size = unit(0.7, "cm")) +

guides(fill=guide_legend(title="Class"))

tree_top100_class

##Creating heatmap of relative abundance

heatmap_top100 <- ggplot(top100_df, aes(x=Antibiotic, y=label, fill = Abundance)) +

geom_tile(color = "#283747",lwd = 0.1,linetype = 1) + facet_wrap(~Isolate) +

theme(axis.text.y=element_blank(), axis.title.y = element_blank()) +

scale_fill_gradientn(name = "Relative Abundance (%)",

colours= c("#FDFEFE","#7FB3D5", "#2471A3", "#A9DFBF", "#F9E79F", "#FAD7A0"),

na.value = "transparent",breaks=c(0,5,10,15,20,25),

values = c(0, rescale(6, from = range(top100_df$Abundance)), 1)) + #makes color scale discontinuous

theme(strip.background=element_rect(fill="#ebebeb"),

strip.text.x = element_text(size = 11, face = "bold"),

legend.text = element_text(size = 12),

legend.title = element_text(size = 13, face = "bold", vjust = 1.5),

legend.key.size = unit(0.8, "cm"),

legend.direction="horizontal",

axis.text.x = element_text(size =11, color = "black"),

axis.title.x = element_text(size=13)) +

coord_fixed(ratio = 0.25)

heatmap_top100

#Combine the two plots

heatmap_top100 %>% insert_left(tree_top100_class)

#100 top bacteria correspond to >90% of relative abundance

top100_df %>% group_by(Isolate, Colony) %>% summarise(abundance = sum(Abundance))

#################

##Beta-diversity

#UniFrac metric incorporates phylogenic information into beta-diversity analysis

# PCoA plot using the unweighted UniFrac as distance

uwunifrac_dist = phyloseq::distance(asv_info.relativ, method="unifrac", weighted=F)

un.ordination = ordinate(asv_info.relativ, method="PCoA", distance=uwunifrac_dist)

#plot

pcoa_uw <- plot_ordination(asv_info.relativ, un.ordination) + geom_point(size = 2.8) +

xlim(-0.5,0.5) + ylim(-0.5, 0.5) + coord_fixed(ratio = 1) +

scale_color_manual(values = c("#7FB3D5", "#F1948A")) +

theme(axis.text.x = element_text(size =10, color = "black"),

axis.text.y = element_text(size =10, color = "black"),

legend.text = element_text(size = 11),

legend.title = element_text(size = 12, face = "bold", vjust = 1.5))

#PERMANOVA

permanova_uw = adonis2(uwunifrac_dist, data = metadf)

permanova_uw$aov.tab

# PCoA plot using the weighted UniFrac as distance

wunifrac_dist = phyloseq::distance(asv_info.relativ, method="wunifrac")

w.ordination = ordinate(asv_info.relativ, method="PCoA", distance=wunifrac_dist)

#plot

pcoa_w <- plot_ordination(asv_info.relativ, w.ordination) + geom_point(size = 2.8) +

xlim(-0.5,0.5) + ylim(-0.5, 0.5) + coord_fixed(ratio = 1) +

scale_color_manual(values = c("#7FB3D5", "#F1948A")) +

theme(axis.text.x = element_text(size =10, color = "black"),

axis.text.y = element_text(size =10, color = "black"),

legend.text = element_text(size = 11),

legend.title = element_text(size = 12, face = "bold", vjust = 1.5))

pcoa_w

#PERMANOVA

permanova_w = adonis(wunifrac_dist ~ Antibiotic, data = metadf)

permanova_w$aov.tab

cowplot::plot_grid(pcoa_w, pcoa_uw, nrow = 1, ncol = 2, scale = .9, labels = c("Weighted", "Unweighted"))

#Generate distances

ord_unifrac <- ordinate(asv_info.relativ, method = "PCoA",

distance = "wunifrac", grouping_column = 'Antibiotic')

ord_unifrac_un <- ordinate(asv_info.relativ, method = "PCoA", distance = "unifrac")

pcoa_uw <- plot_ordination(asv_info.relativ, ord_unifrac_un, color = "Antibiotic") + geom_point(size = 2.8) +

xlim(-0.5,0.5) + ylim(-0.5, 0.5) + coord_fixed(ratio = 1) +

scale_color_manual(values = c("#7FB3D5", "#F1948A")) +

theme(axis.text.x = element_text(size =10, color = "black"),

axis.text.y = element_text(size =10, color = "black"),

legend.text = element_text(size = 11),

legend.title = element_text(size = 12, face = "bold", vjust = 1.5))

cowplot::plot_grid(pcoa_w, pcoa_uw, nrow = 1, ncol = 2, scale = .9, labels = c("Weighted", "Unweighted"))

metadf <- data.frame(sample_data(asv_info.relativ))

unifrac.dist <- UniFrac(asv_info.relativ,

weighted = TRUE,

normalized = FALSE,

parallel = FALSE,

fast = TRUE)

unifrac.dist

permanova <- adonis(unifrac.dist ~ Antibiotic, data = metadf)

permanova

anosim <- anosim(unifrac.dist, grouping =)

**####MINION 16S-full length sequencing data analysis**

###

library(Biostrings); packageVersion("Biostrings")

library(ShortRead); packageVersion("ShortRead")

library(DECIPHER); packageVersion("DECIPHER")

library(phyloseq); packageVersion("phyloseq")

library(ggplot2); packageVersion("ggplot2")

library(msa); packageVersion("msa")

library(phangorn); packageVersion("phangorn")

library(ggtree); packageVersion("ggtree")

library(scales); packageVersion("scales")

library(ape)

library(tidytree)

library(aplot)

library(tidyverse)

library(Hmisc)

library(tidyr)

library(tibble)

library(ggcorrplot)

library(mia)

library(scater)

library(ggsignif) #plot significance level to plot

library(gridExtra)

library(RColorBrewer)

library(microbiome)

library(vegan)

library(dendsort)

library(ggtreeExtra)

library(ggnewscale)

###

#Load phyloseq object

load("D:\\USERMinION\\New_Analysis\\all_phyloseq.RData")

genus <- unique(data.frame(all_info@tax_table)$genus)

genus_bact <- genus[!genus %in% c("Ralstonia", "Methylobacterium-Methylorubrum","Burkholderia-Caballeronia-Paraburkholderia")]

#Separete into main groups

#Ralstonia, Methylobcter, Brukholderia

all_ralstonia <- subset_taxa(all_info, genus == "Ralstonia")

#phylogenetic agglomeration

h<- 0.5 #define the desired

agg_ralstonia <- tip_glom(all_ralstonia, h = h)

all_mm <- subset_taxa(all_info, genus == "Methylobacterium-Methylorubrum")

#phylogenetic agglomeration

h<- 0.3 #define the desired

agg_mm <- tip_glom(all_mm, h = h)

all_bcp <- subset_taxa(all_info, genus == "Burkholderia-Caballeronia-Paraburkholderia")

#phylogenetic agglomeration

h<- 0.25 #define the desired

agg_bcp <- tip_glom(all_bcp, h = h)

#mixed bacteria genus

other_mix <- c("Novosphingobium", "Shewanella","Serratia","Pantoea",

"Bacillus", "Facklamia", "Planomicrobium", "Aquabacterium",

"Pelomonas","Delftia","Comamonas")

all_other_mix <- subset_taxa(all_info, genus %in% other_mix)

h <- 0.13

agg_other_mix <- tip_glom(all_other_mix, h = h)

#other not mixed

other <- genus_bact[!genus_bact %in% other_mix]

all_other <- subset_taxa(all_info, genus %in% other)

h <- 0.2

agg_other <- tip_glom(all_other, h = h)

########Join all agg#######

#create datafarmes

tax_table_01 <- data.frame(agg_ralstonia@tax_table)

abund_table_01 <- data.frame(agg_ralstonia@otu_table)

seq_agg_01 <- data.frame(agg_ralstonia@refseq)

colnames(seq_agg_01)[colnames(seq_agg_01) == "agg_ralstonia.refseq"] <- "Seq"

tax_table_02 <- data.frame(agg_bcp@tax_table)

abund_table_02 <- data.frame(agg_bcp@otu_table)

seq_agg_02 <- data.frame(agg_bcp@refseq)

colnames(seq_agg_02)[colnames(seq_agg_02) == "agg_bcp.refseq"] <- "Seq"

tax_table_03 <- data.frame(agg_mm@tax_table)

abund_table_03 <- data.frame(agg_mm@otu_table)

seq_agg_03 <- data.frame(agg_mm@refseq)

colnames(seq_agg_03)[colnames(seq_agg_03) == "agg_mm.refseq"] <- "Seq"

tax_table_04 <- data.frame(agg_other_mix@tax_table)

abund_table_04 <- data.frame(agg_other_mix@otu_table)

seq_agg_04 <- data.frame(agg_other_mix@refseq)

colnames(seq_agg_04)[colnames(seq_agg_04) == "agg_other_mix.refseq"] <- "Seq"

tax_table_05 <- data.frame(agg_other@tax_table)

abund_table_05 <- data.frame(agg_other@otu_table)

seq_agg_05 <- data.frame(agg_other@refseq)

colnames(seq_agg_05)[colnames(seq_agg_05) == "agg_other.refseq"] <- "Seq"

#join tables

tax_table_all <- rbind(tax_table_03, tax_table_04, tax_table_05, tax_table_01,tax_table_02)

seq_all <- rbind(seq_agg_03, seq_agg_04, seq_agg_05, seq_agg_01, seq_agg_02)

abund_table_all <- bind_rows(abund_table_03, abund_table_04, abund_table_05, abund_table_01,

abund_table_02)

abund_table_all[is.na(abund_table_all)] <- 0

#Creating phyloseq object for all

#Sequences information

dna <- Biostrings::DNAStringSet(seq_all$Seq)

names(dna) <- row.names(seq_all)

all_info_agg <- phyloseq(otu_table(as.matrix(abund_table_all), taxa_are_rows = TRUE),

tax_table(as.matrix(tax_table_all)),

refseq(dna))

all_info_agg <- merge_phyloseq(all_info_agg, dna)

all_info_agg

#Construction of phylogenetic tree using phangron package

mults_all <- msa(all_info_agg@refseq, method="ClustalW", type="dna", order="input") #alignment of sequences

phang.align <- as.phyDat(mults_all, type="DNA")

#Model test

#mt <- modelTest(phang.align, model=c("JC", "F81", "K80", "HKY", "SYM", "GTR"),

#control = pml.control(trace = 0))

#infer a phylogenetic tree with best found model

fitGTRGI_all <- pml_bb(phang.align, model="GTR+G(4)+I")

plot(fitGTRGI_all)

#mid-point-rooted maximum likelihood phylogenetic tree

midpoint_fitGTRGI_all = midpoint(fitGTRGI_all$tree)

plot(midpoint_fitGTRGI_all)

add.scale.bar()

#Adding tree info to phyloseq object

all_info_agg <- merge_phyloseq(all_info_agg, phy_tree(midpoint_fitGTRGI_all))

#add sample data

sam_data <- read.csv("D:\\USERMinION\\New_Analysis\\sam_data.csv", header=TRUE, row.names="Code", sep =";")

sample_data(all_info_agg) <- sam_data

#Save

save(all_info_agg, file="D:\\USERMinION\\New_Analysis\\all_agg_phyloseq.RData")

load("D:\\USERMinION\\New_Analysis\\all_agg_phyloseq.RData")

#Clean ASV table names

tax_table(all_info_agg)[, colnames(tax_table(all_info_agg))] <- gsub(tax_table(all_info_agg)[, colnames(tax_table(all_info_agg))],

pattern = "Methylobacterium-Methylorubrum", replacement = "MM")

tax_table(all_info_agg)[, colnames(tax_table(all_info_agg))] <- gsub(tax_table(all_info_agg)[, colnames(tax_table(all_info_agg))],

pattern = "Burkholderia-Caballeronia-Paraburkholderia", replacement = "BCP")

#########Data analysis#######

#Core heatmap

#Use the format_to_besthit function from microbiomeutilities to get the best classification of the ASVs

all_genus <- aggregate_taxa(all_info_agg, "genus")

all_genus <- subset_taxa(all_genus, genus!="Unknown")

# Core with compositionals:

prevalences <- c(0, 1)

detections = c(0.1, 0.5, 1, 5)

p.core <- plot_core(all_genus,

plot.type = "heatmap",

colours = rev(brewer.pal(8, "Spectral")),

prevalences = prevalences,

detections = detections,

min.prevalence = 0.75) +

xlab("Detection Threshold

(Relative Abundance (%))")

#asthetics

p.core + theme(axis.text.y = element_text(size=10, color = 'black'),

axis.text.x = element_text(size = 11, color = 'black', angle = 90, hjust=1,vjust=0.3),

axis.title.y = element_text(size=10, face = 'bold'),

legend.text = element_text(size =10, colour = 'black'),

legend.title = element_text(size = 11, colour = 'black', face = 'bold')) +

scale_fill_gradientn(name = "Prevalence", colors = rev(brewer.pal(8, "Spectral")),

breaks=c(0,0.2,0.4,0.6,0.8,1)) +

coord_flip() +

scale_y_discrete(position = 'left')

#Tree with abundances

##Getting info to add

all_agg_df <- psmelt(all_info_agg)

# Filter out rows where Abundance = 0

all_agg_df <- all_agg_df %>% filter(Abundance != 0)

##Dataframe for log10 abundance

all_abund_genus_df <- select(all_agg_df, OTU, Abundance, Name)

all_abund_genus_df <- all_abund_genus_df %>% mutate(Abundance = log10(Abundance)) #log10 abundance transformation

all_abund_genus_df <- distinct(all_abund_genus_df , .keep_all = TRUE) #remove duplicates

all_abund_genus_df <- all_abund_genus_df %>% pivot_wider(names_from = Name, values_from = Abundance)

all_abund_genus_df <- data.frame(all_abund_genus_df, row.names = "OTU")

##Dataframe for genus

all_agg_genus_df <- select(all_agg_df, OTU, genus)

all_agg_genus_df <- distinct(all_agg_genus_df , .keep_all = TRUE) #remove duplicates

all_agg_genus_df <- data.frame(all_agg_genus_df, row.names = "OTU")

all_tree = ggtree(all_info_agg, ladderize = FALSE, size = 0.8, layout = "circular") +

geom_tiplab(aes(label=genus), align =TRUE, size =3) +

geom_treescale(fontsize=3, linesize=1, offset=0.4)

#all_tree <- rotate_tree(all_tree, -90)

print(all_tree)

abund_tree = gheatmap(all_tree, all_abund_genus_df, offset=0, width=.4,

colnames_angle=0, colnames_offset_y = 0) +

theme(legend.text = element_text(size = 10, color = 'black'),

legend.key.size = unit(0.6, "cm"))+

scale_fill_gradientn(colours = c("#EBF5FB", "#7FB3D5","#A9DFBF", "#F9E79F", "#F5CBA7", "#EB984E"),

na.value = "white", name = "Relative Abundance (log10)")

abund_tree

all_genus_abund <- abund_tree + new_scale_fill()

colors = c("#B2ABD2","#35978F","#9970AB","#BABABA","#BF812D","#F7F7F7","#053061",

"#1B7837","#D53E4F","#74ADD1","#878787","#A50026","#FDDBC7",

"#E6F5D0","#E08214","#7F3B08","#2D004B","#01665E","#F1B6DA",

"#FEE08B","#00441B","#F46D43","#4393C3","#FEE0B6","#DE77AE",

"#C2A5CF","#313695","#D8DAEB","#FEE08B","#A6DBA0", "#F7F7a4",

"#D73027","#67001F","#543039","#80CDC1","#543008","#C7EAE5",

"#FDAE61","#1A9850","#762A83", "#D9F0D3", "#87CEEB", "#FF6347",

"#B0E0E6", "#FFA07A", "#C1FFC1", "#D8BFD8", "#FFEC8B", "#98FB98","#ADD8E6")

all_genus = gheatmap(all_genus_abund, all_agg_genus_df, offset=-1, width=0.95,

colnames_angle=0, colnames_offset_y = 0) +

theme(legend.text = element_text(size = 9, color = 'black'),

legend.key.size = unit(0.4, "cm")) +

scale_fill_manual(values = colors) +

guides(fill = guide_legend(ncol = 2))

all_genus

all_genus = gheatmap(all_tree, all_agg_genus_df, offset=-0.05, width=.05,

colnames_angle=0, colnames_offset_y = 0) +

theme(legend.text = element_text(size = 9, color = 'black'),

legend.key.size = unit(0.4, "cm")) +

scale_fill_manual(values = colors) +

guides(fill = guide_legend(ncol = 2))

all_genus

all_genus_abund <- all_genus + new_scale_fill()

gheatmap(all_genus_abund, all_abund_genus_df, offset=0.01, width=.4,

colnames_angle=0, colnames_offset_y = 0) +

theme(legend.text = element_text(size = 10, color = 'black'),

legend.key.size = unit(0.6, "cm"))+

scale_fill_gradientn(colours = c("#EBF5FB", "#7FB3D5","#A9DFBF", "#F9E79F", "#F5CBA7", "#EB984E"),

na.value = "white", name = "Relative Abundance (log10)")

#Presence/absence heatmap

pa_genus_df <- select(all_agg_df, Name, genus)

pa_genus_df <- distinct(pa_genus_df , .keep_all = TRUE) #remove duplicates

# Use dplyr to create a new data frame with counts

counts <- pa_genus_df %>%

group_by(genus) %>%

summarise(count = n_distinct(Name))

counts_pa_genus_df <-merge(pa_genus_df, counts, by = "genus", all= T)

#dataframe for distance

counts_pa_genus_df = counts_pa_genus_df[, c("Name", "count", "genus")] %>%

pivot_wider(names_from = genus, values_from = count)

counts_pa_genus_df[is.na(counts_pa_genus_df)] <- 0

counts_pa_genus_df = data.frame(counts_pa_genus_df , row.names = "Name")

#The plot

pheatmap(counts_pa_genus_df, na_col = "transparent",

color = c("transparent", "#EBF5FB", "#7FB3D5","#7FB3D5","#7FB3D5","#7FB3D5","#7FB3D5","#7FB3D5","#7FB3D5", "#154360"),

fontsize=10.5, border_color= "white", cluster_rows=FALSE)

colors = c("#B2ABD2","#9970AB","#35978F","#67001F","#BF812D","#D9F0D3","#053061",

"#1B7837","#D53E4F","#74ADD1","#878787","#A50026","#FDDBC7",

"#F1B6DA","#FEE08B","#FDAE61","#2D004B","#01665E","#D8DAEB")

melt_simple <- psmelt(core_z) %>%

select(OTU, val=Abundance)

tree_log10 = genus_core + geom_fruit(data=melt_simple, geom=geom_boxplot,

mapping = aes(y=OTU, x=val, group=label,fill=genus),

pwidth = 0.8, size=.2, outlier.size=0.8, outlier.stroke=0.1, offset = 0.4,

outlier.shape=21, axis.params=list(axis = "log10", text.size = 2,

hjust = 1,vjust = 0.5, nbreak = 2,),

grid.params=list())

core_tree_log10 <- tree_log10 +

scale_fill_manual(values = colors) +

theme(legend.position = "none")

core_tree_log10

#Heat map with correlation

#group to genus level

all_genus <- tax_glom(all_info_agg, taxrank="genus", NArm = TRUE)

#Transform data to log10 score

all_log10 <- transform(all_genus, 'log10')

#Transform data to a dataframe

all_log10_df <- psmelt(all_log10) #transform to dataframe

all_log10_df_na = replace(all_log10_df, all_log10_df == 0, NA)

#dataframe for distance

all_df_adundance_na = all_log10_df_na[, c("Name", "Abundance", "genus")] %>%

pivot_wider(names_from = genus, values_from = Abundance)

all_df_adundance_na = data.frame(all_df_adundance_na , row.names = "Name")

#dataframe for plot

all_log10_df_adundance = all_log10_df[, c("Name", "Abundance", "genus")] %>%

pivot_wider(names_from = genus, values_from = Abundance)

all_log10_df_adundance = data.frame(all_log10_df_adundance , row.names = "Name")

#Hierarchical clustering using Bray-Curtis distances

cluster_samples <- hclust(vegdist(all_log10_df_adundance, method="bray", na.rm = TRUE))

sort_hclust <- function(...) as.hclust(dendsort(as.dendrogram(...)))

cluster_sapmles <- sort_hclust(cluster_samples)

plot(cluster_sapmles, main = "Sorted Dendrogram", xlab = "", sub = "")

#check number of possible clusters by heaigth

clusters = cutree(cluster_sapmles, k = NULL, h = 0.3)

max(clusters)

#Get the isolates virulance and resistance data

isolates_data <- sam_data[, (ncol(sam_data) - 4):ncol(sam_data)]

isolates_data <- data.frame(isolates_data, row.names = "Name")

#Define the colors

my_colour <- list(Infection.capacity = c("High" = "#4A235A", "Medium-high" = "#7D3C98",

"Medium-low" = "#BB8FCE", "Low" = "#EBDEF0"),

Amphotericin.B = c("Resistant" = "#C0392B", "Susceptible" = "#16A085"),

Voriconazole = c("Resistant" = "#C0392B", "Susceptible" = "#16A085"),

Posaconazole = c("Resistant" = "#C0392B", "Susceptible" = "#16A085"))

#The plot

pheatmap(all_df_adundance_na, na_col = "transparent",

cluster_cols=FALSE, cluster_rows = cluster_sapmles,

annotation_row = isolates_data,

color = colorRampPalette(c("#EBF5FB", "#7FB3D5", "#A9DFBF", "#F9E79F", "#F5CBA7", "#EB984E"))(100),

annotation_colors = my_colour,

fontsize=10.5, border_color= "white",

cutree_rows = 3)

ggtree(all_info_agg, ladderize = FALSE, size = 0.8, open.angle=10) +

geom_text2(aes(subset=!isTip, label=label), hjust=1.5, size=2, vjust = 1) +

#geom_text(aes(label=node), hjust=-.3, size =2) +

geom_treescale(fontsize=3, linesize=1, offset=0.2) +

geom_tiplab(aes(label=genus), align =TRUE, size =3.5)

plot_bar(all_info_agg, fill="genus") +

geom_bar(aes(color=genus), stat="identity", position="stack")

bar_absolut

any(is.na(data.frame(agg_all@tax_table)$genus))

**Versions of software used**

package ape version 5.7.1

package tidytree version 0.4.2

package aplot version 0.1.9

package tidyverse version 1.3.2

package Hmisc version 5.1.0

package tidyr version 1.3.0

package tibble version 3.2.1

package ggcorrplot version 0.1.4

package mia version 1.6.0

package scater version 1.26.1

package ggsignif version 0.6.4

package gridExtra version 2.3

package ggtreeExtra version 1.8.1

package microbiome version 1.20.0

package ShortRead version 1.56.1

package DECIPHER version 2.26.0

package dada2 version 1.26.0

package phyloseq version 1.42.0

package ggplot2 version 3.4.0

package msa version 1.30.1

package phangorn version 2.11.1

package ggtree version 3.6.2

package scales version 1.2.1
