# Supplementary material for: Hidden Allies: Decoding the Core Endohyphal Bacteriome of Aspergillus fumigatus
Source: Environ Microbiol Rep. 2025 Aug 19;17(4):e70153. doi: 10.1111/1758-2229.70153 (PMC12365344; doi:10.1111/1758-2229.70153)
Supplement: Supplementary file 5 — Supporting Information 5. Supporting Information (MS Word), containing the full description of the methods used and a more detailed tables and figures that support the main figure panels in the main text. [file EMI4-17-e70153-s001.docx]

**Hidden allies: Decoding the core endohyphal bacteriome of *Aspergillus fumigatus***

Daryna Piontkivska^1^, João M.P. Jorge^1#^, Dalila Mil-Homens^2,3#^, Tiago M. Martins^1#^, Pedro Crespo^1^, Demosthenes P. Morales^4^, Dinah Carvalho^5^, José Melo-Cristino^5^, Raquel Sá-Leão^1^, Gustavo H. Goldman^1,6,7^, Cristina Silva Pereira^1^*

^1^Instituto de Tecnologia Química e Biológica António Xavier, Universidade Nova de Lisboa (ITQB NOVA), Oeiras, Portugal

^2^Institute for Bioengineering and Biosciences (iBB) and Institute for Health and Bioeconomy (i4HB), Instituto Superior Técnico, University of Lisbon, Lisboa, Portugal

^3^Department of Bioengineering, Instituto Superior Técnico, University of Lisbon, Lisboa, Portugal

^4^Center of Integrated Nanotechnologies, Los Alamos National Laboratory, Los Alamos, NM, 87545, USA

^5^Laboratory of Microbiology, Unidade Local de Saúde Santa Maria, Lisboa, Portugal

^6^Faculdade de Ciências Farmacêuticas de Ribeirão Preto, Universidade de São Paulo, Brazil

^7^National Institute of Science and Technology in Human Pathogenic Fungi, São Paulo, Brazil

^#^equal contributing authors

*corresponding author: Cristina Silva Pereira (spereira@itqb.unl.pt)

Supporting Information **5:** Supporting Information (MS Word), containing the full description of the Methods used and more detailed tables and figures that support the main figures panels at the main text:

**Figure S1.** Bacteria found in negative controls amplification and extraction.

**Figure S2.** A, Venn diagram displaying ASVs overlaps and B, Pearson’s correlation coefficients between fungal bacteriome at genus level before and after antibiotic pressure.

**Figure S3.** Maximum likelihood midpoint rooted tree of the 100 most abundant bacterial ASVs across the sample set.

**Figure S4.** A, Minimum spanning network of Aspergillus fumigatus isolates genotyped at nine microsatellite loci; B, Discriminant analysis of principal components and C, its density plot.

**Table S1.** Minimal inhibitory concentrations of each tested antifungal drug against each *Aspergillus fumigatus* strains.

**Figure S5.** Infection capacity of each A. fumigatus clinical isolate and one soil isolate (Af_SI.00) using Galleria mellonella as infection model.

**Table S2.** Ratio and derived percentages of 18S/16S rDNA in spores and mycelium.

**Figure S6.** Stacked bar chart showing the relative abundance ASVs from the bacterial V3-V4 hypervariable region of 16S rRNA sequences, taxonomically classified at genus level.

**Figure S7.** Phylogenetic tree of the core bacteriome.

**Figure S8.** Prediction of the ecologically relevant functions of the *Aspergillus fumigatus* isolates bacteriome.

**Figure S9**. Metagenome analysis of whole genome sequencing reads from two clinical isolates.

**Figure S10.** (A) Shannon diversity, richness, and evenness of the fungal bacteriome in different infection capacity groups; (B) Hierarchical clustering heat map of fungal bacteriome using Bray-Curtis distance, without core bacteriome.

**Material and Methods**

***Culture media.*** *Aspergillus* Minimal Medium (AMM) containing glucose (10 g·L^-1^), thiamine (0,01 g·L^-1^), 5% (v/v) nitrate salts solution [NaNO_3_ (120 g·L-1), KCl (10.4 g·L^-1^), MgSO_4_·7H_2_O (10.4 g·L^-1^) and KH_2_PO_4_ (30.4 g·L^-1^)], 0.1% (v/v) trace element solution [ZnSO4·7H_2_O (22.0 g·L^-1^), H_3_BO_4_ (11.0 g·L^-1^), MnCl_2_·4H_2_O (5.0 g·L^-1^), FeSO_4_·7H_2_O (5.0 g·L^-1^), CoCl_2_·6H_2_O (1.7 g·L^-1^), CuSO4·5H_2_O (1.6 g·L^-1^), Na_2_MoO_4_·2H_2_O (1.5 g·L^-1^) and Na_4_EDTA (50 g·L^-1^)], the final pH was adjusted to 6.5 with NaOH. For solid media, 1.5% (w/v) of agar was added.

***Clinical strain collection***

*Aspergillus* strains were isolated by the Laboratory of Microbiology of Hospital Santa Maria (HSM), Lisbon, Portugal, between August and October of 2018 (Table 1, Supporting Information 1). The fungal isolates were obtained from sputum specimens or from bronchoalveolar lavage. Samples were inoculated on to Sabouraud Dextrose Agar (SDA) plates containing chloramphenicol and gentamicin (SGA, Biomérieux) and incubated at 35-37 °C for 7 to 10 days. Fungal isolates were collected during routine sampling of patients, and were provided anonymised, without any information that could be used to identify the patient. All fungal isolates were identified phenotypically, at the species level, based on macroscopic colony morphology and micromorphological characteristics. Additionally, fungal isolates were subjected to molecular identification by amplification and sequencing of ITS as previously reported ([Martins, Piontkivska et al. 2023](#_ENREF_12)). For each fungal strain, conidia stocks were prepared and stored at -80 °C until required. In some assays, *A. fumigatus* reference laboratory strain Af293, purchased from the Fungal Genetics Stock Center (Kansas City, MO, United States), and a soil isolate AEM006 ([Martins, Piontkivska et al. 2023](#_ENREF_12)), referred as Af_SI.00, were used.

***Microsatellite genotyping.*** Microsatellite genotyping was used to determine the genetic distances between the *Aspergillus fumigatus* clinical isolates. Genotyping was performed by CD Genomics (NY, USA) with a panel of nine short tandem repeats (TRs) as previously described ([de Valk, Meis et al. 2005](#_ENREF_6)). In brief, three separate multiplex PCRs amplifications were performed to obtain three dinucleotide, three trinucleotide and three tetranucleotide loci fragments. Each PCR mixture contained 1x reaction buffer, 0.3 mM of corresponding amplification primers, 0.2 mM deoxynucleotide triphosphates, 0.5 U of Hot Start Taq DNA Polymerase and 10 ng of genomic DNA. Amplification was performed using the following thermal protocol: 5 min of initial denaturation at 95 °C, followed by 35 cycles of 30s of denaturation at 95 °C, 30s of annealing at 60 °C and 30s of extension at 72 °C, with final extension for 30 min at 60 °C. The fragments obtained were denatured at 95 °C for 3 min in a mixture containing 1.0 μl of PCR product, molecular weight internal standard and 0.05 % formamide. The fragment analysis was performed on the Applied Biosystems 3730xl DNA Analyzer, and the sizes determined using GeneMapper Software 5. The data is shown in Supporting Information 2.

The minimum spanning network, to determine genetic diversity between strains, was determined using Bruvo’s distance, and the discriminant analysis of principal components (DAPC) was performed using the R package poppr (v2.9.5) ([Kamvar, Tabima et al. 2014](#_ENREF_8)). Data obtained in this study was compared with a subset of isolates from the databank for clinical and environmental *A. fumigatus* strains, which is based at the Canisius Wilhelmina Hospital in Nijmegen, Netherlands, and available at the afumID website ([Sewell, Zhu et al. 2019](#_ENREF_20)).

***Aspergillus single-conidium cultures***

To minimize inter-spore variability and eliminate transient bacteria, a single-spore culture technique was applied ([Nomani, Al-Gheethi et al. 2018](#_ENREF_16)). Additionally, spores underwent a high-temperature treatment. In brief, spore suspensions in 0.85% NaCl solution (100 conidia·mL^-1^) were incubated at 60 °C for 1h to kill most bacteria associated with the spores’ surface. Two bacterial controls were used: *Escherichia coli* and *Hydrobacter penzbergensis*. After the heat-treatment, no bacterial colonies were visible after 24h at 37 °C on Luria-Bertani (LB) agar and Reasoner's 2A(R2A) agar plates. Following heat-treatment, aliquots (100 µl) of the conidia were spread on solidified AMM supplemented with 100 mg·L^-1^ of ciprofloxacin (37 °C, 16-20h). One germinated spore was selected, carefully cut, and transferred to fresh medium (supplemented with 100 mg·L^-1^ of ciprofloxacin) and further incubated at 37 °C. Conidia were harvested after 5-7 days of growth using a saline solution (NaCl 8.5 g⋅L^-1^) containing 0.01 % Tween^®^ 20, washed two times with saline solution and collected after passing through three layers of miracloth. The resulting spore suspensions were conserved in saline solution containing 30% (v/v) glycerol at -80 °C until further use.

***Cultures conditions.*** Fungal biomass was obtained by growing ~10^8^ spores·mL^‑1^ in 4 mL of liquid AMM, supplemented with or without 100 mg·L^‑1^ of ciprofloxacin, in 6-well plates for 48h. Each fungal strain was grown on a separate 6-well plate. Grown mycelia were removed from the medium surface, immediately frozen, and stored at ‑80 °C until DNA extraction.

***DNA extraction.*** The mycelium biomass was ground in liquid nitrogen using mortar-pestle, followed by performing 1 cycle of heating (1 h at 90 °C) and sonication (each tube contained 1 g of glass beads per sample, with equal amounts of 0.5- and 0.1-mm beads) for 5 min, with the aid of an extraction buffer (50 mM NaH_2_PO4, 50 mM NaCl, 500 mM Tris-HCl, 5% SDS, pH 8; 1 mL per culture). Afterwards, the sample was mixed with an equal volume 25:24:1 (v) mixture of phenol, chloroform and isoamyl alcohol (solution A), shaken (2 min) and centrifuged (5 min, 2,400*g*) to recover the aqueous phase which was re-extracted with an equal volume 24:1 (v) mixture of chloroform and isoamyl alcohol (solution B) and recovered as described before. To the recovered aqueous phase, 20 µL of RNAse (10 mg·ml^-1^) were added and incubated at 37 ºC for 30 min. The mixture was re-extracted again with solution B as described before. To the recovered aqueous phase, 1/3 volume of 6M NaCl and 1/10 volume of 10% of cetyltrimethylammonium bromide (CTAB) in 0.7M NaCl were added, and the mixture was incubated for 30 min at 65 °C. After cooling to room temperature, an equal volume of solution B was added, shaken, and centrifuged (20 min, 1,400*g*) to recover the supernatant. Finally, DNA was precipitated in 2/3 volume of isopropanol and 1/10 volume of sodium acetate solution (3M) overnight at 4 °C. The precipitated DNA was recovered by centrifugation (20 min, 6,800*g*). The DNA pellet was washed with 200 µL of ethanol (75%), recovered by centrifugation as before, air dried and then eluted in 50-200 µL of TE buffer (Qiagen, Germany) and finally stored at -20 °C. When needed, DNA concentration was measured using a Nanodrop OneC (ThermoFisher, USA).

***Amplification of the V4 or V3-V4 regions of 16S rRNA gene.*** Bacteriome profiling relied on amplicon sequencing of the V4 and/or V3-V4 regions of the 16S rRNA gene using DNA extracted from 2-day old mycelium. Nested PCR was used to enhance the bacterial signal: the PCR product of the first amplification with universal primer set fD1/rP2 ([Weisburg, Barns et al. 1991](#_ENREF_23)) was used as the template for a second amplification with the nested primer set 515F/806R ([Caporaso, Lauber et al. 2011](#_ENREF_4)) to amplify the V4 region or 341F/785R ([Thijs, Op De Beeck et al. 2017](#_ENREF_22)) to amplify the V3-V4 region of the 16S rRNA gene. The PCR mixture for the remaining amplifications contained 2.5 μL of 10x DreamTaq buffer (includes 20 mM MgCl_2_), 0.5 μL of 50 mM MgCl2, 0.75 μL of 10 mM dNTPs, 1.5 μL of 0.15 mM BSA, 0.75 μL of DreamTaq DNA Polymerase (5 U·μL^− 1^, Thermo Scientific™), 1 μL of 5 μM forward primer, 1 μL of 5 μM reverse primer and 50-400 ng of DNA template, adjusted to a final volume of 25 μL with ultrapure water. PCR amplifications were performed using a T100™ Thermal Cycler (Bio-Rad). The amplification using the 16S rRNA gene universal primers fD1 5’‑AGAGTTTGATCCTGGCTCAG-3’; rP2 5’‑ACGGCTACCTTGTTACGACTT-3’, was performed under the following conditions: 5 min at 96 °C, 10 cycles of 30s at 94 °C, 45s annealing at 62-52 °C (decrements of 1 °C per cycle) and 90s at 72 °C, followed by 20 cycles of 30s at 94 °C, 45 s annealing at 52 °C and 90s at 72 °C, with a final extension step at 72 °C for 5 min. For the primers 515F (5′-GTGYCAGCMGCCGCGGTAA-3′) and 806R (5′-GGACTACNVGGGTWTCTAAT-3′) the conditions used were: 5 min at 96 °C, 10 cycles of 30s at 94 °C, 45s annealing at 60-50 °C (decrements of 1 °C per cycle) and 90s at 72 °C, followed by 20 cycles of 30s at 94 °C, 45s annealing at 50 °C and 90s at 72 °C, with a final extension step at 72 °C for 5 min. The PCR products were analysed by agarose-gel electrophoresis. The “no-template” controls (amplification and extraction) were always handled simultaneously, and in a manner consistent with the DNA samples derived from mycelia. Additionally, all work was conducted according to strict sterile techniques (e.g., clean workspaces, barrier tips, pre- and post-PCR pipettes) to limit potential cross-contamination. Before further analyses, all PCR products were purified using GeneClean® Turbo Kit (MP Biomedicals™). All samples (region V4 or V3-V4) were sequenced on Illumina MiSeq (2 × 300 bp paired-end reads) using the Illumina 16S Metagenomic Sequencing service of STAB Vida Lda., Portugal.

***Analysis of the V4 or V3-V4 regions of 16S rRNA gene amplicons.*** Amplicon reads were processed using the divisive amplicon denoising algorithm DADA2 (v1.26.0) ([Callahan, McMurdie et al. 2016](#_ENREF_3)) to infer ASVs present in each sample ([Callahan, McMurdie et al. 2017](#_ENREF_2)) Default settings were used for filtering and trimming. Built-in training models were utilized to learn error rates for the amplicon dataset. Identical sequencing reads were combined through DADA2’s dereplication functionality, and the DADA2 sequence–variant inference algorithm was applied to each dataset. Subsequently, paired-end reads were merged. Taxonomy assignment was performed using the DECIPHER (v2.26.0) package with the IDTAXA algorithm ([Murali, Bhargava et al. 2018](#_ENREF_15)) that employs principles from machine learning to reduce over classification errors. The closest bacterial hit from the SILVA SSU database r138 ([Quast, Pruesse et al. 2013](#_ENREF_18)) was considered (Supporting Information 3), and sequences identified as nonbacterial were discarded. All bacterial genera represented by ASVs identified in DNA amplification or extraction controls were excluded from further analysis.

The ASVs were imported directly in the R software (v4.3.2, R Core Team, 2023). The phylogenetic trees were constructed in R using the phangorn package (v2.11.1), with sequence alignment generated by the msa package (v1.30.1) using the ClustalW method ([Schliep 2011](#_ENREF_19)). Model testing was performed using the modelTest() function to evaluate a set of nucleotide substitution models, including JC, F81, K80, HKY, SYM, and GTR, assessing their suitability based on the alignment. To suppress verbose output, the control option pml.control(trace = 0) was used (<https://cran.r-project.org/web/packages/phangorn/phangorn.pdf>). The general time reversible model with the rate variation among sites described by a gamma distribution and a proportion of invariable sites (GTR + G + I) was identified as the best-fitting model for our dataset. Phylogenetic trees were inferred using the pml_bb() function, which applies the maximum likelihood method to estimate tree topology and branch lengths. The resulting tree was visualized using the plot() function. To assess the reliability of bootstrap replicates, and the bootstrap values were mapped onto the tree. The corresponding R scripts are provided in Supporting Information 4. Inkscape software 1.4 was used to correct typos in the names of bacteria classes.

***Full-length 16S rRNA gene sequencing on the MinION™ platform.*** For the library construction, 100-200 ng of genomic DNA was used and processed with the 16S barcoding kit (SQK-RAB204, ONT, Oxford, United Kingdom), following to the manufacturer’s instruction with a few modifications. No-template controls (amplification and extraction) were always handled simultaneously and in a manner consistent with the fungal samples. The obtained bar-coded library was sequenced in a FLO-MIN106 flow cell using the MinION Mk1C device (ONT). MinKNOW v21.05.25 (ONT) and Guppy v5.0.16 were used for data acquisition. A total of 2.79 M reads were generated.

***Analysis of full-length 16S rRNA gene.*** From the amplicon reads obtained, only those that contained both forward and reverse primers were used for further processing (in total 22,666 reads). To reduce redundancy and to increase the efficiency of downstream analyses, reads were clustered into OTUs based on phylogeny-derived distances, using the *tip_glom* function in the *phyloseq* R (v1.42.0) package ([McMurdie and Holmes 2013](#_ENREF_13)) (tree-height threshold for similarity: h = 0.2). The OTUs were taxonomically identified against the SILVA SSU database r138 ([Quast, Pruesse et al. 2013](#_ENREF_18)) using the *blastn* tool, the closest bacterial hit was considered, and sequences identified as nonbacterial were discarded. All bacterial OTUs identified in DNA amplification or extraction controls were excluded from further analysis (Supporting Information 5: Figure S1). The sequences were imported directly in the R software (v4.3.2, R Core Team, 2023), and the phylogenetic trees were constructed using the general methodology described above. The R scripts are detailed in the Supporting Information 4.

***In vivo* infection with *Galleria mellonella.*** Infection studies were performed as previously described using *G. mellonella* ([Martins, Piontkivska et al. 2023](#_ENREF_12)). Briefly, *G. mellonella* larvae were reared in darkness at 25°C, from egg to last instar larvae, on a natural diet of beeswax and pollen grains. For the experiments, final instar larvae weighing 200 ± 25 mg were selected (n=30 per condition). Aliquots of the stock spore suspensions (derived from single-conidium cultures, see above) were used to adjust the inoculum to a concentration of 10^7^ spores per larva for each tested condition. A microinjection system was used to control the volume of a microsyringe and inject 5 μL of spore suspension into each larva via the hindmost left proleg, previously sanitized with 70% (v/v) ethanol. Following injection, larvae were placed in Petri dishes and stored in the dark at 37 °C. Control larvae were injected with saline solution (pH 7.4). For each condition (n=30) larval survival was followed for 96 h. Caterpillars were considered dead when they displayed no movement in response to touch.

**Minimal Inhibitory Concentrations (MIC) of antifungals.** The minimal inhibitory concentrations (MIC) were determined using a microbroth dilution format according to the EUCAST reference method ([Subcommittee on Antifungal Susceptibility Testing of the 2008](#_ENREF_21)). Tests were performed using RPMI-1640 medium (R6504, with L-glutamine and without sodium bicarbonate) supplemented with glucose to a final concentration of 2% and 0.165 mol·L^-1^ of 3-(N-morpholino) propanesulfonic acid (MOPS) with pH adjusted to 7.0. The concentrations of antifungal drugs tested ranged from 16 to 0.03 mg·L^-1^ for amphotericin B and posaconazole, and from 8 to 0.016 mg·L^-1^ for voriconazole. Spore suspensions were prepared to a final working concentration of 10^6^ conidia·mL^-1^. Growth and negative controls were included in all tests. The microplates were incubated at 37 °C for 48 h. The lowest concentration that exhibited no growth under microscopic observation was considered to be the MIC.

**Microscopy.** *Fluorescence microscopy*. Hydrogel media discs were prepared for use in fluorescence microscopy. Discs were prepared using AMM followed by the addition of Phytagel to a final concentration of 4% w/v. The hydrogel slide preparation was adapted from methods described previously ([Woo, Ngan et al. 2010](#_ENREF_24), [Morales, Robinson et al. 2022](#_ENREF_14)). Briefly, the media was autoclaved and kept in a water bath at 90 ºC. Afterwards the media was cast between two standard microscope slides (VWR, previously washed with ethanol and UV sterilized) and set to solidify under sterile conditions in a biosafety cabinet. The slides were then carefully separated using a sterile razor blade. Using a sterile 1 mL pipette tip, circular discs (~7 mm diameter) were stamped out. These discs were transferred to a 6-well plate, inoculated with 1 µL of a suspension of ~10^9^ spores·mL^‑1^, and incubated at 37 °C. To avoid drying of the discs during the incubation period, UV sterilized paper towels saturated with sterile water were folded and placed in the space between the wells. After 20 h of incubation, the discs were fixed with 4% paraformaldehyde in PBS and incubated overnight at 4 °C. The fixed discs were washed three times with 1x PBS and stored in a solution of PBS with 0.1 U/µL Superase RNAse inhibitor with 10 mM Ribonucleoside Vanadyl Complex (RVC) at 4 °C until needed. Before use, the discs were washed three times with 1x PBS, dehydrated with ethanol-PBS solutions with 50 %, 75 %, 100 %, 75 %, 50 % ethanol, rinsed with PBS for 3 min at room temperature, and after removal of PBS were left to air dry for 10 min. For staining, each sample was first covered with 2 µM syto9 (Invitrogen, 5 min, room temperature, dark) and after washing with PBS, samples were covered with 20 µM calcofluor-white (Sigma, 3 min, room temperature, dark), washed again with PBS, then transferred to a microscope slide with a cover slip on the top of the disc. Some samples were stained with 5 µg·mL^-1^ Hoechst 33342 (Thermo Fischer Scientific, 5 min, room temperature, dark) before the calcofluor-white staining. Mycelia and bacteria were visualized using a Zeiss LSM 880 microscope equipped with a Fast Airyscan. ImageJ was used for image analysis. *Fluorescence in situ hybridization (FISH) microscopy*. The universal 16S rRNA probe EUB338: 5′-GCTGCCTCCCGTAGGAGT-3′ with a Cy3 dye flanking both the 5’ and 3’ ends, and the 18S probe EUK516: 5’-ACCAGA CTTGCCCTCC-3’ with ATTO 647 on the 5’ end, were purchased from IDT. Fungal and bacterial cell walls of fungal discs were first digested with a mixture of 50 µg·mL^‑1^chitinase, 0.5 mg·mL^-1^ glucanase and 1 mg·mL^-1^ lysozyme (1h, 37 °C). The discs were rinsed twice with 1x PBS and then pre-treated for hybridization in a solution of 6x SSC + 15 % formaldehyde for 20 min at 37 °C. A probe solution was prepared as described: 125 nM of each hybridization probe (16S and 18S rRNA targeting probes) was added to a 6x SSC + 15 % formamide solution containing 0.1 mg·mL^-1^ UltraPure Salmon Sperm DNA Solution, 0.5 mg·mL^-1^ UltraPure BSA, 20 mM ribonucleoside vanadyl complex, and 0.1 U/µL SUPERase•In RNase Inhibitor. Staining was performed at 37 °C for 4 h and then rinsed twice with 6x SSC + 15 % formaldehyde (20 min, 37 °C). Finally, the discs were stained with a 1 µg·mL^‑1^solution of DAPI in 6x SSC (5 min, room temperature). After washing twice with 2x SSC, the discs were mounted on a number 1.5 cover glass and microscope slide with ProLong Glass mounting solution, and stored away from light for at least 24 h prior to imaging. FISH staining was visualized on an Olympus IX83 microscope and images were analysed and prepared using ImageJ.

*Transmission electron microscopy (TEM).* Mycelia (SDA medium, 36 h, 37 ºC) were fixed with a solution of 2% (v/v) formaldehyde (Science Services), 2.5% glutaraldehyde (v/v) (Science Services) in 0.1 M cacodylate buffer (30 min, room temperature), then washed three times with 0.1 M cacodylate buffer. Fungal samples were sandwiched between 0.2 µm flat aluminum disks and frozen in a high-pressure freezer (Wohlwend Compact 2) using as filler 0.5% (w/v) low-melting point agarose (OmniPur) in 0.1 M cacodylate buffer at 37 ºC. These samples were placed in the freeze substitution device (Leica AFS2) at -90 ºC in a solution with 2% (w/v) osmium tetroxide (Science Services), and 0.1% (w/v) uranyl acetate (Analar) with 1% distilled H_2_O in acetone. After 60 h, the samples were slowly warmed at a rate of 2 ºC/h: first to -60 ºC, kept at this temperature for 10 h, then to -30ºC and kept at this temperature for 10 h. Subsequently, using the maximum warming rate, samples were brought to 0 ºC and dehydrated with acetone (3 times, 10 min each). Dehydrated samples were infiltrated with increasing amounts of Embed-812 Epoxy Resin (Science Services) (5% 4 h, 10% overnight, 25% 4 h, 50% overnight, 75% 4 h, 100% overnight) and polymerized in fresh resin at 60 ºC for 48 h. Ultrathin sections (70 nm) were cut using an ultramicrotome (Leica UC7), picked-up in 1% formvar (Agar Scientific) in chloroform coated slot grids, and stained at room temperature with 1% (w/v) uranyl acetate and Reynolds lead citrate, for 5 min each. The grids were observed using a TEM (FEI Tecnai G2 Spirit BioTWIN with an Olympus-SIS Veleta CCD Camera) at 120 kV.

**Whole-genome sequencing.** The quality and quantity of DNA were accessed by electrophoresis and using the Qubit dsDNA BR kit, following AMpureXP cleaning. For 150 bp pair-end DNA sequencing, libraries were generated using the Kapa HyperPrep kit (Roche) according to the manufacturer’s instructions and integrity accessed using a TapeStation 4200. Samples were indexed and sequenced on the lllumina Novaseq platform (≥30 million reads per sample). Quality control of reads was carried out using FastQC (v0.12.1) and trimmomatic (v0.39) ([Bolger, Lohse et al. 2014](#_ENREF_1)). The surviving reads (>99%) from each library were aligned to a library of three *A. fumigatus* reference genomes (for strains Af293, A1163 and CEA10, respectively ASM265v1, ASM15014v1 and CP097563 to CP097570) using segemehl (v0.3.4) ([Otto, Stadler et al. 2014](#_ENREF_17)). Metagenomic analysis of the remaining reads (≤2%) was conducted using Kraken (v2.1.3) with PlusPF Refseq indexes as of June 5, 2023 ([Lu, Rincon et al. 2022](#_ENREF_10)) and Recentrifuge (v1.12.1) ([Marti 2019](#_ENREF_11)). Reads were also aligned to specific bacterial genomes as described above, and genome coverage determined using Samtools (v1.17) ([Danecek, Bonfield et al. 2021](#_ENREF_5)).

**Microbiome function prediction.** FAPROTAX (v1.2.10) and PICRUSt2 (v2.5.2) were used to predict the ecologically relevant functions of the microbiomes from the fungal isolates ([Louca, Parfrey et al. 2016](#_ENREF_9), [Douglas, Maffei et al. 2020](#_ENREF_7)). Both analyses employed default parameters using relative abundance ASVs from the bacterial V3-V4 hypervariable region of the 16S rRNA sequences. For simplicity in the presentation of the PICRUSt2 prediction, MetaCyc pathways were categorized into class pathways.

**
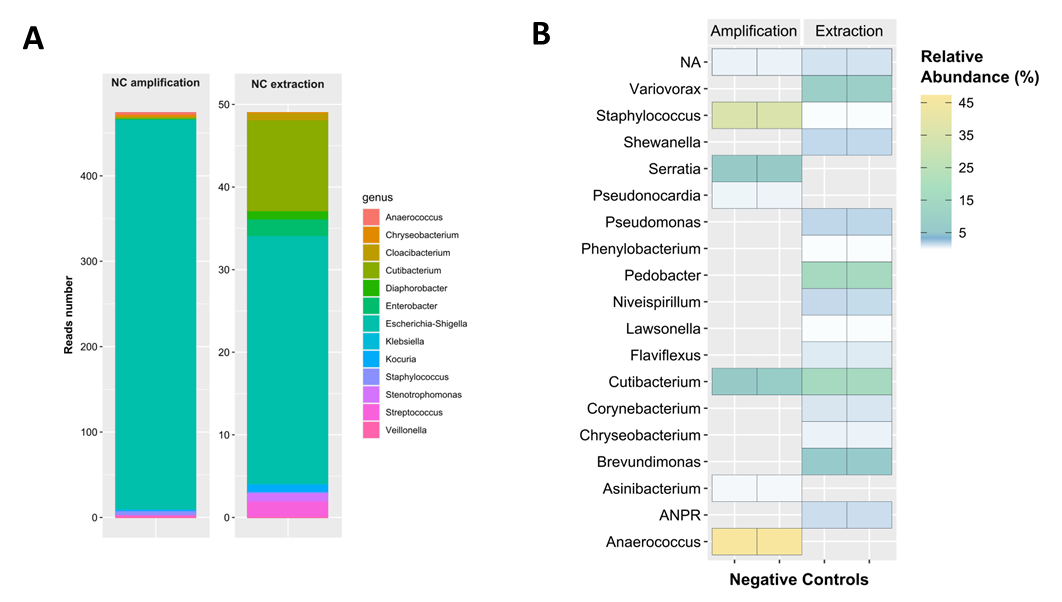
**

**Figure S1.** (A) Bacteria found in negative controls amplification and extraction. All bacterial genera identified in negative control samples were excluded from further analysis (B) Genus-level profile of contaminant ASVs found in negative controls of PCR amplification and DNA extraction, performed in 2 separate days. All bacterial ASVs present in negative control samples were excluded from further analysis. ANPR stands *for Allorhizobium-Neorhizobium-Pararhizobium-Rhizobium* group.

**
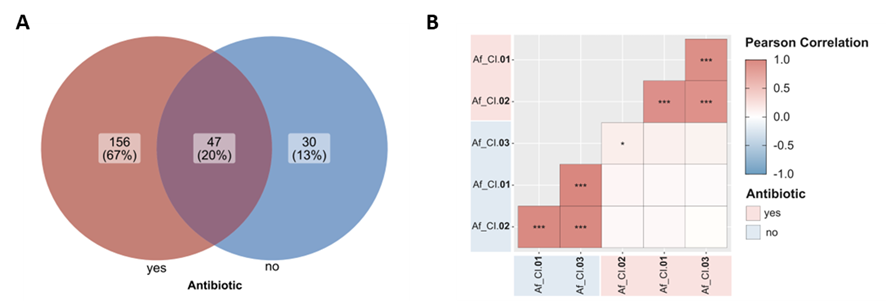
**

**Figure S2.** (A) Venn diagram displaying ASVs overlaps and (B) Pearson’s correlation coefficients between fungal bacteriome at genus level before (no) and after (yes) antibiotic pressure. Note that the 47 common ASVs were found at relative high abundances in mycelia cultivated in media both without and with antibiotic selection, representing 70-78% and 69-75% of the total relative abundance, respectively. Asterisks indicate significant correlations *P ≤ 0.01; ***P ≤ 0.0001.


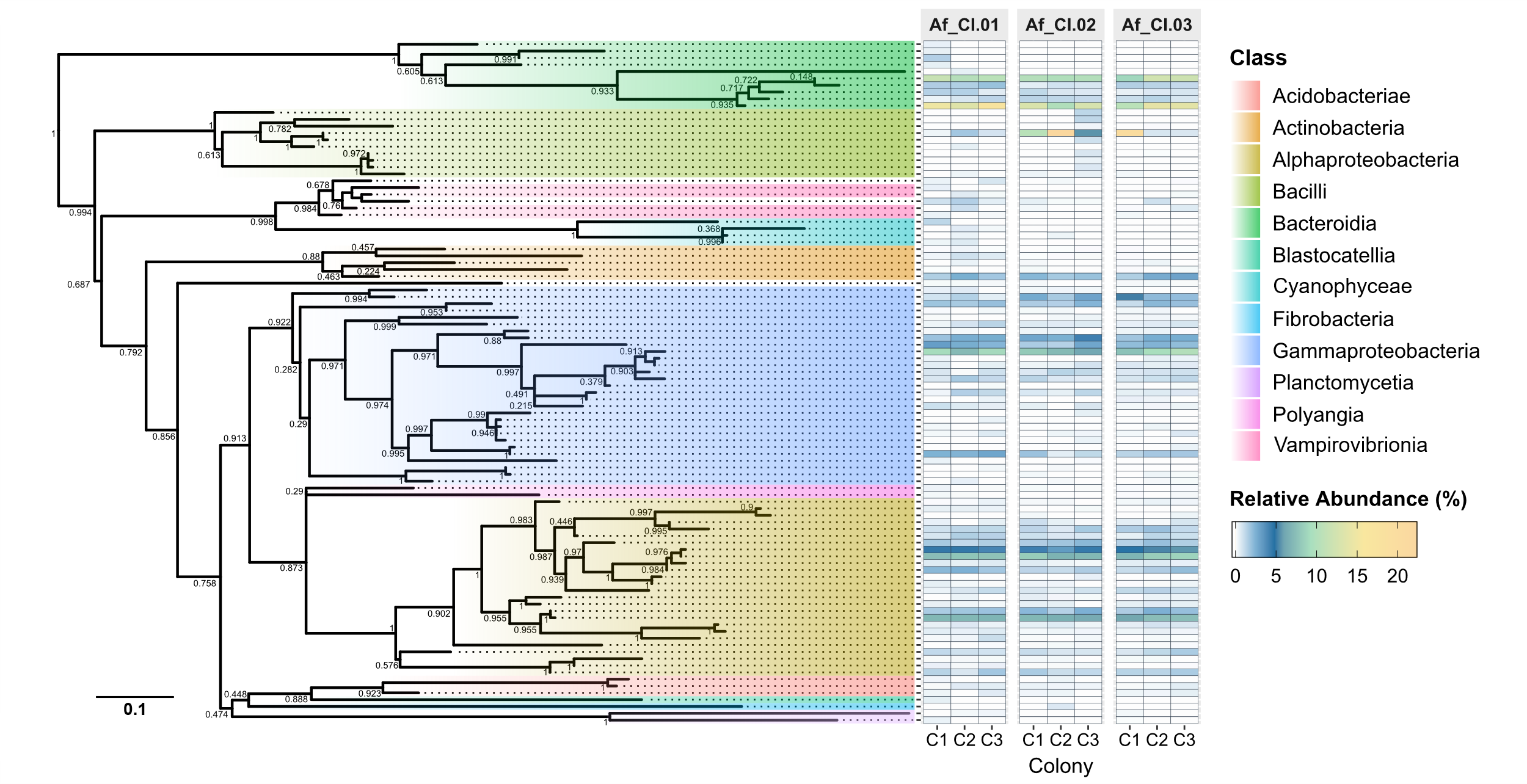


**Figure S3.** Maximum likelihood midpoint rooted tree of the 100 most abundant bacterial ASVs across the sample set (corresponding to ~95% of total relative abundance in each sample). The phylogenetic tree was constructed using the general time reversible model with the rate variation among sites described by a gamma distribution and the proportion of invariable sites (GTR + G + I model). Background colors indicate bacterial ASVs assigned at class level. Tree constriction was based on the region V4 of 16S rRNA gene sequences, applying 1000 bootstrap replications to estimate confidence. Bootstrap values are indicated above or below the branches. The scale bar indicates nucleotide substitutions per site. Heatmap shows the relative abundances of bacterial ASVs found in tested fungal clinical isolates, comparing three single spore colonies (C_1_, C_2_, C_3_). The color intensity shows the ASV percentage in each sample (note that in the color key the dark blue corresponds to 5%).


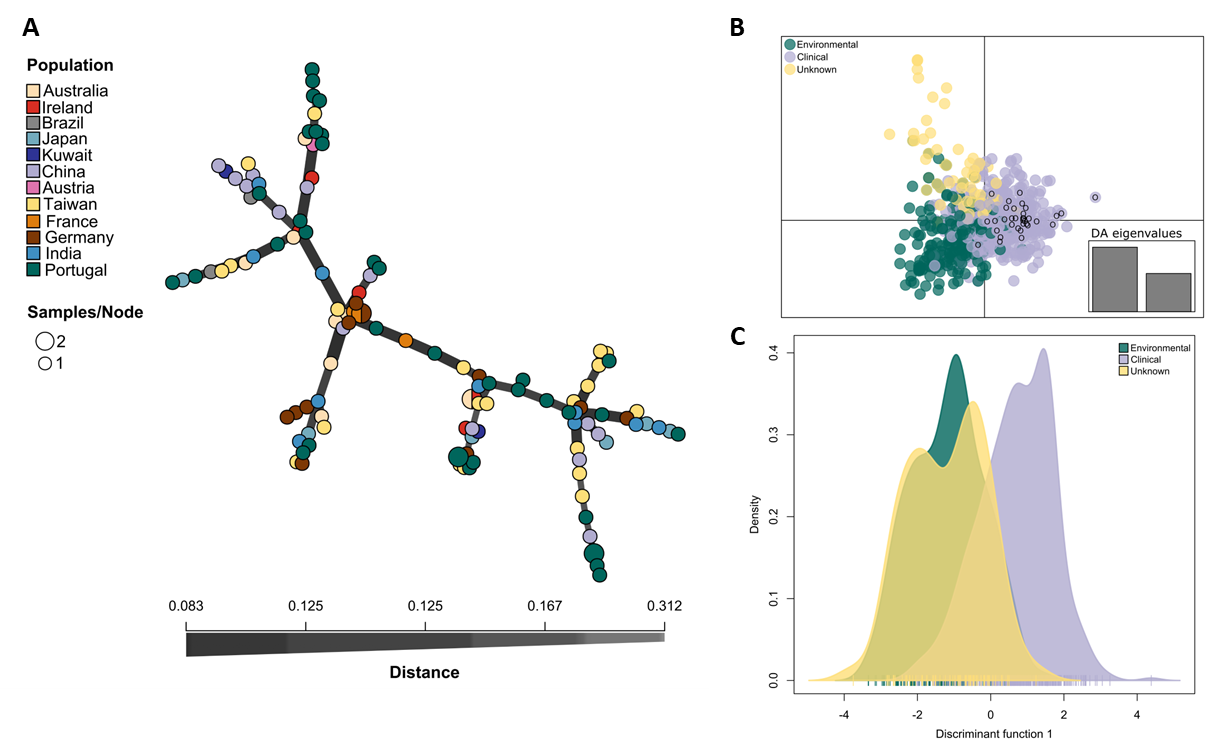


**Figure S4.** (A) Minimum spanning network of Aspergillus fumigatus isolates genotyped at nine microsatellite loci. The various genotypes of clinical isolates are represented by circles, with colors corresponding to their respective countries. The thickness of the connecting lines signifies the relatedness between the linked isolates, as determined by Bruvo’s distance using microsatellite genotyping data. Values approaching zero indicate identical isolates, whereas values nearing one indicate unrelated isolates. (B) Discriminant analysis of principal components (DAPC), clinical strains from Portugal analysed in this study are indicated with an open black circle above the respective spot in DAPC and are well spread amongst other clinical strains from around the world. (C) Density plot from a modified DAPC, illustrating the characteristics of the first discriminant function.

**Table S1.** Minimal inhibitory concentrations of each tested antifungal drug against each *Aspergillus fumigatus* strains. The strains were classified as resistant or susceptible according to breakpoint table v 10.0, 2020^#^ defined by European Committee on Antimicrobial Susceptibility Testing (EUCAST).

|  | **Amphotericin B** | | **Voriconazole** | | **Posaconazole** | | |
| --- | --- | --- | --- | --- | --- | --- | --- |
| **Isolate** | **MIC (mg·L^-1^)** | **Classification** | **MIC (mg·L^-1^)** | **Classification** | **MIC (mg·L^-1^)** | | **Classification** |
| **Af293** | 1.0 | Susceptible | 1.0 | Susceptible | 0.5 | Susceptible | |
| **Af_SI.00** | 1.0 | Susceptible | 1.0 | Susceptible | 0.5 | Susceptible | |
| **Af_CI.01** | 1.0 | Susceptible | 1.0 | Susceptible | 0.5 | Susceptible | |
| **Af_CI.02** | 1.0 | Susceptible | 1.0 | Susceptible | 0.5 | Susceptible | |
| **Af_CI.03** | 1.0 | Susceptible | 2.0 | Resistant | 1.0 | Resistant | |
| **Af_CI.06** | 1.0 | Susceptible | 1.0 | Susceptible | 0.5 | Susceptible | |
| **Af_CI.07** | 2.0 | Resistant | 1.0 | Susceptible | 0.5 | Susceptible | |
| **Af_CI.08** | 2.0 | Resistant | 2.0 | Resistant | 1.0 | Resistant | |
| **Af_CI.09** | 1.0 | Susceptible | 2.0 | Resistant | 1.0 | Resistant | |
| **Af_CI.11** | 2.0 | Resistant | 1.0 | Susceptible | 0.5 | Susceptible | |
| **Af_CI.12** | 1.0 | Susceptible | 1.0 | Susceptible | 1.0 | Resistant | |
| **Af_CI.13** | 1.0 | Susceptible | 1.0 | Susceptible | 1.0 | Resistant | |
| **Af_CI.14** | 1.0 | Susceptible | 2.0 | Resistant | 1.0 | Resistant | |
| **Af_CI.15** | 2.0 | Resistant | 2.0 | Resistant | 1.0 | Resistant | |
| **Af_CI.16** | 1.0 | Susceptible | 1.0 | Susceptible | 1.0 | Resistant | |
| **Af_CI.17** | 2.0 | Resistant | 2.0 | Resistant | 1.0 | Resistant | |
| **Af_CI.18** | 1.0 | Susceptible | 2.0 | Resistant | 1.0 | Resistant | |
| **Af_CI.19** | 2.0 | Resistant | 2.0 | Resistant | 0.5 | Susceptible | |
| **Af_CI.20** | 1.0 | Susceptible | 2.0 | Resistant | 1.0 | Resistant | |
| **Af_CI.22** | 2.0 | Resistant | 1.0 | Susceptible | 1.0 | Resistant | |
| **Af_CI.23** | 2.0 | Resistant | 1.0 | Susceptible | 1.0 | Resistant | |
| **Af_CI.24** | 2.0 | Resistant | 1.0 | Susceptible | 1.0 | Resistant | |
| **Af_CI.27** | 2.0 | Resistant | 1.0 | Susceptible | 1.0 | Resistant | |
| **Af_CI.28** | 1.0 | Susceptible | 1.0 | Susceptible | 1.0 | Resistant | |
| **Af_CI.30** | 1.0 | Susceptible | 1.0 | Susceptible | 1.0 | Resistant | |
| **Af_CI.31** | 2.0 | Resistant | 1.0 | Susceptible | 1.0 | Resistant | |
| **Af_CI.32** | 2.0 | Resistant | 1.0 | Susceptible | 1.0 | Resistant | |
| **Af_CI.34** | 2.0 | Resistant | 1.0 | Susceptible | 1.0 | Resistant | |
| **Af_CI.36** | 1.0 | Susceptible | 1.0 | Susceptible | 1.0 | Resistant | |
| **Af_CI.37** | 2.0 | Resistant | 1.0 | Susceptible | 1.0 | Resistant | |
| **Af_CI.38** | 2.0 | Resistant | 2.0 | Resistant | 0.5 | Susceptible | |
| **Af_CI.39** | 2.0 | Resistant | 1.0 | Susceptible | 0.5 | Susceptible | |
| **Af_CI.41** | 1.0 | Susceptible | 2.0 | Resistant | 1.0 | Resistant | |
| **Af_CI.42** | 1.0 | Susceptible | 1.0 | Susceptible | 0.5 | Susceptible | |
| **Af_CI.43** | 2.0 | Resistant | 2.0 | Resistant | 0.5 | Susceptible | |
| **Af_CI.44** | 1.0 | Susceptible | 1.0 | Susceptible | 0.5 | Susceptible | |
| **Af_CI.45** | 2.0 | Resistant | 2.0 | Resistant | 1.0 | Resistant | |
| **Af_CI.46** | 2.0 | Resistant | 2.0 | Resistant | 0.5 | Susceptible | |
| **Af_CI.47** | 1.0 | Susceptible | 1.0 | Susceptible | 1.0 | Resistant | |
| ^#^Arendrup MC, Friberg N, Mares M, Kahlmeter G, Meletiadis J, Guinea J, Andersen C, Arikan-Akdagli S, Barchiesi F, Chryssanthou E: How to interpret MICs of antifungal compounds according to the revised clinical breakpoints v. 10.0 European committee on antimicrobial susceptibility testing (EUCAST). *Clin Microbiol Infect* 2020, **26**(11):1464-1472. | | | | | | | |


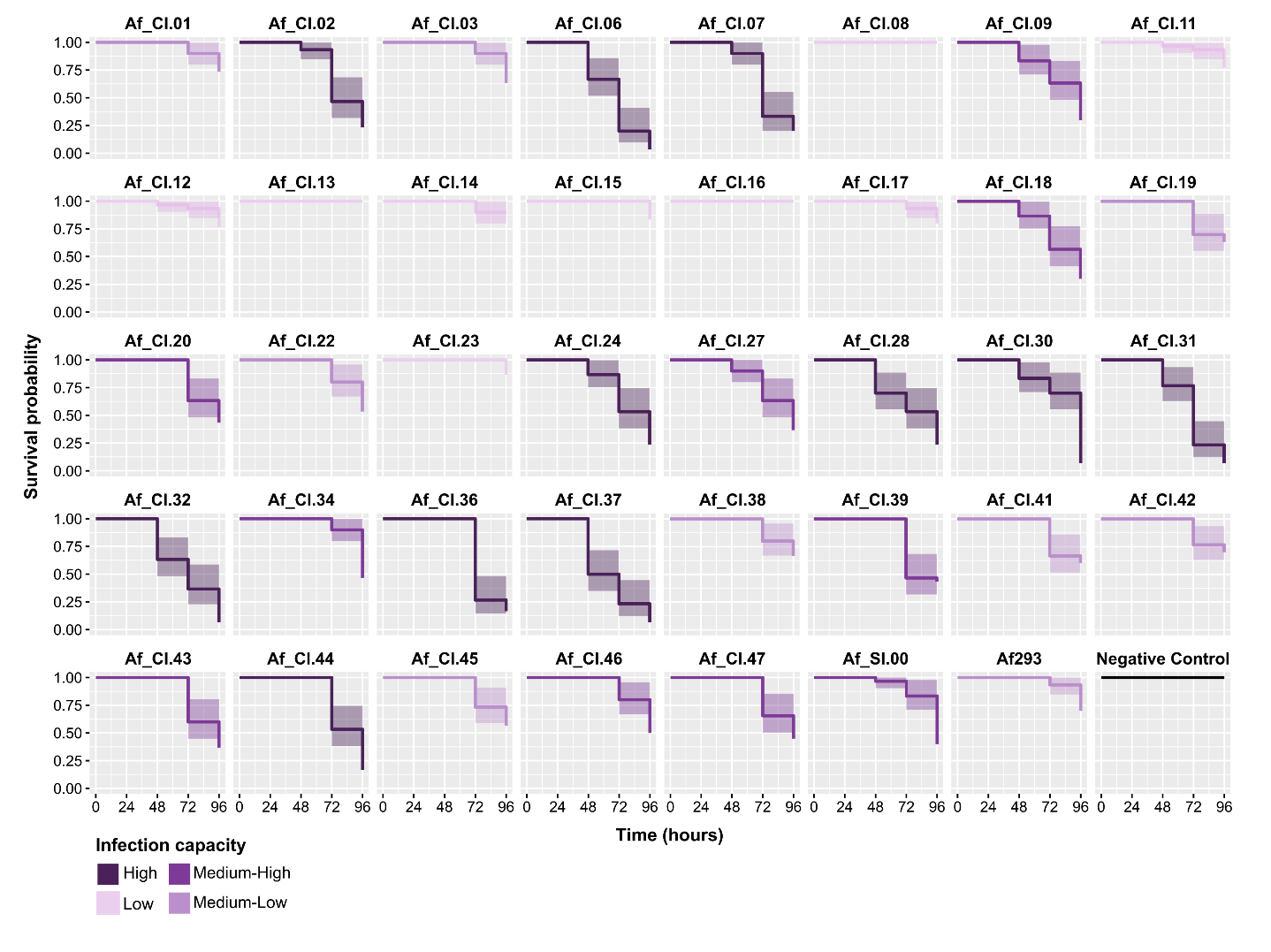


**Figure S5.** Infection capacity of each A. fumigatus clinical isolate and one soil isolate (Af_SI.00) using Galleria mellonella as infection model (n = 30). The infection capacity was assessed individually (infection with 10^7^ spores per larvae), showed by Kaplan-Meier survival curves discriminating the survival probability over 96 h. Negative control corresponds to injection of saline solution.

**Table S2.** Ratio and derived percentages of 18S/16S rDNA in spores (~10^8^ spores·mL^-1^) and 24-old mycelia of several *A. fumigatus* strains (n=8). Ratio_18S/16S_ = 2^(Ct18S rDNA−Ct16S rDNA)^ and the18S% = Ratio_18S/16S_ / (Ratio_18S/16S_ + 1) *100 and 16S% = 100 – 18S%^#^.

| Strain | 18S/16S Ratio | | Percentages (%) | | | |
| --- | --- | --- | --- | --- | --- | --- |
|  | **Mycelium** | **Spores** | **Mycelium** | | **Spores** | |
|  |  |  | **18S%** | **16S%** | **18S%** | **16S%** |
| Af_SI.00 | 164.7 | 73.1 | 99.4 | 0.6 | 98.7 | 1.4 |
| AF_CI.01 | 106.9 | 77.6 | 99.1 | 0.9 | 98.7 | 1.3 |
| AF_CI.02 | 91.6 | 134.8 | 98.9 | 1.1 | 99.3 | 0.7 |
| AF_CI.03 | 104.9 | 108.2 | 99.1 | 0.9 | 99.1 | 0.9 |
| AF_CI.06 | 108.4 | 61.7 | 99.1 | 0.9 | 98.4 | 1.6 |
| AF_CI.07 | 216.3 | 98.6 | 99.5 | 0.5 | 99.0 | 1.0 |
| AF_CI.08 | *n.d.* | 228.8 | *n.d.* | *n.d.* | 99.6 | 0.4 |
| AF_CI.12 | 65.5 | 287.2 | 98.5 | 1.5 | 99.7 | 0.4 |
| AF_CI.18 | 181.4 | 274.3 | 99.5 | 0.6 | 99.6 | 0.4 |
| *n.d.*, not done; AF.CI.018, after 48-h, formed a scarce mycelium, insufficient to extract DNA. Oligonucleotide pairs were: (i) 16S. CTCCTACGGGAGGCAGCACT (forward) and ATTACCGCGGCTGCTGG (reverse) and (ii) 18S. CGATAACGAACGAGACCT (forward) and AICCATTCAATCGGTAIT (reverse) using RT-qPCR (CFX96 Thermocycler. Bio-Rad). Each 10 µL reaction mixture included 5 µL of SsoFast Evagreen® Supermix (Bio-Rad). 0.5 µL of each primer (10 µM). 3 µL of PCR-quality water and 1 µL of template rDNA at a concentration of 10 ng/µL (96 well plates). The PCR conditions were as follows: enzyme activation at 95 ºC for 120 s; 35 cycles of denaturation at 94 ºC for 20 s. annealing at 64 ºC (16S) or 58 ºC (18S) for 30 s and extension at 68 ºC for 30 s. Melting curves from the RT-qPCR assays were examined to verify primer specificity. All assays were done using three biological replicates, and three to five technical replicates, besides adequate controls.  # Ostheim P, Alemu S, Tichý A, Sirak I, Davidkova M, Stastna MM, Kultova G, Schuele S, Paunesku T, Woloschak G: Examining potential confounding factors in gene expression analysis of human saliva and identifying potential housekeeping genes. Sci Rep 2022, 12(1):2312. | | | | | | |


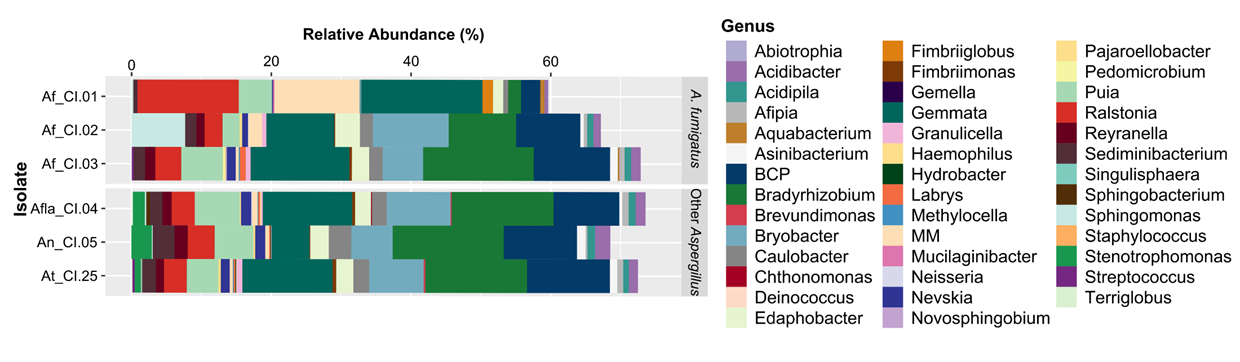


**Figure S6** – Stacked bar chart showing the relative abundance ASVs from the bacterial V3-V4 hypervariable region of 16S rRNA sequences, taxonomically classified at genus level. Showing the comparison between bacteriome found in 3 A. fumigatus clinical isolates (same as Figure 3) with three additional A. terreus (At_CI.25), A. niger (An_CI.05), and A. flavus (Afla_CI.04) clinical isolates. Low abundance taxa were deleted from the visualization. The order of bacterial genus in the legend is according with its position in the chart.


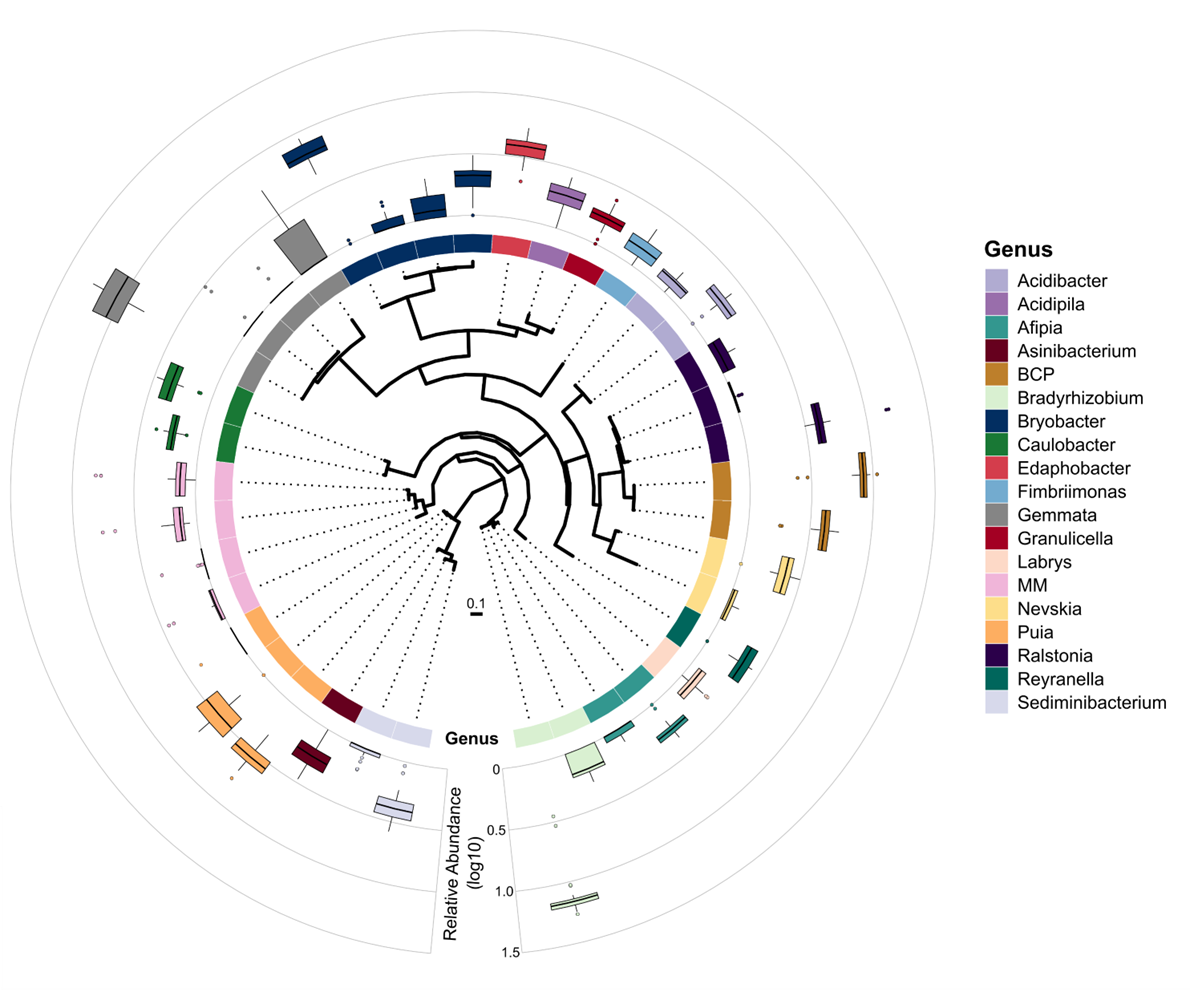


**Figure S7.** Phylogenetic tree of the core bacteriome. The scale bar indicates nucleotide substitutions per site. Log_10_ transformed relative abundances.


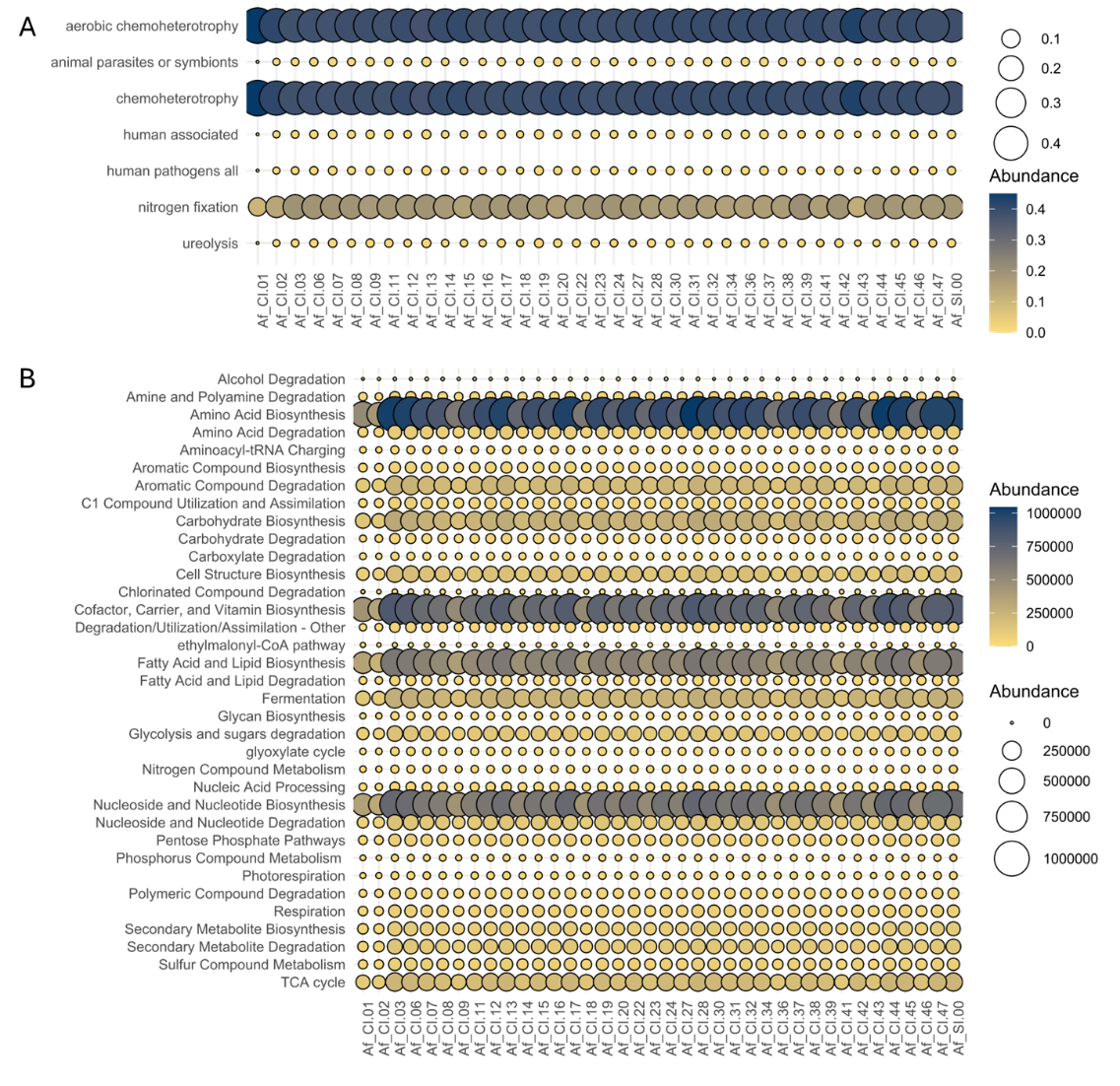


**Figure S8.** Prediction of the ecologically relevant functions of the *Aspergillus fumigatus* isolates bacteriome. Picrust2 prediction of the ecological functions of core bacteria (75% prevalence with at least 0.1% detection threshold) using the relative abundance ASVs from the bacterial V3-V4 hypervariable region of 16S rRNA sequences.


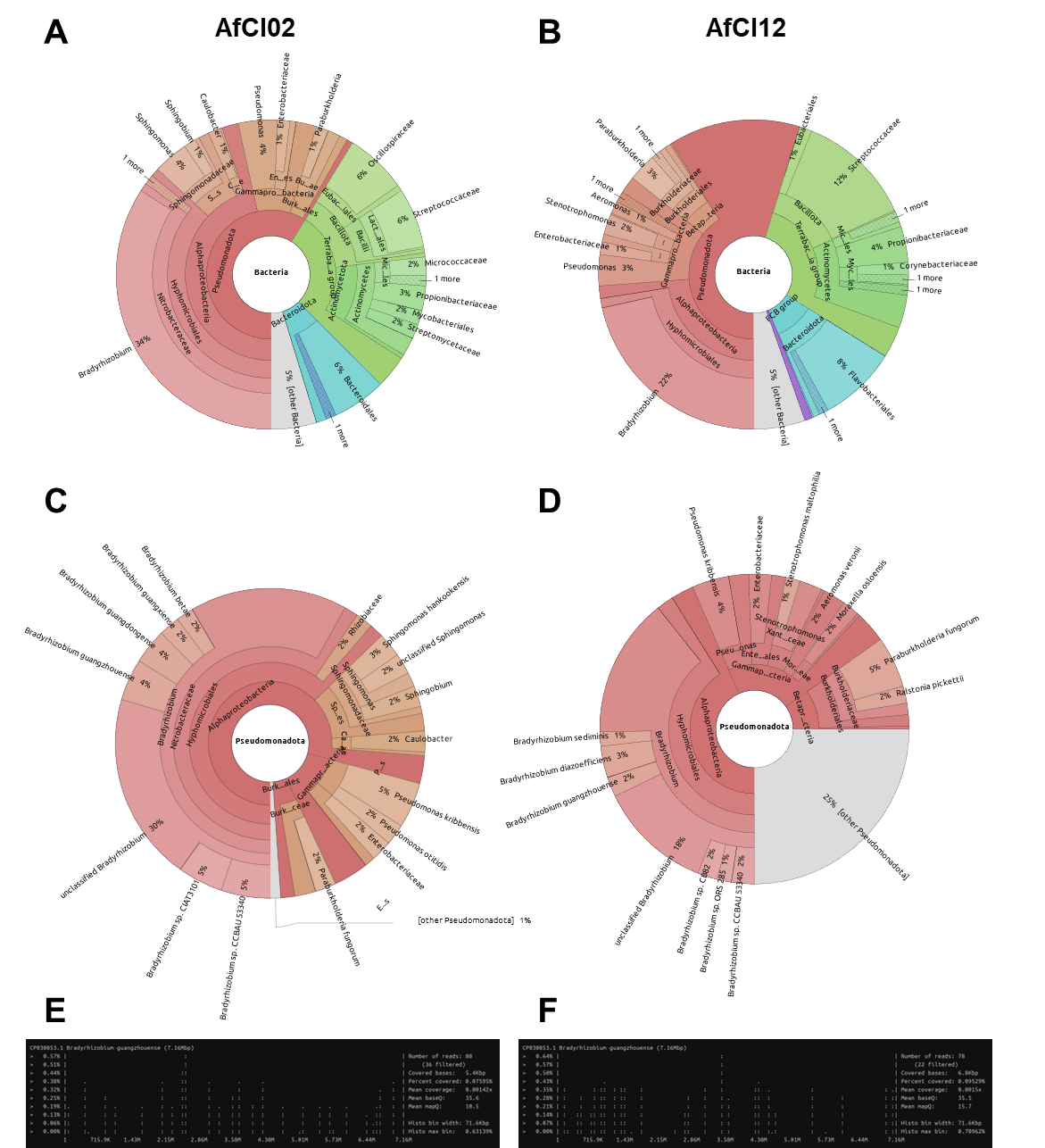


**Figure S9**. Metagenome analysis of whole genome sequencing reads from two clinical isolates. Highlight of reads matched to Bacteria (panel A and B; 466 and 619 counts respectively for Af_CI.02 and Af_CI.12), or Pseudomonadota (C and D) using Kraken, and genome coverage of reads aligned to *Bradyrhizobium guangzhouense* CCBAU 51670 (acc. no. CP030053) (E and F).


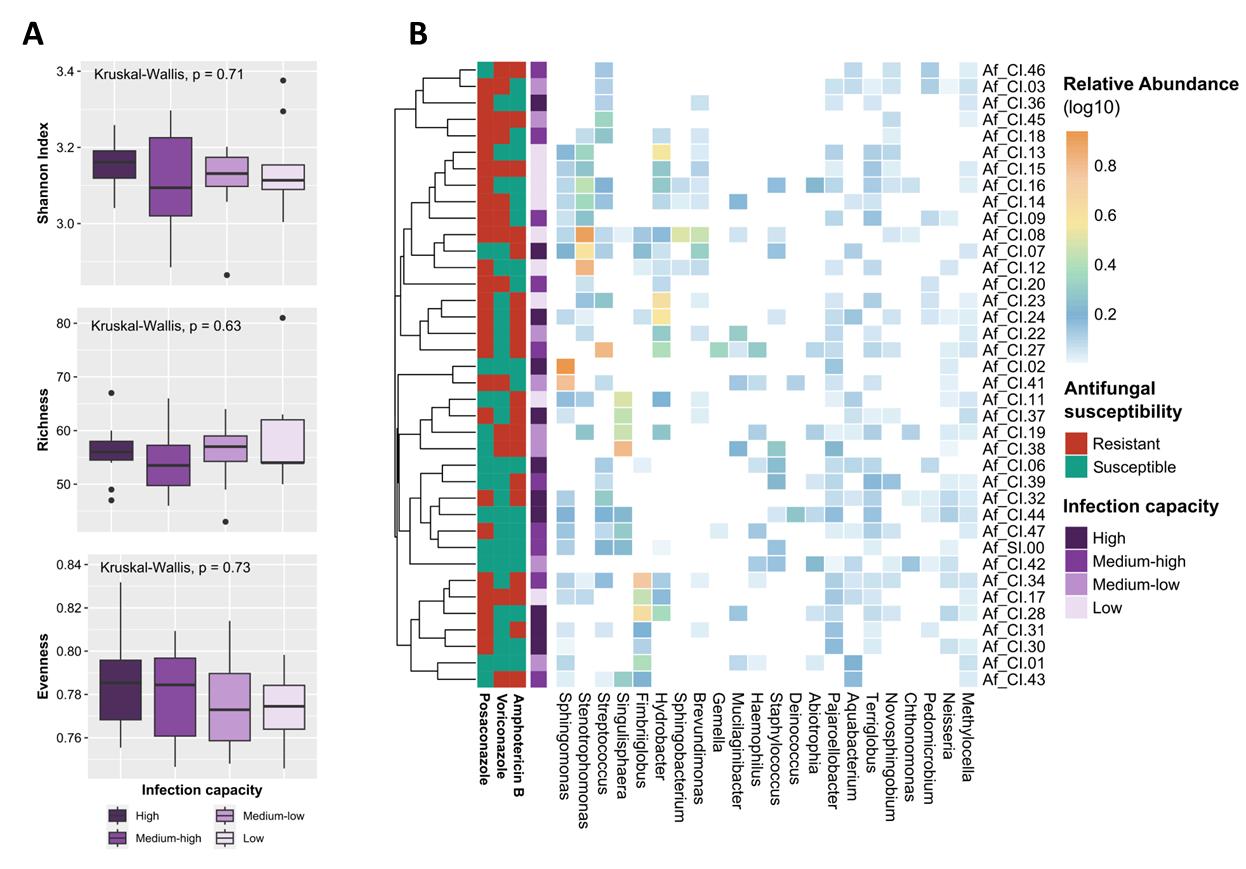


**Figure S10.** (A) Shannon diversity, richness, and evenness of the fungal bacteriome in different infection capacity groups. Kruskal-Wallis test was used to test for significant differences between groups. (B) Hierarchical clustering heat map of fungal bacteriome using Bray-Curtis distance, without core bacteriome.

Bolger, A. M., M. Lohse and B. Usadel (2014). "Trimmomatic: a flexible trimmer for Illumina sequence data." Bioinformatics **30**(15): 2114-2120.

Callahan, B. J., P. J. McMurdie and S. P. Holmes (2017). "Exact sequence variants should replace operational taxonomic units in marker-gene data analysis." Isme Journal **11**(12): 2639-2643.

Callahan, B. J., P. J. McMurdie, M. J. Rosen, A. W. Han, A. J. Johnson and S. P. Holmes (2016). "DADA2: High-resolution sample inference from Illumina amplicon data." Nat Methods **13**(7): 581-583.

Caporaso, J. G., C. L. Lauber, W. A. Walters, D. Berg-Lyons, C. A. Lozupone, P. J. Turnbaugh, N. Fierer and R. Knight (2011). "Global patterns of 16S rRNA diversity at a depth of millions of sequences per sample." Proc Natl Acad Sci U S A **108 Suppl 1**(Suppl 1): 4516-4522.

Danecek, P., J. K. Bonfield, J. Liddle, J. Marshall, V. Ohan, M. O. Pollard, A. Whitwham, T. Keane, S. A. McCarthy, R. M. Davies and H. Li (2021). "Twelve years of SAMtools and BCFtools." Gigascience **10**(2).

de Valk, H. A., J. F. Meis, I. M. Curfs, K. Muehlethaler, J. W. Mouton and C. H. Klaassen (2005). "Use of a novel panel of nine short tandem repeats for exact and high-resolution fingerprinting of Aspergillus fumigatus isolates." J Clin Microbiol **43**(8): 4112-4120.

Douglas, G. M., V. J. Maffei, J. R. Zaneveld, S. N. Yurgel, J. R. Brown, C. M. Taylor, C. Huttenhower and M. G. I. Langille (2020). "PICRUSt2 for prediction of metagenome functions." Nat Biotechnol **38**(6): 685-688.

Kamvar, Z. N., J. F. Tabima and N. J. Grunwald (2014). "Poppr: an R package for genetic analysis of populations with clonal, partially clonal, and/or sexual reproduction." PeerJ **2**: e281.

Louca, S., L. W. Parfrey and M. Doebeli (2016). "Decoupling function and taxonomy in the global ocean microbiome." Science **353**(6305): 1272-1277.

Lu, J., N. Rincon, D. E. Wood, F. P. Breitwieser, C. Pockrandt, B. Langmead, S. L. Salzberg and M. Steinegger (2022). "Metagenome analysis using the Kraken software suite." Nat Protoc **17**(12): 2815-2839.

Marti, J. M. (2019). "Recentrifuge: robust comparative analysis and contamination removal for metagenomics." PLoS Comput Biol **15**(4): e1006967.

Martins, C., D. Piontkivska, D. Mil-Homens, P. Guedes, J. M. P. Jorge, J. Brinco, C. Barria, A. C. F. Santos, R. Barras, C. Arraiano, A. Fialho, G. H. Goldman and C. Silva Pereira (2023). "Increased production of pathogenic, airborne fungal spores upon exposure of a soil mycobiota to chlorinated aromatic hydrocarbon pollutants." Microbiol Spectr **11**(4): e0066723.

McMurdie, P. J. and S. Holmes (2013). "Phyloseq: an R package for reproducible interactive analysis and graphics of microbiome census data." PLoS One **8**(4): e61217.

Morales, D. P., A. J. Robinson, A. C. Pawlowski, C. Ark, J. M. Kelliher, P. Junier, J. H. Werner and P. S. G. Chain (2022). "Advances and challenges in fluorescence in situ hybridization for visualizing fungal endobacteria." Front Microbiol **13**: 892227.

Murali, A., A. Bhargava and E. S. Wright (2018). "IDTAXA: a novel approach for accurate taxonomic classification of microbiome sequences." Microbiome **6**(1): 140.

Nomani, E., A. A. Al-Gheethi, N. K. Rahman, B. Tahlip, R. Mohamed and O. A. Kadir (2018). "Single spore isolation as a simple and efficient technique to obtain fungal pure culture." 4th International Conference on Civil and Environmental Engineering for Sustainability (Iconcees 2017) **140**.

Otto, C., P. F. Stadler and S. Hoffmann (2014). "Lacking alignments? The next-generation sequencing mapper segemehl revisited." Bioinformatics **30**(13): 1837-1843.

Quast, C., E. Pruesse, P. Yilmaz, J. Gerken, T. Schweer, P. Yarza, J. Peplies and F. O. Glockner (2013). "The SILVA ribosomal RNA gene database project: improved data processing and web-based tools." Nucleic Acids Res **41**(Database issue): D590-596.

Schliep, K. P. (2011). "Phangorn: phylogenetic analysis in R." Bioinformatics **27**(4): 592-593.

Sewell, T. R., J. Zhu, J. Rhodes, F. Hagen, J. F. Meis, M. C. Fisher and T. Jombart (2019). "Nonrandom distribution of azole resistance across the global population of aspergillus fumigatus." mBio **10**(3).

Subcommittee on Antifungal Susceptibility Testing of the, E. E. C. f. A. S. T. (2008). "EUCAST technical note on the method for the determination of broth dilution minimum inhibitory concentrations of antifungal agents for conidia-forming moulds." Clin Microbiol Infect **14**(10): 982-984.

Thijs, S., M. Op De Beeck, B. Beckers, S. Truyens, V. Stevens, J. D. Van Hamme, N. Weyens and J. Vangronsveld (2017). "Comparative evaluation of four bacteria-specific primer pairs for 16S rRNA gene surveys." Front Microbiol **8**: 494.

Weisburg, W. G., S. M. Barns, D. A. Pelletier and D. J. Lane (1991). "16S ribosomal DNA amplification for phylogenetic study." J Bacteriol **173**(2): 697-703.

Woo, P. C., A. H. Ngan, H. K. Chui, S. K. Lau and K. Y. Yuen (2010). "Agar block smear preparation: a novel method of slide preparation for preservation of native fungal structures for microscopic examination and long-term storage." J Clin Microbiol **48**(9): 3053-3061.
